# Considering discrepancy when calibrating a mechanistic electrophysiology model

**DOI:** 10.1098/rsta.2019.0349

**Published:** 2020-05-25

**Authors:** Chon Lok Lei, Sanmitra Ghosh, Dominic G. Whittaker, Yasser Aboelkassem, Kylie A. Beattie, Chris D. Cantwell, Tammo Delhaas, Charles Houston, Gustavo Montes Novaes, Alexander V. Panfilov, Pras Pathmanathan, Marina Riabiz, Rodrigo Weber dos Santos, John Walmsley, Keith Worden, Gary R. Mirams, Richard D. Wilkinson

**Affiliations:** 1 Computational Biology and Health Informatics, Department of Computer Science, University of Oxford, Oxford, UK; 2 MRC Biostatistics Unit, University of Cambridge, Cambridge, UK; 3 Centre for Mathematical Medicine and Biology, School of Mathematical Sciences, University of Nottingham, Nottingham, UK; 4 Department of Bioengineering, University of California San Diego, La Jolla, CA, USA; 5 Systems Modeling and Translational Biology, GlaxoSmithKline R&D, Stevenage, UK; 6 ElectroCardioMaths Programme, Centre for Cardiac Engineering, Imperial College London, London, UK; 7 CARIM School for Cardiovascular Diseases, Maastricht University, Maastricht, The Netherlands; 8 Graduate Program in Computational Modeling, Universidade Federal de Juiz de Fora, Juiz de Fora, Brazil; 9 Department of Physics and Astronomy, Ghent University, Ghent, Belgium; 10 Laboratory of Computational Biology and Medicine, Ural Federal University, Ekaterinburg, Russia; 11 US Food and Drug Administration, Center for Devices and Radiological Health, Office of Science and Engineering Laboratories, Silver Spring, MD, USA; 12 Department of Biomedical Engineering King’s College London and Alan Turing Institute, London, UK; 13 James T. Willerson Center for Cardiovascular Modeling and Simulation, Oden Institute for Computational Engineering and Sciences, The University of Texas at Austin, Austin, TX, USA; 14 Dynamics Research Group, Department of Mechanical Engineering, University of Sheffield, Sheffield, UK; 15 School of Mathematics and Statistics, University of Sheffield, Sheffield, UK

**Keywords:** model discrepancy, uncertainty quantification, cardiac model, Bayesian inference

## Abstract

Uncertainty quantification (UQ) is a vital step in using mathematical models and simulations to take decisions. The field of cardiac simulation has begun to explore and adopt UQ methods to characterize uncertainty in model inputs and how that propagates through to outputs or predictions; examples of this can be seen in the papers of this issue. In this review and perspective piece, we draw attention to an important and under-addressed source of uncertainty in our predictions—that of uncertainty in the model structure or the equations themselves. The difference between imperfect models and reality is termed *model discrepancy*, and we are often uncertain as to the size and consequences of this discrepancy. Here, we provide two examples of the consequences of discrepancy when calibrating models at the ion channel and action potential scales. Furthermore, we attempt to account for this discrepancy when calibrating and validating an ion channel model using different methods, based on modelling the discrepancy using Gaussian processes and autoregressive-moving-average models, then highlight the advantages and shortcomings of each approach. Finally, suggestions and lines of enquiry for future work are provided.

This article is part of the theme issue ‘Uncertainty quantification in cardiac and cardiovascular modelling and simulation’.

## 1. Introduction

This perspective paper discusses the issue of model discrepancy—the difference between a model’s predictions and reality. The concepts and issues we highlight are applicable to any modelling situation where governing equations are approximations or assumptions; thus our perspective paper is intended for computational, mathematical and statistical modellers within many other fields as well as within and outside biological modelling. The focus of our examples is cellular cardiac electrophysiology, a well-developed area of systems biology [1].

### (a) Cardiac modelling

Cardiac models are typically a collection of mathematical functions governed by systems of ordinary and/or partial (when spatial dimensions are considered) differential equations, integrated using computational techniques, which produce responses that depend on the model inputs. Inputs can include model parameters, initial conditions, boundary conditions, and cellular, tissue or whole organ geometrical aspects. Inputs which have physiological meaning can sometimes be obtained by direct measurement, while others may need to be estimated via an indirect calibration procedure using experimental data. There are many examples of such cardiac models, at a variety of different scales, discussed in the papers of this special issue.

Mathematical modelling and computational simulation has been remarkably successful at providing insights into cardiac physiological mechanisms at cellular, tissue and whole organ scales [2–7]. In the majority of these quantitative efforts, models are derived based on simplified representations of complex biophysical systems and use *in vitro* and *in vivo* experimental data for calibration and validation purposes. Quantitative cardiac models have been a crucial tool for basic research for decades, and more recently have begun to transition into safety-critical clinical and pharmaceutical development applications [8–12]. The use of cardiac mathematical models in such applications will require high levels of credibility in the predictive model outputs, as well as an accurate quantification of the uncertainty in these predictions.

Parameters in cardiac models are often uncertain, mainly due to measurement uncertainty and/or natural physiological variability [13]. Thus, uncertainty quantification (UQ) methods are required to study uncertainty propagation in these models and help to establish confidence in model predictions. Parametric UQ is the process of determining the uncertainty in model inputs or parameters, and then estimating the resultant uncertainty in model outputs, thus testing the robustness of model predictions given our uncertainty in their inputs, and has been applied to a variety of cardiac models [14–19].

Another major source of uncertainty in modelling is uncertainty in the model structure, i.e. the form of the governing equations. There is always a difference between the imperfect model used to approximate reality, and reality itself; this difference is termed model discrepancy. Assessment of the robustness of model predictions given our uncertainty in the model structure, and methods to characterize model discrepancy, has received relatively little attention in this field (and mathematical/systems biology more generally). We have found only two published explicit treatments of discrepancy in cardiac electrophysiology models, in papers by Plumlee *et al.* [20,21]. In these studies, the assumption that ion channel rate equations follow an explicit form (such as that given, as we will see later, by equation (3.5)) was relaxed, and rates were allowed to be Gaussian processes (GPs) in voltage. A two-dimensional GP (in time and voltage) was then also added to the current prediction to represent discrepancy in current for a single step to any fixed voltage.

### (b) Notation and terminology

Before discussing model discrepancy in detail, we introduce some notation and terminology. As the concepts introduced here are intended to be understood not just by a cardiac modelling audience, we provide a non-exhaustive list of terminology we have encountered in different fields to describe useful concepts relating to calibration and model discrepancy (and mathematical/computational modelling in general) in table 1.

**Table 1.** Terminology used in different fields to refer to inverse problem concepts.

concept	terminologies	
fitting parameters in a given model to data	calibration	inverse problem
	parameter inference	parameter identification
	parameter estimation	parameter tuning
	parameter fitting	parameter optimization
	model matching/fitting	
do data from given experiment provide sufficient information to identify the model parameters?	parameter identifiability	practical identifiability
	structural identifiability	well-posedness
altering experiments to improve parameter identifiability	experimental design	protocol design
choosing model equations	model selection	model choice
	system identification	
the difference between model and reality	model discrepancy	model uncertainty
	model misspecification	model mismatch
	model inadequacy	model form error
	structural error	model structure error
the observable measurements (data)	observables	observable outputs
	quantities of interest (QoIs)	
a simplified version of the simulator/model	surrogate model	metamodel
	proxy	emulator
	look-up table	
checking the performance of the fitted model	validation	certification
	qualification	performance estimation

Here, we delve into some of those concepts in more detail. Suppose a physiological system is modelled as *y* = *f*(**θ**, *u*), where *f* represents all governing equations used to model the system (also referred to as model form or model structure), **θ** is a vector of parameters characterizing the system, and *u* are known externally applied conditions or control variables applied in the particular experimental procedure. In a cardiac modelling context, these might represent a stimulus protocol, a drug concentration or the applied voltage protocol in a simulated voltage-clamp experiment. In general, **θ** = {**θ**_*D*_, **θ**_*C*_}, where values of **θ**_*D*_ are directly measured, and where values of **θ**_*C*_ are determined by calibration using the model *f*. Here, for simplicity of exposition, we assume **θ**_*D*_ is fixed (and known) and **θ** = **θ**_*C*_.

We can distinguish between external conditions used for calibration, validation and prediction (that is, the application of the model, or context of use (CoU)), *u*_*C*_, *u*_*V*_, *u*_*P*_, say. Suppose we have experimental data *Y*_*C*_ for calibration and *Y*_*V*_ for validation. A typical workflow, without UQ, is

— **Calibration**: estimate $\hat{\theta }={\text{argmin}}_{\theta \in \Theta }d_{C}(f(\theta ,u_{C}),Y_{C})$, using some calibration distance function *d*_*C*_( · , · ) (e.g. a vector norm: $d_{C}(x,y)=||x-y||$), and some subset of parameter space **Θ**;
— **Validation**: compare $y_{V}=f(\hat{\theta },u_{V})$ against *Y*_*V*_, either qualitatively or using a suitable validation distance $d_{V}(f(\hat{\theta },u_{V}),Y_{V})$;
— **Context of use**: compute $Y_{P}=f(\hat{\theta },u_{P})$, or some quantity derived from this, to learn about the system or to make a model-based decision.

The calibration stage has many different names (table 1).

In practice, there a number of reasons why we cannot infer parameter values with certainty. The most commonly considered situation is when the link between the data and the model output is stochastic, e.g. because of measurement error on *Y*_*C*_ or because of model discrepancy. Computing the uncertainty about **θ** based on noisy data *Y*_*C*_ is referred to as ‘inverse UQ’, and requires a statistical model of the experimental data to be specified. For example, when considering measurement error, a common choice is to assume independent identically distributed zero-mean Gaussian errors on all data points, in which case (neglecting model discrepancy; see later) our model for the data is

1.1 $$Y_{C}=f(\theta ,u_{C})+\epsilon ,$$

with $\epsilon ={\left(\epsilon_{1},\epsilon_{2},\ldots \right)}^{⊤}$, where $\epsilon_{i}∼N(0,\sigma^{2})$. There are many different approaches to solving inverse UQ problems (e.g. [22,23]), most of which are based on inferring probability distributions to describe the relative likelihood that each different parameter set is consistent with the available data. Though a number of different methods to solve inverse UQ problems have been applied in cardiac electrophysiology [13], the most common is a Bayesian approach, which combines prior information about the parameters, *π*(**θ**), with the probability of observing the data given each parameter *π*(*Y*_*C*_|**θ**) (referred to as the likelihood of *θ*), to find a posterior distribution over the parameters

1.2 $$\pi (\theta |Y_{C})=\frac{\pi (Y_{C}|\theta )\pi (\theta )}{\pi (Y_{C})}.$$

For an introduction to Bayesian methods, see [24,25]. For the i.i.d. Gaussian error model (equation (1.1)), the likelihood is given by

1.3 $$\pi (Y_{C}|\theta )={\left(2\pi \sigma^{2}\right)}^{-n/2}\exp\left(-\frac{||Y_{C}-f(\theta ,u_{C})|{|}_{2}^{2}}{2\sigma^{2}}\right),$$

where $||x|{|}_{2}^{2}=\sum_{i}x_{i}^{2}$, and *n* is the number of data points.

Another potential source of uncertainty about **θ** can occur when the parameter varies across the (or a) population. Estimating population variability in **θ** requires multiple *Y*_*C*_ recordings, $\left\{Y_{C}^{\left(1\right)},Y_{C}^{\left(2\right)},\ldots \right\}$. Multilevel or hierarchical models can then be used: we assume the parameters for population *i* are drawn from some distribution **θ**^(*i*)^ ∼ *π*(**θ**|*ψ*), and infer the population parameters *ψ*, see [26].

Once uncertainty in **θ** (given the data) has been determined, the impact of this uncertainty on validation simulations *Y*_*V*_ or CoU simulations *Y*_*P*_ can be computed by propagating the uncertainty through the model *f* in the validation/CoU simulations, e.g.

$$\pi (Y_{P}|Y_{C})=\int \pi (Y_{P}|\theta )\pi (\theta |Y_{C})\text{d}\theta .$$

This is referred to as ‘uncertainty propagation’ or the ‘posterior predictive distribution’. Uncertainty in the prediction of *Y*_*V*_ helps provide a more informed comparison to the observed validation data (especially if experimental error in *Y*_*V*_ is also accounted for). Uncertainty in *Y*_*P*_ enables a more informed model-based decision-making process.

### (c) Model discrepancy

UQ as outlined above does not account for the fact that the model is always an imperfect representation of reality, due to limited understanding of the true data-generating mechanism and perhaps also any premeditated abstraction of the system. The model discrepancy is the difference between the model and the true data-generating mechanism, and its existence has implications for model selection, calibration and validation, and CoU simulations.

For calibration, the existence of model discrepancy can change the meaning of the estimated parameters. If we fail to account for the model discrepancy in our inference, our parameter estimates, instead of being physically meaningful quantities, will have their meaning intimately tied to the model used to estimate them (we end up estimating ‘pseudo-true’ values; see §2c). The estimated parameter values depend on the chosen model form, and the uncertainty estimates obtained during inverse parameter UQ tell us nothing about where the true value is (only how confident we are about the pseudo-true values). In other words, there is no guarantee the obtained **θ** will match true physiological values of any parameters that have a clear physiological meaning.

We can try to restore meaning to the estimated parameters by including a term to represent the model discrepancy in our models. Validation, in particular, provides an opportunity for us to identify possible model discrepancy. In many cases, validation, rather than being considered as an activity for confirming a ‘model is correct’, is better considered as a method for estimating the model discrepancy. To maximize the likelihood that the validation can discern model discrepancy, the validation data should ideally be ‘far’ from the calibration data, and as close to the CoU as possible.

## 2. A motivating example of discrepancy

To illustrate the concept of model discrepancy and some of its potential consequences, we have created a cardiac example inspired by previous work [27], using mathematical models of the action potential (AP) of human ventricular cells. These models have a high level of electrophysiological detail, including most of the major ionic currents as well as basic calcium dynamics, and have been used to study reentrant arrhythmias. We assume that the Ten Tusscher *et al.* ventricular myocyte electrophysiology model [28] (Model T) represents the ground truth, and use this model to generate data traces in three different situations: for *calibration* data we use the AP under 1 Hz pacing; to generate *validation* data we use 2 Hz pacing; and for *context of use* (CoU) data we use 1 Hz pacing with the 75% *I*_Kr_ block (*g*_*Kr*_ multiplied by a scaling factor of 0.25).

To illustrate the problem of fitting a model under model discrepancy, we assume we do not know the ground truth model and instead fit an alternative model, the Fink *et al.* model [29] (Model F), to the synthetic data generated from Model T. Both models F and T were built for human ventricular cardiomyocytes, with Model F being a modification of Model T that improves the descriptions of repolarizing currents, especially of the hERG (or *I*_Kr_) channel (which is a major focus for safety pharmacology). A comparison of the differences in the current kinetics between the two models is shown in figure 1, and the model equations are given in electronic supplementary material, §S1. Only five currents have kinetics that vary between the two models, and, importantly, no currents or compartments are missing (unlike when attempting to fit a model to real data).

**Figure 1.**
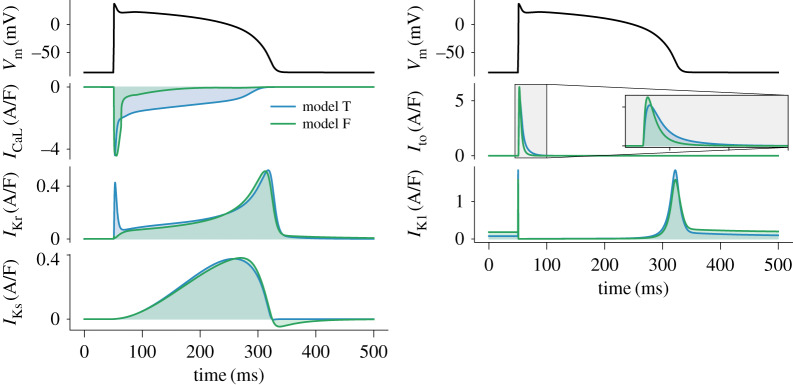
A comparison of the ten Tusscher (Model T [28], blue) and Fink (Model F [29], green) kinetics. These currents are voltage-clamp simulations under the same action potential clamp (shown in the top row panels). Only those currents with different kinetics are shown; the kinetics of *I*_Na_, *I*_NaCa_ and *I*_NaK_ are identical in both models. Two of the gates in *I*_CaL_ are identical in the two models, one gate has a different formulation, and Model F has one extra gate compared to Model T. The two models use different formulations for *I*_Kr_ (*I*_Kr_ activates during depolarization in Model T but not Model F), different parametrizations of the kinetics for *I*_Ks_ and *I*_to_, and different equations for *I*_K1_ steady state. Currents are normalized in this plot by minimizing the squared-difference between the two models’ currents such that we emphasize the differences in kinetics rather than the conductances (which are rescaled during the calibration). Only *I*_CaL_ shows what we would typically consider to be a large difference in repolarization kinetics, with the rest of the currents apparently being close matches between Model T and Model F. (Online version in colour.)

In this example, the control variables are the stimulus current and *I*_Kr_ block, the model outputs are the membrane voltage, and the parameters of interest are the maximum conductance/current density of the ionic currents. We use Model T to generate synthetic current-clamp experiments by simulating the different protocols (control variables) then adding i.i.d. Gaussian noise $∼N(0,\sigma^{2})$ to the resulting voltage traces (model outputs), with *σ* chosen to be 1 mV. We use the calibration data (1 Hz pacing) to estimate eight maximal conductance/current density parameters for *I*_Na_, *I*_CaL_, *I*_Kr_, *I*_Ks_, *I*_to_, *I*_NaCa_, *I*_K1_ and *I*_NaK_ using Model F. We will investigate whether the calibrated Model F makes accurate predictions in the validation and CoU situations (using the parameter estimates from the calibration data, as is commonly done in electrophysiology modelling [30–34]). The code to reproduce all of the results in this paper are available at https://github.com/CardiacModelling/fickleheart-method-tutorials.

### (a) Model calibration

We calibrate the model using a train of five APs stimulated under a 1 Hz pacing protocol as the calibration data. Before attempting to do this fitting exercise, the appropriately sceptical reader might ask whether we are attempting to do something sensible. Will we get back information on all the parameters we want, or will we just find one good fit to the data among many equally plausible ones, indicating non-identifiability of the parameters?

To address these questions, we first look at inferring the parameters of the original Model T (as well as inferring the noise model parameter, *σ*). We use equation (1.1) with Gaussian noise giving the likelihood in equation (1.2), together with a uniform prior distribution from 0.1 × to 10 × the original parameters of Model T. We take two different approaches to calibration. Firstly, we find a point estimate using a global optimization algorithm [35] to find the optimal model parameters (with no estimate of uncertainty). Secondly, we approximate the full posterior distribution using Markov chain Monte Carlo (MCMC). All inference is done using an open-source Python package, PINTS [36], and simulations are performed in Myokit [37].

The results are shown in electronic supplementary material, figure S1. This exercise results in a narrow plausible distribution of parameters very close to the ones that generated the data, and we conclude that the model parameters are identifiable with the given data. Additionally, electronic supplementary material, figure S1 shows that when using samples of these distributions to make predictions, all of the forward simulations are very closely grouped around the synthetic data for the *I*_Kr_ block CoU.

We now attempt the fitting exercise using Model F (i.e. the misspecified model). The fitted model prediction (using the maximum a posteriori (MAP) parameter estimate) is shown in figure 2*a*. The agreement between the calibrated model output and the synthetic data would be considered excellent if these were real experimental data. Therefore, it is tempting to conclude that this calibrated model gives accurate predictions, and that the model discrepancy is minor. But can we trust the predictive power of the model in other scenarios based solely on the result we see in figure 2*a*?

**Figure 2.**
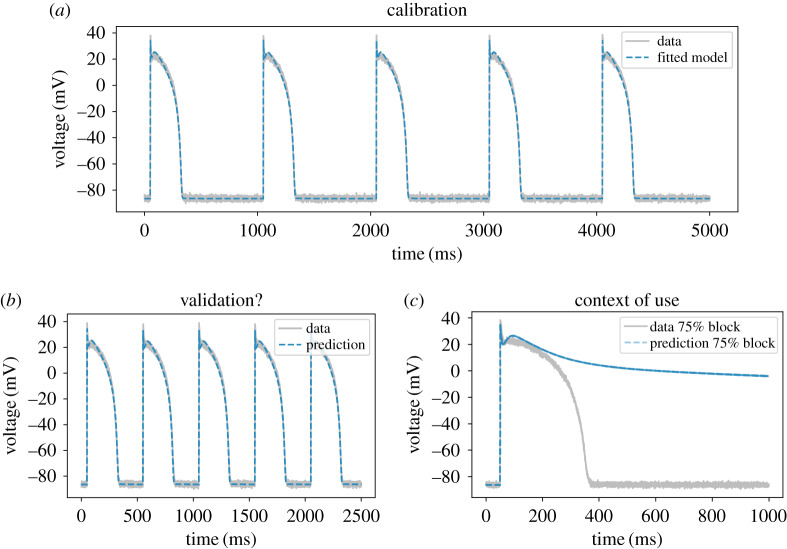
Model F fitting and validation results. (*a*) Model F is fitted to the synthetic data (generated from Model T), using five action potentials recorded under a 1 Hz pacing protocol. The calibrated Model F (blue dashed line) shows an excellent fit to the calibration data (grey solid line). (*b,c*) Model F predctions for validation and context of use (CoU) data. (*b*) The calibrated Model F predictions closely matches the validation data (2 Hz pacing), giving a (false) confidence in the model performance. (*c*) Notably, Model F gives catastrophic predictions for the *I*_Kr_ block (CoU) experiments (suggesting the validation data are not an appropriate test given the intended model use). The posterior predictions are model predictions made using parameter values sampled from the posterior distribution (figure 3); here, 200 samples/predictions are shown, but they overlay and are not distinguishable by eye. (Online version in colour.)

**Figure 3.**
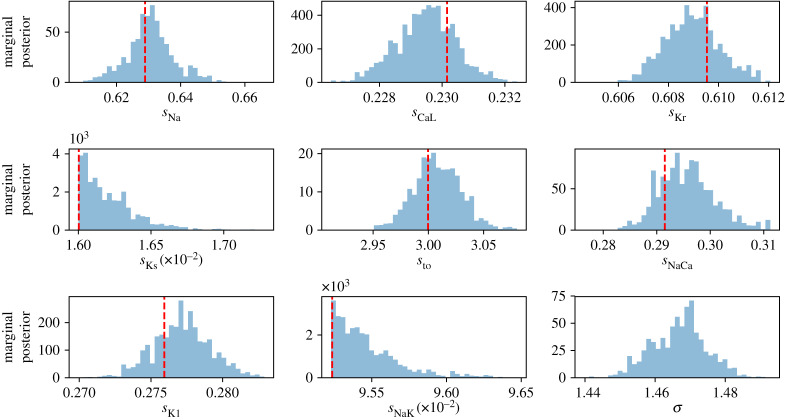
Marginals of the posterior distribution of the Model F parameters, in terms of scaling factors for the conductances in Model T (*s*_*i*_ = *g*^Model^~F_*i*_/*g*^Model^~T_*i*_). Values of 1 would represent the parameters of Model T that generated the data; note that none of the inferred parameters for Model F are close 1. The red dashed lines indicate the result of the global optimization routine. Two of these parameters, *S*_*Ks*_ and *S*_*NaK*_, have distributions hitting the lower bound that was imposed by the prior, indicating that the calibration process is attempting to make them smaller than 10% of the original Model F parameter values. (Online version in colour.)

### (b) Discrepant model predictions

Interestingly, the calibrated Model F gives very accurate predictions for the 2 Hz pacing validation protocol (data that are not used to estimate the parameters), as shown in figure 2*b*. Such rate-adaptation predictions are used commonly as validation evidence for AP models. At this stage, we may be increasingly tempted to conclude that we have a good model of this system’s electrophysiology.

But if one now uses the model to predict the effect of drug-induced *I*_Kr_ block, the catastrophic results are shown in the bottom right panel of figure 2. The calibrated Model F fails to repolarize, completely missing the true *I*_Kr_ block response of a modest AP duration prolongation. This example highlights the need for thorough validation and the CoU-dependence of model validation, but also the difficulty in choosing appropriate validation experiments.

We can also quantify the uncertainty in parameter estimates and predictions while continuing to ignore the discrepancy in Model F’s kinetics. Again, we use equation (1.2) together with a uniform prior to derive the posterior distribution of the parameters. The marginals of the posterior distribution, estimated by MCMC, and the point estimates obtained by optimization are shown in figure 3. The posterior distribution is very narrow (note the scale), which suggests that we can be confident about the parameter values. The resulting posterior predictions, shown in figure 2*c*, give a very narrow bound. By ignoring model discrepancy we have become highly (and wrongly) certain that the catastrophically bad predictions are correct.

It is worth noting that all of the issues above arise from the fact that the model discrepancy was ignored during calibration. In the scenario of no model discrepancy, i.e. when fitting Model T to the data, none of the issues above occurred, as shown in electronic supplementary material, figure S1.

To conclude our motivation of this paper, we can see that neglecting discrepancy in the model’s equations is dangerous and can lead to false confidence in predictions for a new context of use. We discuss methods that have been suggested to remedy this in §3.

### (c) A statistical explanation

To understand what happens when we fit an incorrect model to data, let us first consider the well-specified situation where the data generating process (DGP) has probability density function (pdf) *g*(*y*), and for which we have data *y*_*i*_ ∼ *g*( · ) for *i* = 1, …, *n*. Then suppose we are considering the models P={p(y∣θ):θ∈Θ}, i.e. a collection of pdfs parameterized by unknown parameter **θ**. If the DGP *g* is in P, i.e. we have a well-specified model so that for some **θ**_0_ ∈ **Θ**, we have *g*( · ) = *p*( · |**θ**_0_), then asymptotically, as we collect more data (and under suitable conditions [38]), the maximum-likelihood estimator converges to the true value **θ**_0_ almost surely

$${\hat{\theta }}_{n}={\text{argmax}}_{\theta }\sum_{i=1}^{n}\log p(y_{i}|\theta )⟶\theta_{0},\;\text{ almost surely as }\;n⟶\infty ,$$

or equivalently $p(\cdot |{\hat{\theta }}_{n})$ converges to *g*( · ). Similarly, for a Bayesian analysis (again under suitable conditions [39]), the posterior will converge to a Gaussian distribution centred around the true value **θ**_0_, with variance that shrinks to zero at the asymptotically optimal rate (given by the Cramér–Rao lower bound), i.e.

$$\pi (\theta |y_{1:n})\approx N\left(\theta_{0},\frac{1}{n}I{\left(\theta_{0}\right)}^{-1}\right),$$

where *y*_1:*n*_ = (*y*_1_, …, *y*_*n*_), and $I(\theta_{0})$ is the Fisher information matrix for the true parameter value **θ**_0_.

However, when our model is misspecified, i.e. g∉P (there is no **θ** ∈ **Θ** for which *g*( · ) = *f*( · |**θ**)), if we do inference for **θ** ignoring the discrepancy, then we usually still get asymptotic convergence of the maximum-likelihood estimator and Bayesian posterior [40,41]. However, instead of converging to a true value (which does not exist), we converge to the *pseudo-true* value

$$\theta^{*}={\text{arg min}}_{\theta \in \Theta }\text{KL}(g(\cdot )\;||\;p(\cdot |\theta )),$$

where KL(g||p)=∫g(x)log⁡(g(x)/p(x))dx is the Kullback–Leibler divergence from *p* to *g* (a measure of the difference between two distributions). In other words, we converge upon the model, $p(\cdot |\theta^{*})$, which is closest to the DGP as measured by the Kullback–Leibler divergence (figure 4).

**Figure 4.**
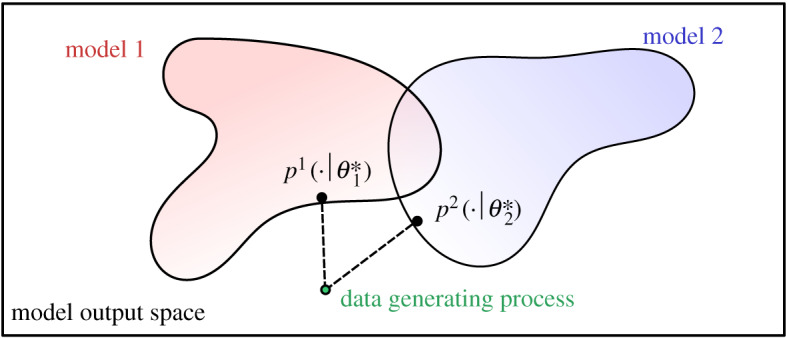
A cartoon to illustrate the effect of model discrepancy on parameter fits in different models. Each cloud represents a range of possible outputs from each model, which they can reach with different parameter values. The true data generating process (DGP) lies outside either of our imperfect model classes 1 and 2, and neither can fit the data perfectly due to model discrepancy. When we attempt to infer parameters, we will converge upon models that generate outputs closest to the true DGP under the constraint of being in each model. Adding more data just increases the confidence in being constrained to model parameterizations on the boundary of the particular model, i.e. we become certain about $\theta^{*}$, the pseudo-true parameter value for each model. Note that different models will have different pseudo-true parameter values. (Online version in colour.)

Perhaps more importantly from a UQ perspective, as well as getting a point estimate that converges to the wrong value, we still usually get asymptotic concentration at rate 1/*n*, i.e. the posterior variance shrinks to zero. That is, we have found model parameters that are wrong, and yet we are certain about this wrong value. The way to think about this is that the Bayesian approach is not quantifying our uncertainty about a meaningful physical parameter **θ**_0_, but instead it gives our uncertainty about the pseudo-true value $\theta^{*}$. Consequently, we can not expect our calibrated predictions

$$\pi (y^{\prime }|y)=\int p(y^{\prime }|\theta )\pi (\theta |y_{1:n})\text{d}\theta ,$$

to perform well, as we saw in the AP example above.

This leaves us with two options. We can either extend our model class P in the hope that we can find a class of models that incorporates the DGP (and which is still sufficiently simple that we can hope to learn the true model from the data), or we can change our inferential approach.

## 3. Accounting for model discrepancy

Once we have acknowledged that a model is misspecified, we are then faced with the challenge of how to handle the misspecification. The approach taken should depend upon the aim of the analysis. Using the model to predict independent events, for example a current time-series for some experimental protocol, will require a different approach if our aim is inference/calibration, i.e. if interest lies in the physical value of a particular parameter. In the first case (prediction), it can often suffice to fit the model to the data ignoring discrepancy, and then to correct the predictions in some way,^1^ although this may not work well if the prediction involves extrapolating into a regime far away from the data. The latter case (calibration) is more challenging, as we need to jointly fit the model and the discrepancy model, which can lead to problems of non-identifiability.

The most common approach for dealing with discrepancy is to try to correct the simulator by expanding the model class. The simplest approach is simply to add a flexible, non-parametric term to the simulator output, i.e.instead of assuming the data arose from equation (1.1), to assume

3.1 $$y=f(\theta ,u_{C})+\delta (v_{C})+\epsilon .$$

Here, *δ*(*v*_*C*_) is the model discrepancy term, and *ϵ* remains an unstructured white noise term. Note that *v*_*C*_ is used as the input to *δ* as it is not necessary to have the same input as the mechanistic model: *v*_*C*_ could include some or all of *u*_*C*_, but may also include information from internal model variables (see §3d). To train this model, one option is to first estimate **θ** assuming equation (1.1), and then to train *δ* to mop up any remaining structure in the residual. However, a better approach is to jointly estimate *δ* and *θ* in a Bayesian approach [42]. Unfortunately, as demonstrated below, this often fails as it creates a non-identifiability between **θ** and *δ* when *δ* is sufficiently flexible: for any **θ**, there exists a functional form *δ*( · ) for which equation (3.1) accurately represents the DGP. Brynjarsdóttir *et al.* [27] suggested that the solution is to strongly constrain the functional form of *δ*( · ) using prior knowledge. They present a toy situation in which *δ*(0) = 0 and *δ*(*x*) is monotone increasing, and show that once armed with this knowledge, the posterior *π*(**θ**| *y*) more accurately represents our uncertainty about **θ**. However, knowledge of this form is not available in many realistic problems.

### (a) Ion channel model example

We now illustrate the difficulty of accounting for model discrepancy in a tutorial example. We demonstrate that it can be hard to determine the appropriate information to include in *δ*, and that different functional forms for *δ* can lead to different parameter estimates.

We consider three structurally different models: Models A, B and C. We take Model C as the ground truth model in this particular example, and use it to perform synthetic voltage-clamp experiments and generate synthetic data. The goal is to use Models A and B to explain the generated synthetic data, assuming we have no knowledge about the ground truth Model C. This tutorial aims to demonstrate the importance of considering model discrepancy, jointly with model selection, to represent given data with unknown true DGP.

We use the hERG channel current as an example, and use three different model structures (shown in figure 5). Model A is a variant of the traditional Hodgkin–Huxley model, described in Beattie *et al.* [43]; Model B is used in Oehmen *et al.* [44]; and Model C is adapted from Di Veroli *et al.* [45].

**Figure 5.**
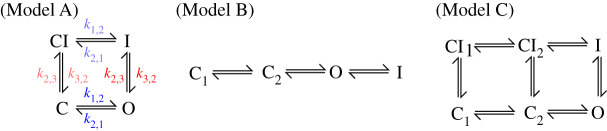
Markov model representation of Models A, B and C used in the ion channel model tutorial where Model C is taken as ground truth and used to generate synthetic data, while Models A and B are candidate models that we attempt to fit and use for predictions, demonstrating the challenge of both model discrepancy and model selection. (Online version in colour.)

All three ion channel models can be expressed using a Markov model representation. For a model with a state vector, **x** = (*x*_1_, *x*_2_, …)^*T*^, then in each case **x** evolves according to

3.2 $$\frac{\text{d}x}{\text{d}t}=Mx,$$

where **M** is the Markov matrix describing the transition rates between states. Markov models are linear coupled ordinary differential equations (ODEs) with respect to time, *t*, and states, **x**. Typically, the components in the Markov matrix, **M**, are nonlinear functions of voltage, *V*(*t*), which in these voltage-clamp experiments is an externally prescribed function of time known as the ‘voltage-clamp protocol’ (i.e. *u*_*C*_ in equation (1.1)). The observable, the macroscopic ionic current, *I*, measured under *V*(*t*), is

3.3 I(t,V)=g⋅O⋅(V−E),

where *g* is the maximum conductance, *E* is the reversal potential and O is the sum of all ‘open states’ in the model.

Take Model B as an example. Its state vector, **x**, and Markov matrix, **M**, can be written as

3.4 $$x=\left(\begin{array}{c} x_{1}\\ x_{2}\\ x_{3}\\ x_{4} \end{array}\right)=\left(\begin{array}{c} {\text{C}}_{2}\\ {\text{C}}_{1}\\ \text{O}\\ \text{I} \end{array}\right);\;\text{and}\;M=\left(\begin{array}{cccc} -k_{1,2} & k_{2,1} & 0 & 0\\ k_{1,2} & -k_{2,1}-k_{2,3} & k_{3,2} & 0\\ 0 & k_{2,3} & -k_{3,2}-k_{3,4} & k_{4,3}\\ 0 & 0 & k_{3,4} & -k_{4,3} \end{array}\right),$$

where *x*_*i*_ is the probability a gate is in state *i* (or equivalently, the proportion of gates which are in state *i*), with $\sum x_{i}=1$. The parameters *k*_*i*,*j*_ represent the transition rates from state *x*_*i*_ to state *x*_*j*_. Note that for all our models, there is just one open state so that O=O. For all three models, each transition rate, *k*_*i*,*j*_, is voltage dependent and takes the form

3.5 $$k_{i,j}(V)=A_{i,j}\exp(B_{i,j}V),$$

with two parameters (*A*_*i*,*j*_, *B*_*i*,*j*_) to be inferred. This yields a total of 12 parameters for Model B which we denote as {*p*_1_, …, *p*_12_}, together with the maximum conductance, *g*, to be found. Similarly for Model A, it has eight parameters {*p*_1_, …, *p*_8_} together with *g*, to be inferred.

### (b) Synthetic experiments

We let Model C be the ground truth DGP and simulate data from it (using parameter values estimated from real room temperature data by Beattie *et al.* [43], where *g* = 204 nS). We add i.i.d. Gaussian noise with zero mean and standard deviation *σ* = 25 pA to the simulated data. We generate data under three different voltage-clamp protocols, *V*(*t*). These are a sinusoidal protocol (see top plot in figure 6) and an AP series protocol from Beattie *et al.* [43] (see electronic supplementary material, figure S9), and the staircase protocol from Lei *et al.* [26,46] (figure 6*b*).

**Figure 6.**
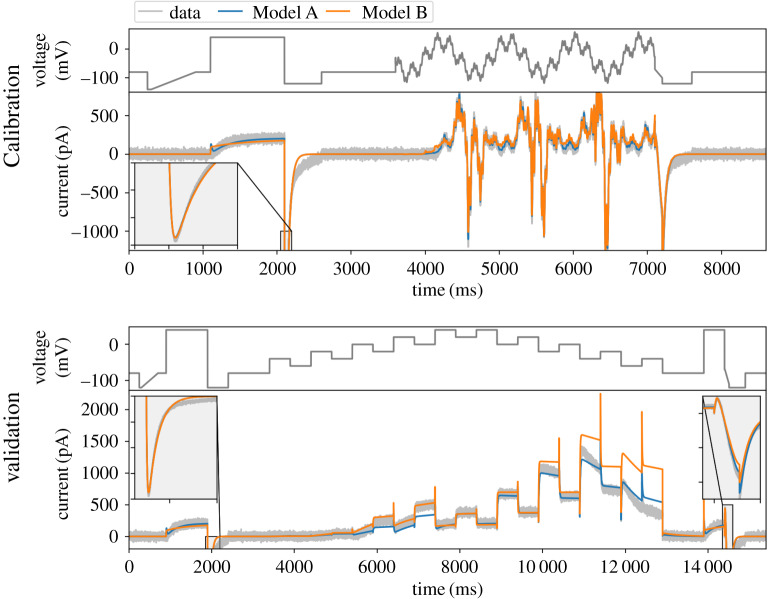
(*a*) The fitted model predictions for Models A (blue) and B (orange) for the ion channel example (i.e. model predictions for the data they were trained with). Both models have been fitted to synthetic calibration data (grey) generated using Model C using the sinusoidal voltage-clamp protocol [43]. (*b*) Models A (blue) and B (orange) predictions for the validation data (grey) generated from Model C under the staircase protocol [26] (not used in training). Note that there are significant discrepancies around 12 000 ms. (Online version in colour.)

### (c) Standard calibration ignoring model discrepancy

To calibrate the model (without considering any model discrepancy), we assume a statistical model of the form of equation (1.1), which has the same observation noise model as our synthetic data. The likelihood of model parameter **θ**, having observed the data **y** = *y*_1:*n*_, is given by equation (1.3).

We use the sinusoidal protocol (figure 6*a*) as the calibration protocol; the AP series protocol (electronic supplementary material, top, figure S9) and the staircase protocol (electronic supplementary material, figure S9) are used as validation data. We use a global optimization algorithm [35] to fit the model parameters using their maximum-likelihood estimates. All inference is done using PINTS [36].

The fitting results of Models A and B are shown in figure 6. Using different starting points in the optimization gives almost exactly the same parameter sets each time. Although both models fit the calibration data reasonably well, neither matches perfectly, due to model discrepancy. While the exact forms of the model discrepancy differs between the two models, both models notably fail to reproduce the correct form of the current decay following the step to −120 mVshortly after 2000 ms.

The validation predictions for the staircase protocol are also shown in figure 6. Unlike in the sinusoidal protocol, where Model A generally gives a better prediction than Model B, in the staircase protocol, it is more evident that the model discrepancy traits are different for each model. For example, Model B appears to give slightly better predictions of the current during the first 10 000 ms, whereas after this point Model A begins to give better predictions.

### (d) Calibration with model discrepancy

We now consider an approach that allows us to incorporate model discrepancy when doing parameter inference and making predictions. We adapt the method proposed in [42] and instead of assuming independent errors in equation (1.1), which corresponds to assuming a diagonal covariance matrix for the vector of errors **ϵ**, we consider an additive discrepancy model of the form given by equation (3.1), giving a correlated (non-diagonal) error structure. We consider three different choices for the discrepancy *δ*(*v*_*C*_), and jointly infer *θ* and *δ*. Note that we allow for a different choice of input *v*_*C*_, compared to the input of model *f*, *u*_*C*_.

First, we model *δ* as a sparse-GP [47,48], for which we adapted the implementation in PyMC3 [49] using Theano [50]. The radial basis function was used for the results presented here; we also tried two other GP covariance functions (the exponential covariance function and the Matérn 3/2 covariance function) in electronic supplementary material, §S7(c), where we found the impact of the choice of covariance functions in this problem is not as sensitive as the formulation of the discrepancy models. We explore two possibilities: choosing *v*_*C*_ to be either (i) *t* (time); or (ii) *O*,*V* (the open probability, O in equation (3.3), and the voltage, *V*). In fitting the model, we estimate hyperparameters associated with the GP covariance function, and condition the model on the observed discrepancies. For the GP(*O*, *V*) model, this means that we assume that the discrepancy is a function *O* and *V* so that we use the observed combinations of (*O*, *V*, *δ*) to predict future discrepancies; in the GP(*t*) model, it means that we assume the discrepancy process is always similarly distributed in time (which will not be a sensible assumption in most situations). Full details are provided in the electronic supplementary material, §S2.

As a third approach, we model discrepancy *δ* and the white noise error *ϵ*, as an autoregressive-moving-average (ARMA) model of order *p*, *q* [51]. If *e*_*t*_ = *δ*_*t*_(*v*_*c*_) + *ϵ*_*t*_ is the residual at time *t*, then an ARMA(*p*, *q*) model for *e*_*t*_ is

3.6 $$e_{t}=\nu_{t}+\sum_{t^{\prime }=1}^{p}\varphi_{t}e_{t-t^{\prime }}+\sum_{t^{\prime }=1}^{q}\zeta_{t^{\prime }}\nu_{t-t^{\prime }},$$

where $\nu_{t}∼N(0,\tau^{2})$, and φ_1_, …, φ_*p*_ and *ζ*_1_, …, *ζ*_*q*_ are, respectively, the coefficients of the autoregressive and moving-average part of the model. We used the StatsModels [52] implementation, and assumed *p* = *q* = 2 throughout. Note that when using the ARMA model, we do not condition on the observed discrepancy sequence (so the mean of the ARMA process remains zero, unlike in the GP approaches), but only use it to correlate the discrepancy structure in time. In general, there is an interesting connection between GPs discretely sampled regularly in time, and autoregressive processes [47], but here we treat the ARMA process differently to how we use GP discrepancies, and use the data only to estimate the ARMA parameters, not to condition the process upon the observed temporal structure, i.e. we use the ARMA process as a simple approach for introducing correlation into the residuals to better account for the discrepancy, not to correct the discrepancy (as is done with the GP). The motivation is that if the mechanistic model is correct, the residuals should be uncorrelated, but for misspecified models they will typically be correlated. For further details, please refer to electronic supplementary material, §S3.

For all methods, i.i.d. noise, GP(*t*), GP(*O*, *V*) and ARMA(2, 2), we infer the posterior distribution of the parameters (equation (1.2)), where the priors are specified in electronic supplementary material, §S4. We use an adaptive covariance MCMC method in PINTS [32,36] to sample from the posterior distributions. The trace plots of the samples are shown in electronic supplementary material, §S7. The inferred (marginal) posterior distributions for Model A are shown in figure 7, and they are used to generate the posterior predictive distributions shown in figure 8. Electronic supplementary material, figure S16 shows the same plots for Model B. Note that the choice of the discrepancy model can shift the posterior distribution significantly, both in terms of its location and spread. In particular, the ARMA(2, 2) model gives a much wider posterior than the other discrepancy models.

**Figure 7.**
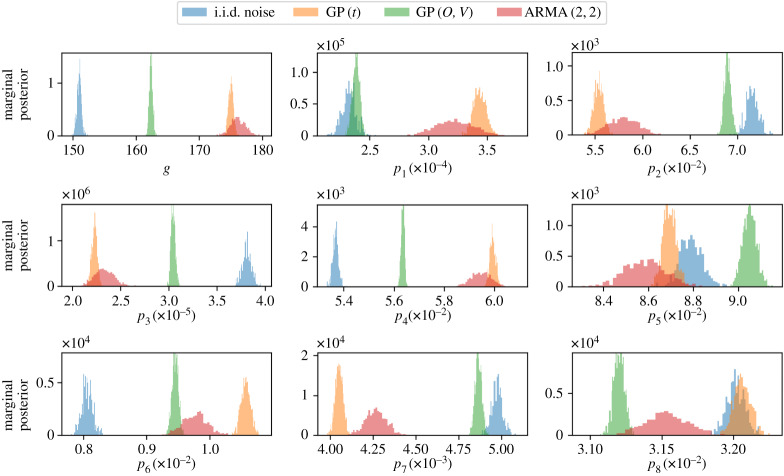
Model A inferred marginal posterior distributions for the conductance, *g* in equation (3.3), and kinetic parameters *p*_1_, …, *p*_8_ (a list of parameters referring to *A*_*i*,*j*_ and *B*_*i*,*j*_ in equation (3.5)) with different discrepancy models: i.i.d. noise (blue), GP(*t*) (orange), GP(*O*, *V*) (green) and ARMA(2, 2) (red). (Online version in colour.)

**Figure 8.**
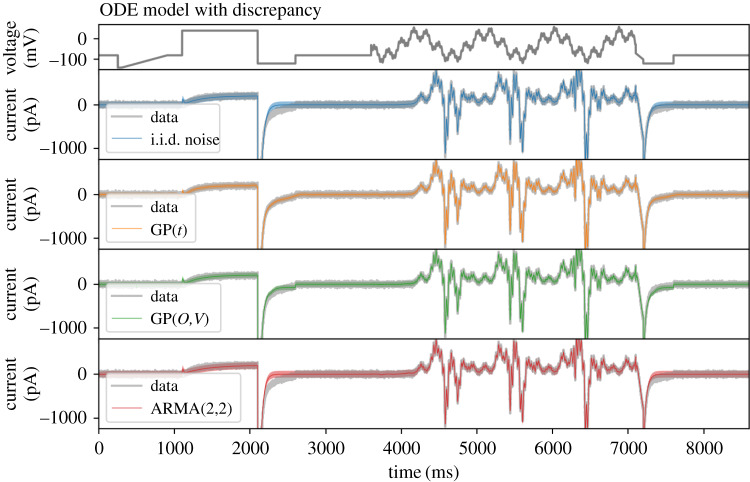
Model A fitted to the sinusoidal calibration protocol using the different discrepancy models: i.i.d. noise, GP(*t*), GP(*O*, *V*) and ARMA(2, 2). The plots show the mean (solid lines) and 95% credible intervals (shaded) of the posterior prediction for each model. (Online version in colour.)

Figure 8 shows the posterior predictive distributions of Model A with the calibration protocol using the four discrepancy models (electronic supplementary material, figure S17 for Model B), i.e.predicting the data used in training. The top panel shows the sinusoidal voltage protocol, and the panels underneath are calibrated model predictions with i.i.d. noise (blue), GP(*t*) (orange), GP(*O*, *V*) (green) and ARMA(2, 2) (red). The calibration data are shown in grey. Visually, we can see that the two GP models, GP(*t*) (orange) and GP(*O*, *V*) (green), fit the data with high accuracy; later we will see one of them is overfitting, while the other is not. The ARMA(2, 2) model (red) increases the width of the posterior (compared to i.i.d. noise), but its posterior mean prediction does not follow the data as closely as the two GP models.

Table 2 shows the root mean square errors (RMSEs) of the posterior mean predictions for all of the models, and is coloured so that yellow highlights the best performing model and red the worst. The first row of the table shows the results for the calibration (sine wave) protocol, and it is clear that the GP(*t*) and GP(*O*, *V*) models give the best RMSE values for the calibration data. Note that the RMSE only assesses the accuracy of the point estimate (given by the posterior mean). Table S1 in the electronic supplementary material gives the posterior predictive log-likelihoods; the log-likelihood is a proper scoring rule [53] which assesses the entire predictive distribution, not just the mean prediction. The ARMA(2, 2) and GP(*O*, *V*) models achieve the highest log-likelihood scores on the calibration data (best all round predictions when accounting for uncertainty).

**Table 2.** Models A (top) and B (bottom) RMSEs with different discrepancy models: i.i.d. noise, GP(*t*), GP(*O*, *V*) and ARMA(2, 2) for each of the three voltage protocols. Here, ‘ODE model-only’ refers to the predictions using only the calibrated ODE model under different discrepancy models (i.e.the model is calibrated assuming equation (3.1), but prediction is done using only $f(\hat{\theta },u_{C})$). See also electronic supplementary material, figures S13–S15 for Model A and figures S23–S25 for Model B.

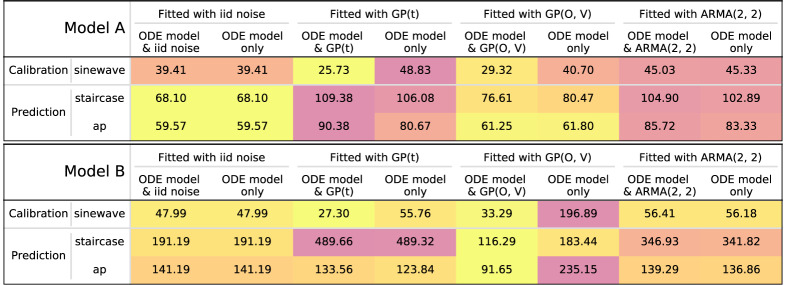

Figure 9 shows the prediction results for the staircase validation protocol for Model A (electronic supplementary material, figure S18 for Model B) using different discrepancy models, with the same layout as figure 8. Similar figures for the AP protocol predictions are shown in electronic supplementary material, figures S9 (Model A) and S19 (Model B). The GP(*t*) discrepancy model is conditioned to give the same temporal discrepancy pattern as in the calibration protocal, and is unable to change its predicted discrepancy in any way for the validation protocol; i.e. the GP(*t*) discrepancy predicts as if it were still under the sinusoidal protocol. Thus, there is some residual from the calibration protocol shown in the GP(*t*) (orange) prediction for the staircase protocol, e.g. see ‘wobbly’ current at approxiametely 7000 ms as pointed at by the blue arrow.

**Figure 9.**
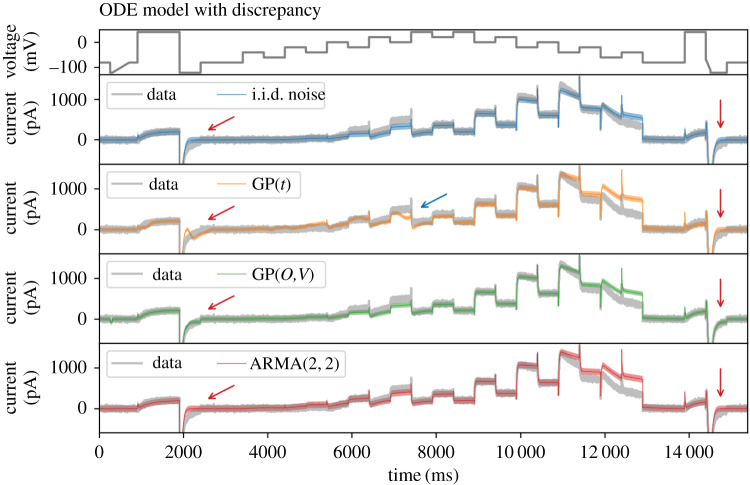
Model A’s prediction using the discrepancy models (i.i.d. noise, GP(*t*), GP(*O*, *V*) and ARMA(2, 2)), trained using the staircase voltage-clamp protocol [26]. We plot the posterior predictive mean (solid lines) with 95% credible intervals (shaded). The red arrows point to the tail current after the two activation steps, and mark an area of visible model mismatch: note the different performance of the four discrepancy models in this region. The blue arrow points to an obvious artefact at approximately 7000 ms induced by the GP(*t*) prediction which was trained on the sinusoidal protocol, and which does not take into account that we are now predicting for the staircase protocol. (Online version in colour.)

For Model A, it is interesting to see that the RMSE of the point prediction (the posterior mean) in table 2 (top) is best for the i.i.d. noise model with the GP(*O*, *V*) model only a little worse. Note that the GP(*O*, *V*) model is able to capture and accurately predict the tail current after the two activation steps, as indicated by the red arrows in figure 9—a visible area of model mismatch in our calibration without model discrepancy. The uncertainty quantification in the predictions is poor for all of the discrepancy models, but from electronic supplementary material, table S1 we can see that when we assess the uncertainty in the prediction, the i.i.d. noise model is the worst performing model (as for intervals where the prediction is wrong, each error is equally surprising, whereas in correlated models, the first error in any interval makes subsequent errors more probable). The unstructured ARMA(2, 2) and GP(*O*, *V*) models score highest for their uncertainty quantification.

For Model B, the GP(*O*, *V*) discrepancy model gives the best overall predictions for both the staircase and the AP protocols, although when we examine the contributions of the mechanistic and discrepancy models, we see that an element of non-identifiability between them has arisen (electronic supplementary material, §S7b). In terms of the posterior predictive log-likelihood, electronic supplementary material table S1 (bottom) again highlights that the ARMA(2, 2) and GP(*O*, *V*) models tend to be better than the i.i.d. noise and GP(*t*) models.

Electronic supplementary material, figures S10, S11 and S12 show the model discrepancy for Model A for the sine wave protocol, AP protocol and staircase protocol, respectively; electronic supplementary material, figures S20, S21 and S22 show the same plots for Model B. Electronic supplementary material, figures S21 and S22, in particular, highlight that the GP(*t*) model has, by design, learnt nothing of relevance about model discrepancy for extrapolation under an independent validation protocol (in which *V*(*t*), and indeed the range of *t* differs from that of the training protocol). Furthermore, the discrepancy model is based only on information extending to 8000 ms (the duration of the training protocol), after which the credible interval resorts to the width of the GP prior variance. By contrast, the GP(*O*, *V*) model learns, independently of *t*, the discrepancy under combinations of (*O*, *V*) present in the training data (such as the activation step to 40 mV followed by a step to −120 mV), which is why it is able to better predict the tail current after the two activation steps. Finally, the ARMA(2, 2) model has zero mean with similar 95% credible intervals to the i.i.d. noise model, but has correlated errors and so scores better in terms of the posterior predictive log-likelihood. The ion channel (ODE) model-only predictions for the sine wave protocol, AP protocol and staircase protocol are shown in electronic supplementary material, figures S13, S14 and S15 for Model A and figures S23, S24 and S25 for Model B.

For a given dataset, the RMSE and log-likelihood values in table 2 and electronic supplementary material table S1 are comparable across models. Note that Model A is more accurate than Model B on all datasets and with all discrepancy models. With Model A, none of the discrepancy models are able to improve the mean predictions over the i.i.d. noise model performance, but the GP(*O*, *V*) comes close (in RMSE) while being able to capture some of the nonlinear dynamics that Model A misses, as discussed above. With Model B, the GP(*O*, *V*) model gives the best mean predictions (as measured by the RMSE). The GP(*t*) model achieves a better score on the calibration data, but by over-fitting the data. The ARMA(2, 2) model consistently gives the best posterior predictive log-likelihood values for Models A and B, as it gives a wider posterior distribution compared to other methods (figure 7). Over-confident predictions are heavily penalized by the log-likelihood, which explains the large differences observed in these values.

To conclude, we have used two different incorrect model structures (Models A, B) to fit synthetic data generated from a third model (Model C). We considered both ignoring and incorporating discrepancy when calibrating the model. Calibrating with discrepancy improved predictions notably for Model B, but not for Model A. Although our problem was a time-dependent (ODE) system, constructing the discrepancy model as a pure time-series based function is not necessarily useful in predicting unseen situations; we found the GP(*O*, *V*) model performed best at correcting the point prediction from the models.

## 4. Discussion

In this review and perspective piece, we have drawn attention to an important and under-appreciated source of uncertainty in mechanistic models—that of uncertainty in the model structure or the equations themselves (model discrepancy). Focusing on cardiac electrophysiology models, we provided two examples of the consequences of ignoring discrepancy when calibrating models at the ion channel and AP scales, highlighting how this could lead to over-confident parameter posterior distributions and subsequently spurious predictions.

Statistically, we can explicitly admit discrepancy exists, and include it in the model calibration process and predictions. We attempted to do this by modelling discrepancy using two proposals from the literature—GPs trained on different inputs and an autoregressive-moving-average (ARMA) model. We saw how GPs can achieve some success in describing discrepancy in the calibration experiment. A two-dimensional GP in voltage and time was used previously by Plumlee *et al.* [20,21], where it was used to extrapolate to new voltages for a given single step voltage-clamp experiment. To use a discrepancy model to make predictions for unseen situations, it needs to be a function of something other than time, otherwise features specific to the calibration experiment are projected into new situations. A promising discrepancy model was our two-dimensional GP as a function of the mechanistic model’s open probability and voltage, although for Model B this led to ambiguity between the role of the ODE system and the role of the discrepancy (see electronic supplementary material §S7b).

The modelling community would hope to study any discrepancy model that is shown to improve predictions, and use insights from this process to iteratively improve the mechanistic model. How we handle model discrepancy may depend on whether we are more interested in learning about what is missing in the model, or in making more reliable predictions: both related topics are worthy of more investigation.

### (a) Recommendations

Very rarely do computational studies use more than one model to test the robustness of their predictions to the model form. We should bear in mind that all models are approximations and so when we are comparing to real data, all models have discrepancy. Here, we have seen, using synthetic data from an assumed true data-generating model, how fragile the calibration process can be for models with discrepancy, and how this discrepancy manifests itself in predictions of unseen situations. Synthetic data studies, simulating data from different parameter sets and different model structures, allow the modeller to test how well the inverse problem can be solved and how robust predictions from the resulting models are [54]. We strongly recommend performing such studies to learn more about the chosen, and alternative, models, as well as the effects of the model choice on parameter calibration and subsequent predictions. To develop our field further, it will be important to document the model-fitting process, and to make datasets and infrastructure available to perform and reproduce these fits with different models [55].

### (b) Open questions and future work

The apparent similarity of the AP models we looked at (summarized in figure 1) is a challenge for model calibration. A number of papers have emphasized that more information can be gained to improve parameter identifiability with careful choice of experimental measurements, in particular by using membrane resistance [30,34], or other protocols promoting more information-rich dynamics [31,32] and some of these measurements may be more robust to discrepancy than others.

In synthetic data, fitting the model used to generate the data will recover the same parameter set from any different protocol (where there is sufficient information to identify the parameters). But in the presence of discrepancy, fitting the same model to data from different protocols/experiments will result in different parameter sets, as the models make the best possible compromise (as shown schematically in figure 4). This phenomenon may be an interesting way to approach and quantify model discrepancy.

If the difference between imperfect model predictions represented the difference between models and reality then this may also provide a way to estimate discrepancy. For instance, the largest difference between the ion channel Model A and B predictions in the staircase protocol was at the point in time that both of them showed largest discrepancy (figure 6). Some form of Bayesian model averaging [56], using variance-between-models to represent discrepancy, may be instructive if the models are close enough to each other and reality, but can be misleading if the ensemble of models is not statistically exchangeable with the DGP [57,58] or if there is some systematic error (bias) due to experimental artefacts [59].

In time-structured problems, rather than adding a discrepancy to the final simulated trajectory, as we have done here, we can instead change the dynamics of the model directly. It may be easier to add a discrepancy term to the differential equations to address misspecification, than it is to correct their solution, but the downside is that this makes inference of the discrepancy computationally challenging. One such approach is to convert the ODE to a stochastic differential equation [60,61], i.e. replace $dx/dt=f_{\theta }(x,t)$ by $dx=f_{\theta }(x,t)dt+\Sigma^{1/2}dW_{t}$ where *W*_*t*_ is a Brownian motion with covariance matrix Σ. This turns the deterministic ODE into a stochastic model and can improve the UQ, but cannot capture any structure missing from the dynamics. We can go further and attempt to modify the underlying model equations, by changing the ODE system to

4.1 $$\frac{dx}{dt}=f_{\theta }(x,t)+\delta (x),$$

where again *δ*(**x**) is an empirical term to be learnt from the data. For example, this has been tried with a discretized version of the equations using a parametric model for *δ* [62], with GPs [63], nonlinear autoregressive exogenous (NARX) models [64] and deep neural networks [65]. Computation of posterior distributions for these models is generally challenging, but is being made easier by the development of automatic-differentiation software, which allows derivative information to be used in MCMC samplers, or in variational approaches to inference (e.g. [66,67]).

Ultimately, modelling our way out of trouble, by expanding the model class, may prove impossible given the quantity of data available in many cases. Instead, we may want to modify our inferential approach to allow the best judgements possible about the parameters given the limitation of the model and data. Approaches such as approximate Bayesian computation (ABC) [68] and history-matching [69,70] change the focus from learning a statistical model within a Bayesian setting, to instead only requiring that the simulation gets within a certain distance of the data. This change, from a fully specified statistical model for *δ* to instead only giving an upper bound for *δ*, is a conservative inferential approach where the aim is not to find the best parameter values, but instead rule out only obviously implausible values [71,72].

For example, in the AP model from §2, instead of taking a Bayesian approach with an i.i.d. Gaussian noise model, we can instead merely try to find parameter values that get us within some distance of the calibration data (for details, see electronic supplementary material, figure S2). In the electronic supplementary material, we describe a simple approach, based on the methods presented in [73], where we find 1079 candidate parameter sets that give a reasonable match to the calibration data. When we use these parameters to predict the 2 Hz validation data, and the 75% *I*_Kr_ block CoU data, we get a wide range of predictions that incorporate the truth (electronic supplementary material, figure S3)—for a small subset of 70 out of 1079, we get good predictions and not the catastrophic prediction shown in figure 2. By acknowledging the existence of model discrepancy, the use of wider error bounds (or higher-temperature likelihood functions) during the fitting process may avoid fitting parameters overly-precisely. However, we have no way of knowing which subset of remaining parameter space is more plausible (if any) without doing these further experiments; testing the model as close as possible to the desired context of use helps us spot such spurious behaviour.

This paper has focused on the ion channel and AP models of cardiac electrophysiology. There is an audit of where uncertainty appears in cardiac modelling and simulation in this issue [74]. The audit highlights many other areas where discrepancy may occur: in assumptions homogenizing the subcellular scale to the models we have here; or at the tissue and organ scales in terms of spatial heterogeneity, cell coupling or mechanical models for tissue contraction and fluid-solid interaction. All of these areas need attention if we are to prevent model discrepancy producing misleading scientific conclusions or clinical predictions.

## 5. Conclusion

In this paper, we have seen how having an imperfect representation of a system in a mathematical model (discrepancy) can lead to spuriously certain parameter inference and overly-confident and wrong predictions. We have examined a range of methods that attempt to account for discrepancy in the fitting process using synthetic data studies. In some cases, we can improve predictions using these methods, but different methods work better for different models in different situations, and, in some cases, the best predictions were still made by ignoring discrepancy. A large benefit of the calibration methods which include discrepancy is that they better represent uncertainty in predictions, although all the methods we trialled still failed to allow for a wide enough range of possible outputs in certain parts of the protocols. Methodological developments are needed to design reliable methods to deal with model discrepancy for use in safety-critical electrophysiology predictions.

## Supplementary Material

Supplementary Methods and Results

## Footnotes

1 Note, however, that jointly fitting model and discrepancy can make the problem easier, for example by making the discrepancy a better behaved function more amenable to being modelled.

## Data accessibility

Code to reproduce the results in the tutorials is available at https://github.com/CardiacModelling/fickleheart-method-tutorials.
