# Supplementary material for: Considering discrepancy when calibrating a mechanistic electrophysiology model
Source: Philos Trans A Math Phys Eng Sci. 2020 May 25;378(2173):20190349. doi: 10.1098/rsta.2019.0349 (PMC7287333; doi:10.1098/rsta.2019.0349)
Supplement: Supplementary Methods and Results [file rsta20190349supp1.pdf]

PHILOSOPHICAL TRANSACTIONS A

rsta.royalsocietypublishing.org

Article Supplementary Material

Considering discrepancy when calibrating a mechanistic electrophysiology model: Supplementary Material

Chon Lok Lei, Sanmitra Ghosh, Dominic G. Whittaker, Yasser Aboelkassem, Kylie A. Beattie, Chris Cantwell, Tammo Delhaas, Charles Houston, Gustavo Montes Novaes, Alexander V. Panfilov, Pras Pathmanathan, Marina Riabiz, Rodrigo Weber dos Santos, John Walmsley, Keith Worden, Gary R. Mirams and Richard D. Wilkinson

Supplementary Material

Contents

S1 Differences between Model T and Model F 2

S2 Modelling discrepancy using a Gaussian process 6

S3 Modelling residuals using an ARMA(p, q) process 9

S4 Choice of priors for the ion channel example 11

S5 Computing and representing posterior predictive 12

S6 Supplementary results for the action potential example 13

S7 Supplementary results for the ion channel discrepancy example 16

- (a) Model A 17
  - i Model A: Full model predictions . 19
  - ii Model A: Discrepancy predictions 20
  - iii Model A: ODE model predictions . 23
- (b) Model B 26
  - i Model B: Full model predictions . . 27
  - ii Model B: Discrepancy predictions 30
  - iii Model B: ODE model predictions . 33
- (c) GP covariance functions: RBF, OU and Matern3/2 36

References 45

© The Authors. Published by the Royal Society under the terms of the Creative Commons Attribution License http://creativecommons.org/licenses/by/4.0/, which permits unrestricted use, provided the original author and source are credited.

THE ROYAL SOCIETY
PUBLISHING

S1. Differences between Model T and Model F

Here we show the equations of the currents in Model T [1] and Model F [2] that are different in kinetics. Below g_X is the conductance (constant), E_X is the reversal potential, X_o is the extracellular concentration, X_i is the intracellular concentration of ion X. V is the membrane voltage, F is Faraday constant, R is the ideal gas constant.

I_{CaL}

Model T

$$I_{CaL} = \frac{g_{CaL} \cdot d \cdot f \cdot fCa \cdot 4 \cdot V \cdot F^2}{R \cdot 310} \times \frac{\left(Ca_i \cdot e^{\frac{2 \cdot V \cdot F}{R \cdot 310}} - 0.341 \cdot Ca_o\right)}{\left(e^{\frac{2 \cdot V \cdot F}{R \cdot 310}} - 1\right)}$$

$$d_\infty = \frac{1}{\left(1 + e^{\frac{(-5-V)}{7.5}}\right)}$$

$$\alpha_d = \left(\frac{1.4}{\left(1 + e^{\frac{(-35-V)}{13}}\right)} + 0.25\right)$$

$$\beta_d = \frac{1.4}{\left(1 + e^{\frac{(V+5)}{5}}\right)}$$

$$\gamma_d = \frac{1}{\left(1 + e^{\frac{(50-V)}{20}}\right)}$$

$$\tau_d = (\alpha_d \cdot \beta_d + \gamma_d)$$

$$\frac{\mathrm{d}d}{\mathrm{d}t} = \frac{(d_\infty - d)}{\tau_d}$$

$$f_\infty = \frac{1}{\left(1 + e^{\frac{(V+20)}{7}}\right)}$$

$$\tau_f = \left(1125 \cdot e^{\frac{-(V+27)^2}{240}} + 80 + \frac{165}{\left(1 + e^{\frac{(25-V)}{10}}\right)}\right)$$

$$\frac{\mathrm{d}f}{\mathrm{d}t} = \frac{(f_\infty - f)}{\tau_f}$$

$$\alpha_{fCa} = \frac{1}{\left(1 + \left(\frac{Ca_i}{0.000325}\right)^8\right)}$$

$$\beta_{fCa} = \frac{0.1}{\left(1 + e^{\frac{(Ca_i - 0.0005)}{0.0001}}\right)}$$

$$\gamma_{fCa} = \frac{0.2}{\left(1 + e^{\frac{(Ca_i - 0.00075)}{0.0008}}\right)}$$

$$fCa_\infty = \frac{(\alpha_{fCa} + \beta_{fCa} + \gamma_{fCa} + 0.23)}{1.46}$$

$$\tau_{fCa} = 2$$

$$d_{fCa} = \frac{(fCa_\infty - fCa)}{\tau_{fCa}}$$

$$\frac{\mathrm{d}fCa}{\mathrm{d}t} = \begin{cases} 0; & \text{if } (fCa_\infty > fCa) \text{ and } (V > -60), \\ d_{fCa} & \text{otherwise.} \end{cases}$$

Model F

$$I_{CaL} = \frac{g_{CaL} \cdot d \cdot f \cdot f_2 \cdot fCass \cdot 4 \cdot (V - 15) \cdot F^2}{R \cdot 310} \times \frac{\left(0.25 \cdot Ca_{ss} \cdot e^{\frac{2 \cdot (V-15) \cdot F}{R \cdot 310}} - Ca_o\right)}{\left(e^{\frac{2 \cdot (V-15) \cdot F}{R \cdot 310}} - 1\right)}$$

$$d_\infty = \frac{1}{\left(1 + e^{\frac{(5-V)}{7.5}}\right)}$$

α_d = as per Model T,

β_d = as per Model T,

γ_d = as per Model T,

τ_d = as per Model T,

$\frac{\mathrm{d}d}{\mathrm{d}t}$ = as per Model T,

f_∞ = as per Model T,

$$\tau_f = \frac{1}{4}\left(1102.5 \cdot e^{\frac{-(((V+27))^2)}{225}} + \frac{200}{\left(1 + e^{\frac{(13-V)}{10}}\right)} + \frac{180}{\left(1 + e^{\frac{(V+30)}{10}}\right)} + 20\right)$$

$\frac{\mathrm{d}f}{\mathrm{d}t}$ = as per Model T,

$$f2_\infty = \left(\frac{0.75}{\left(1 + e^{\frac{(V+35)}{7}}\right)} + 0.25\right)$$

$$\tau_{f2} = \frac{1}{2}\left(562 \cdot e^{\frac{-(V+27)^2}{240}} + \frac{31}{\left(1 + e^{\frac{(25-V)}{10}}\right)} + \frac{80}{\left(1 + e^{\frac{(V+30)}{10}}\right)}\right)$$

$$\frac{\mathrm{d}f_2}{\mathrm{d}t} = \frac{(f2_\infty - f_2)}{\tau_{f2}}$$

$$fCass_\infty = \left(\frac{0.4}{\left(1 + \left(\frac{Ca_{ss}}{0.05}\right)^2\right)} + 0.6\right)$$

$$\tau_{fCass} = \left(\frac{80}{\left(1 + \left(\frac{Ca_{ss}}{0.05}\right)^2\right)} + 2\right)$$

$$\frac{\mathrm{d}fCass}{\mathrm{d}t} = \frac{(fCass_\infty - fCass)}{\tau_{fCass}}$$

I_{to}

Model T

$$I_{to} = g_{to} \cdot r \cdot s \cdot (V - E_K)$$

$$s_\infty = \frac{1}{\left(1 + e^{\frac{(V+28)}{5}}\right)}$$

$$\tau_s = 1000 \cdot e^{\left(\frac{-(V+67)^2}{1000}\right)} + 8$$

$$\frac{\mathrm{d}s}{\mathrm{d}t} = \frac{(s_\infty - s)}{\tau_s}$$

$$r_\infty = \frac{1}{\left(1 + e^{\frac{(20-V)}{6}}\right)}$$

$$\tau_r = \left(9.5 \cdot e^{\frac{-(V+40)^2}{1800}} + 0.8\right)$$

$$\frac{\mathrm{d}r}{\mathrm{d}t} = \frac{(r_\infty - r)}{\tau_r}$$

Model F

$I_{to} =$ as per Model T,

$$s_\infty = \frac{1}{\left(1 + e^{\frac{(V+20)}{5}}\right)}$$

$$\tau_s = \left(85 \cdot e^{\frac{-(V+45)^2}{320}} + \frac{5}{\left(1 + e^{\frac{(V-20)}{5}}\right)} + 3\right)$$

$\frac{\mathrm{d}s}{\mathrm{d}t} =$ as per Model T,

r gate as per Model T.

I_{Kr}

Model T

$$I_{Kr} = g_{Kr} \cdot \sqrt{\frac{K_o}{5.4}} \cdot X_{r1} \cdot X_{r2} \cdot (V - E_K)$$

$$xr1_{\infty} = \frac{1}{\left(1 + e^{\frac{(-26-V)}{7}}\right)}$$

$$\alpha_{xr1} = \frac{450}{\left(1 + e^{\frac{(-45-V)}{10}}\right)}$$

$$\beta_{xr1} = \frac{6}{\left(1 + e^{\frac{(V+30)}{11.5}}\right)}$$

$$\tau_{xr1} = \alpha_{xr1} \cdot \beta_{xr1}$$

$$\frac{\mathrm{d}X_{r1}}{\mathrm{d}t} = \frac{(xr1_{\infty} - X_{r1})}{\tau_{xr1}}$$

$$xr2_{\infty} = \frac{1}{\left(1 + e^{\frac{(V+88)}{24}}\right)}$$

$$\alpha_{xr2} = \frac{3}{\left(1 + e^{\frac{(-60-V)}{20}}\right)}$$

$$\beta_{xr2} = \frac{1.12}{\left(1 + e^{\frac{(V-60)}{20}}\right)}$$

$$\tau_{xr2} = \alpha_{xr2} \cdot \beta_{xr2}$$

$$\frac{\mathrm{d}X_{r2}}{\mathrm{d}t} = \frac{(xr2_{\infty} - X_{r2})}{\tau_{xr2}}$$

Model F

$$I_{Kr} = g_{Kr} \cdot \left(\frac{310}{35} - \frac{55}{7}\right) \cdot \sqrt{\frac{K_o}{5.4}} \cdot Or4 \cdot (V - E_K)$$

$$\alpha_{xr1} = e^{(24.335+(0.0112 \cdot V-25.914))}$$

$$\beta_{xr1} = e^{(13.688+(-(0.0603) \cdot V-15.707))}$$

$$\alpha_{xr2} = e^{(22.746+(0 \cdot V-25.914))}$$

$$\beta_{xr2} = e^{(13.193+(0 \cdot V-15.707))}$$

$$\alpha_{xr3} = e^{(22.098+(0.0365 \cdot V-25.914))}$$

$$\beta_{xr3} = e^{(7.313+(-(0.0399) \cdot V-15.707))}$$

$$\alpha_{xr4} = e^{(30.016+(0.0223 \cdot V-30.888))} \cdot \left(\frac{5.4}{K_o}\right)^{0.4}$$

$$\beta_{xr4} = e^{(30.061+(-(0.0312) \cdot V-33.243))}$$

$$\frac{\mathrm{d}Cr1}{\mathrm{d}t} = (\beta_{xr1} \cdot Cr2 - \alpha_{xr1} \cdot Cr1)$$

$$\frac{\mathrm{d}Cr2}{\mathrm{d}t} = ((\alpha_{xr1} \cdot Cr1 + \beta_{xr2} \cdot Cr3) - (\alpha_{xr2} + \beta_{xr1}) \cdot Cr2)$$

$$\frac{\mathrm{d}Cr3}{\mathrm{d}t} = ((\alpha_{xr2} \cdot Cr2 + \beta_{xr3} \cdot Or4) - (\alpha_{xr3} + \beta_{xr2}) \cdot Cr3)$$

$$\frac{\mathrm{d}Or4}{\mathrm{d}t} = ((\alpha_{xr3} \cdot Cr3 + \beta_{xr4} \cdot Ir5) - (\alpha_{xr4} + \beta_{xr3}) \cdot Or4)$$

$$\frac{\mathrm{d}Ir5}{\mathrm{d}t} = (\alpha_{xr4} \cdot Or4 - \beta_{xr4} \cdot Ir5)$$

I_{K1}

Model T

$$I_{K1} = g_{K1} \cdot xK1_{\infty} \cdot \sqrt{\frac{K_o}{5.4}} \cdot (V - E_K)$$

$$\alpha_{K1} = \frac{0.1}{\left(1 + e^{0.06 \cdot ((V - E_K) - 200)}\right)}$$

$$\beta^a_{K1} = 3 \cdot e^{0.0002 \cdot ((V - E_K) + 100)}$$

$$\beta^b_{K1} = e^{0.1 \cdot ((V - E_K) - 10)}$$

$$\beta_{K1} = \frac{\beta^a_{K1} + \beta^b_{K1}}{\left(1 + e^{-(0.5) \cdot (V - E_K)}\right)}$$

$$xK1_{\infty} = \frac{\alpha_{K1}}{(\alpha_{K1} + \beta_{K1})}$$

Model F

$$I_{K1} = g_{K1} \cdot \left(\frac{310}{35} - \frac{55}{7}\right) \cdot \sqrt{\frac{K_o}{5.4}} \cdot xK1_{\infty} \cdot (V - E_K)$$

$$Ki_{Mg} = 2.8 \cdot e^{\frac{-(V - \delta \cdot E_K)}{180}}$$

$$Kb_{Mg} = 0.45 \cdot e^{\frac{-(V - \delta \cdot E_K)}{20}}$$

$$Kd1_{SPM} = 0.7 - 3 \cdot e^{\frac{-((V - \delta \cdot E_K) + 8 \cdot Mg_{Buf})}{4.8}}$$

$$Kd2_{SPM} = 40 - 3 \cdot e^{\frac{-(V - \delta \cdot E_K)}{9.1}}$$

$$X = \left(1 + \frac{Mg_{Buf}}{Kb_{Mg}}\right)$$

$$rec1 = \frac{X^2}{\left(\frac{SPM}{Kd1_{SPM}} + \frac{Mg_{Buf}}{Ki_{Mg}} + X^3\right)}$$

$$rec2 = \frac{1}{\left(1 + \frac{SPM}{Kd2_{SPM}}\right)}$$

$$xK1_{\infty} = (\phi \cdot rec1 + (1 - \phi) \cdot rec2)$$

$$Mg_{Buf} = 0.0356$$

$$SPM = 0.0014613$$

$$\phi = 0.8838$$

$$\delta = 1.0648$$

I_{Ks}

Model T

$$I_{Ks} = g_{Ks} \cdot X_s^2 \cdot (V - E_{Ks})$$

$$xs_{\infty} = \frac{1}{\left(1 + e^{\frac{(-5 - V)}{14}}\right)}$$

$$\alpha_{xs} = \frac{1100}{\sqrt{\left(1 + e^{\frac{(-10 - V)}{6}}\right)}}$$

$$\beta_{xs} = \frac{1}{\left(1 + e^{\frac{(V - 60)}{20}}\right)}$$

$$\tau_{xs} = \alpha_{xs} \cdot \beta_{xs}$$

$$\frac{\mathrm{d}X_s}{\mathrm{d}t} = \frac{(xs_{\infty} - X_s)}{\tau_{xs}}$$

Model F

$$I_{Ks} = \text{as per Model T,}$$

$$xs_{\infty} = \text{as per Model T,}$$

$$\alpha_{xs} = \frac{1400}{\sqrt{\left(1 + e^{\frac{(5 - V)}{6}}\right)}}$$

$$\beta_{xs} = \frac{1}{\left(1 + e^{\frac{(V - 35)}{15}}\right)}$$

$$\tau_{xs} = (\alpha_{xs} \cdot \beta_{xs} + 80)$$

$$\frac{\mathrm{d}X_s}{\mathrm{d}t} = \text{as per Model T.}$$

S2. Modelling discrepancy using a Gaussian process

In order to add a discrepancy term to our basic measurement model (see main text), we model the ith observation as:

$$(Y_C)_i = f_i(\boldsymbol{\theta}, u_C^i) + \delta_i(\boldsymbol{\phi}, \boldsymbol{v}_C^i) + \epsilon_i, \tag{S2.1}$$

where $\delta_i(\boldsymbol{\phi}, \boldsymbol{v}_C^i)$ is the model discrepancy term, a function with arguments $\boldsymbol{v}_C$ and parameters $\boldsymbol{\phi}$. Note that the inputs $\boldsymbol{v}_C$ can be independent from the inputs passed to the mechanistic model. We choose v_C to be (1) time t, and (2) the open probability O (i.e. $\mathcal{O}$ in Eq. (3.3)) and the voltage V.

Following [3] we place a zero mean Gaussian process prior on the discrepancy function given by

$$\delta(\boldsymbol{\phi}, \boldsymbol{v}_C) \sim \mathcal{GP}\big(0, \boldsymbol{\kappa}(\boldsymbol{v}_C, \boldsymbol{v}'_C; \boldsymbol{\phi})\big), \tag{S2.2}$$

where $\boldsymbol{\kappa}(\boldsymbol{v}_C, \boldsymbol{v}'_C; \boldsymbol{\phi})$ is the covariance function (also known as covariance *kernel*) parameterised by $\boldsymbol{\phi}$. One common choice for the covariance function is the squared exponential function given by

$$\boldsymbol{\kappa}(\boldsymbol{v}_C, \boldsymbol{v}'_C; \boldsymbol{\phi}) = \alpha^2 \exp\left(-\sum_{j=1}^{q} \frac{(v_{C_j} - v'_{C_j})^2}{2\rho_j^2}\right), \tag{S2.3}$$

where q is the number of covariates, such as time or open probability as mentioned above, representing $\boldsymbol{v}_C$. The parameter ρ_j quantifies the characteristic length-scale along the jth covariate and α denotes the marginal variance of the GP prior. Together they constitute the parameter vector $\boldsymbol{\phi} = [\alpha, \rho_1, \ldots, \rho_q]$.

Since our measurement noise is Gaussian with variance σ^2 we can analytically compute the discrepancy function to obtain the marginal likelihood of N observations $\boldsymbol{Y}_C = (Y_C)_{i=1}^N$, conditioned on the parameters $\boldsymbol{\theta}$, $\boldsymbol{\phi}$ of the mechanistic and discrepancy models respectively, as well as the calibration inputs u_C and $\boldsymbol{v}_C$, given by

$$p(\boldsymbol{Y}_C | u_C, \boldsymbol{v}_C, \boldsymbol{\theta}, \boldsymbol{\phi}) \sim \mathcal{N}(\mathbf{f}_{\boldsymbol{\theta}, u_C}, \Sigma_{NN} + \sigma^2 \boldsymbol{I}), \tag{S2.4}$$

where $\mathbf{f}_{\boldsymbol{\theta}, u_C} = [f_1(\boldsymbol{\theta}, u_C), \ldots, f_N(\boldsymbol{\theta}, u_C)]$ is a vector collecting the N evaluations of the mechanistic model function, Σ_{NN} represents the covariance function (Eq. S2.3) evaluated on all $N \times N$ pairs of the calibrations inputs $\boldsymbol{v}_C$:

$$\Sigma_{NN} = \begin{pmatrix} \boldsymbol{\kappa}(\boldsymbol{v}_C^1, \boldsymbol{v}_C^1; \boldsymbol{\phi}) & \ldots & \boldsymbol{\kappa}(\boldsymbol{v}_C^1, \boldsymbol{v}_C^N; \boldsymbol{\phi}) \\ \vdots & \ddots & \vdots \\ \boldsymbol{\kappa}(\boldsymbol{v}_C^N, \boldsymbol{v}_C^1; \boldsymbol{\phi}) & \cdots & \boldsymbol{\kappa}(\boldsymbol{v}_C^N, \boldsymbol{v}_C^N; \boldsymbol{\phi}) \end{pmatrix}, \tag{S2.5}$$

and $\boldsymbol{I}$ is a $N \times N$ identity matrix.

Inference of the model and GP parameters

We proceed by first placing suitable prior distributions, $p(\boldsymbol{\theta})$ and $p(\boldsymbol{\phi})$, on the model and GP parameters and then obtain the posterior distribution using Bayes theorem as follows:

$$p(\boldsymbol{\theta}, \boldsymbol{\phi} | \boldsymbol{Y}_C, u_C, \boldsymbol{v}_C) \propto p(\boldsymbol{Y}_C | u_C, \boldsymbol{v}_C, \boldsymbol{\theta}, \boldsymbol{\phi}) p(\boldsymbol{\theta}) p(\boldsymbol{\phi}). \tag{S2.6}$$

Since this posterior distribution is analytically intractable due to the non-linear dependence on $\boldsymbol{\theta}$ and $\boldsymbol{\phi}$ we resort to Markov chain Monte Carlo (MCMC) in order to obtain samples from this distribution.

Predictions

Having inferred the parameters $\boldsymbol{\theta}$ and $\boldsymbol{\phi}$ we may want to predict the output of the model in Eq. S2.1 for a new set of model inputs u_V and $\boldsymbol{v}_V$. Note that these new model inputs are considered as validation inputs, denoted with subscript V. For the purpose of derivation here,

we consider the number of validation points M to be different than the number of measurement points N, although these numbers can be the same for specific choices of calibrations.

We denote the column vector for the corresponding M predicted outputs as $\boldsymbol{Y}_V = (Y_V)_{i=1}^M$, and the model evaluations with the new inputs as $\mathbf{f}_{\boldsymbol{\theta},u_V} = [f_1(\boldsymbol{\theta}, u_V), \ldots, f_M(\boldsymbol{\theta}, u_V)]^T$ Furthermore, we denote the collection of calibration inputs at the N training (points corresponding to the measurements) as $\boldsymbol{I}_C = (u_C, \boldsymbol{v}_C)$, and at the prediction points as $\boldsymbol{I}_V = (u_V, \boldsymbol{v}_V)$

Note that for a fixed value of parameters, $\boldsymbol{\theta}$ and $\boldsymbol{\phi}$ respectively, we can analytically obtain the predictive distribution of $\boldsymbol{Y}_V$ given by

$$p(\boldsymbol{Y}_V|\boldsymbol{I}_V, \boldsymbol{I}_C, \boldsymbol{Y}_C, \boldsymbol{\theta}, \boldsymbol{\phi}) = \mathcal{N}(\boldsymbol{\mu_V}, \boldsymbol{\sigma_V^2}), \tag{S2.7}$$

where the mean and variance is given by [4]

$$\begin{aligned} \boldsymbol{\mu_V} &= \mathbf{f}_{\boldsymbol{\theta},u_V} + \Sigma_{MN}[\Sigma_{NN} + \sigma^2 \boldsymbol{I}]^{-1}(\boldsymbol{Y}_C - \mathbf{f}_{\boldsymbol{\theta},u_C}) \\ \boldsymbol{\sigma_V^2} &= \Sigma_{MM} - \Sigma_{MN}[\Sigma_{NN} + \sigma^2 \boldsymbol{I}]^{-1}\Sigma_{NM}, \end{aligned} \tag{S2.8}$$

where Σ_{MN} and Σ_{NM} denotes the $M \times N$ and $N \times M$ matrices of covariance function evaluations between the training and prediction inputs given by

$$\Sigma_{MN} = \begin{pmatrix} \boldsymbol{\kappa}(\boldsymbol{v}_V^1, \boldsymbol{v}_C^1; \boldsymbol{\phi}) & \ldots & \boldsymbol{\kappa}(\boldsymbol{v}_V^1, \boldsymbol{v}_C^N; \boldsymbol{\phi}) \\ \vdots & \ddots & \vdots \\ \boldsymbol{\kappa}(\boldsymbol{v}_V^M, \boldsymbol{v}_C^1; \boldsymbol{\phi}) & \cdots & \boldsymbol{\kappa}(\boldsymbol{v}_V^M, \boldsymbol{v}_C^N; \boldsymbol{\phi}) \end{pmatrix}, \tag{S2.9}$$

$$\Sigma_{NM} = \begin{pmatrix} \boldsymbol{\kappa}(\boldsymbol{v}_C^1, \boldsymbol{v}_V^1; \boldsymbol{\phi}) & \ldots & \boldsymbol{\kappa}(\boldsymbol{v}_C^1, \boldsymbol{v}_V^M; \boldsymbol{\phi}) \\ \vdots & \ddots & \vdots \\ \boldsymbol{\kappa}(\boldsymbol{v}_C^N, \boldsymbol{v}_V^1; \boldsymbol{\phi}) & \cdots & \boldsymbol{\kappa}(\boldsymbol{v}_C^N, \boldsymbol{v}_V^M; \boldsymbol{\phi}) \end{pmatrix}, \tag{S2.10}$$

with inputs $\boldsymbol{v}_C$ and $\boldsymbol{v}_V$, respectively, and Σ_{MM} is the covariance evaluated at the prediction inputs $\boldsymbol{v}_V$ only:

$$\Sigma_{MM} = \begin{pmatrix} \boldsymbol{\kappa}(\boldsymbol{v}_V^1, \boldsymbol{v}_V^1; \boldsymbol{\phi}) & \ldots & \boldsymbol{\kappa}(\boldsymbol{v}_V^1, \boldsymbol{v}_V^M; \boldsymbol{\phi}) \\ \vdots & \ddots & \vdots \\ \boldsymbol{\kappa}(\boldsymbol{v}_V^M, \boldsymbol{v}_V^1; \boldsymbol{\phi}) & \cdots & \boldsymbol{\kappa}(\boldsymbol{v}_V^M, \boldsymbol{v}_V^M; \boldsymbol{\phi}) \end{pmatrix}. \tag{S2.11}$$

Finally, to obtain the marginal (i.e. integrating out the parameters) predictive distribution:

$$p(\boldsymbol{Y}_V|\boldsymbol{I}_V, \boldsymbol{I}_C, \boldsymbol{Y}_C) = \int \mathcal{N}(\boldsymbol{\mu_V}, \boldsymbol{\sigma_V^2}) p(\boldsymbol{\theta}, \boldsymbol{\phi}|\boldsymbol{Y}_C, u_C, \boldsymbol{v}_C)\, \mathrm{d}\boldsymbol{\theta}\, \mathrm{d}\boldsymbol{\phi}, \tag{S2.12}$$

we use Monte Carlo integration using the samples of $\boldsymbol{\theta}$ and $\boldsymbol{\phi}$ obtained through MCMC.

Sparse Gaussian Process

The above formulation of the discrepancy model suffers from a crucial computational bottleneck stemming from the need of inverting the covariance matrix Σ_{NN} while evaluating the marginal likelihood in Eq. S2.4, as well as drawing posterior predictions in Eq. S2.12 (in turn using Eq. S2.8). In all the calibration problems under consideration here, we have a large number of data points (time series measurements) where $N \geq 80000$. Thus, it becomes infeasible to apply Gaussian processes for modelling the discrepancy without tackling this excessive computational load related to repeated inversion of a large matrix.

In order to alleviate this computational bottleneck we use a sparse approximation of the true covariance function. Quiñonero-Candela et al. [5] provides an extensive review of such sparse approximations techniques. Following [5] we use a set of P or inducing inputs (or pseudo-inputs) x_C with associated latent function $\delta(\boldsymbol{\phi}, x_C)$ representing the discrepancy function corresponding

to the inducing inputs. This inducing function is assigned a zero mean GP prior as follows:

$$\delta(\boldsymbol{\phi}, x_C) \sim \mathcal{GP}\big(0, \boldsymbol{\kappa}(x_C, x_C'; \boldsymbol{\phi})\big). \tag{S2.13}$$

Let us denote the vector of discrepancy function evaluations at all the training points as $\delta_{\boldsymbol{\phi},u_C} = [\delta_1(\boldsymbol{\phi}, u_C), \ldots, \delta_N(\boldsymbol{\phi}, u_C)]^T$ and at inducing points as $\delta_{\boldsymbol{\phi},x_C} = [\delta_1(\boldsymbol{\phi}, x_C), \ldots, \delta_P(\boldsymbol{\phi}, x_C)]^T$. We can then write the joint prior as a product of all the training and inducing points as $p(\delta_{\boldsymbol{\phi},u_C}) = \mathcal{N}(\mathbf{0}, \Sigma_{NN})$ and $p(\delta_{\boldsymbol{\phi},x_C}) = \mathcal{N}(\mathbf{0}, \Sigma_{PP})$ respectively, where Σ_{PP} denotes the covariance evaluated at all pairs of inducing inputs:

$$\Sigma_{PP} = \begin{pmatrix} \boldsymbol{\kappa}(x_C^1, x_C^1; \boldsymbol{\phi}) & \ldots & \boldsymbol{\kappa}(x_C^1, x_C^P; \boldsymbol{\phi}) \\ \vdots & \ddots & \vdots \\ \boldsymbol{\kappa}(x_C^P, x_C^1; \boldsymbol{\phi}) & \cdots & \boldsymbol{\kappa}(x_C^P, x_C^P; \boldsymbol{\phi}) \end{pmatrix}. \tag{S2.14}$$

We can then approximate the prior on the true discrepancy function $\delta_{\boldsymbol{\phi},u_C}$ marginalising the inducing discrepancies as:

$$\begin{aligned} p(\delta_{\boldsymbol{\phi},u_C}) \approx p(\delta_{\boldsymbol{\phi},u_C} | \delta_{\boldsymbol{\phi},x_C}) &= \int p(\delta_{\boldsymbol{\phi},u_C} | \delta_{\boldsymbol{\phi},x_C}) p(\delta_{\boldsymbol{\phi},x_C}) \, \mathrm{d}\, \delta_{\boldsymbol{\phi},x_C} \\ &= \mathcal{N}(\Sigma_{NP}\Sigma_{PP}^{-1}\delta_{\boldsymbol{\phi},x_C}, \Sigma_{NN} - \Sigma_{NP}\Sigma_{PP}^{-1}\Sigma_{PN}), \end{aligned} \tag{S2.15}$$

where Σ_{NP}, Σ_{PN} denotes the covariance matrices containing the cross-covariances between the training and inducing inputs (evaluated in the same way as in Eqs. S2.9, S2.10). This sparse approximation was first introduced in [6] to scale the GP regression model. This approximation is widely known as the *fully independent training conditional* (FITC) approximation in machine learning parlance since the introduction of these inducing inputs and corresponding function values $\delta_{\boldsymbol{\phi},x_C}$ induces a conditional independence among all the elements of $\delta_{\boldsymbol{\phi},u_C}$ [5], that is we have

$$p(\delta_{\boldsymbol{\phi},u_C} | \delta_{\boldsymbol{\phi},x_C}) = \prod_{i=1}^{N} p(\delta_i(\boldsymbol{\phi}, \boldsymbol{v}_C^i) | \delta_{\boldsymbol{\phi},x_C}) = \mathcal{N}(\Sigma_{NP}\Sigma_{PP}^{-1}\delta_{\boldsymbol{\phi},x_C}, \Sigma_{NN} - \Sigma_{NP}\Sigma_{PP}^{-1}\Sigma_{PN}). \tag{S2.16}$$

Using this approximate prior $p(\delta_{\boldsymbol{\phi},u_C} | \delta_{\boldsymbol{\phi},x_C})$ to obtain the marginal likelihood and the prediction terms we essentially approximate the true covariance Σ_{NN} as [5]:

$$\Sigma_{NN} \approx \hat{\Sigma} = \boldsymbol{Q} + \mathrm{diag}(\Sigma_{NN} - \boldsymbol{Q}), \tag{S2.17}$$

where $\mathrm{diag}(A)$ is a diagonal matrix whose elements match the diagonal of A and the matrix $\mathbf{Q}$ is given by

$$\boldsymbol{Q} = \Sigma_{NP}\Sigma_{PP}^{-1}\Sigma_{PN}. \tag{S2.18}$$

$\hat{\Sigma}$ has the same diagonal elements as Σ_{NN} and the off-diagonal elements are the same as for $\boldsymbol{Q}$. Thus, inversion of $\hat{\Sigma}$ scales as $\mathcal{O}(NP^2)$ as opposed to $\mathcal{O}(N^3)$ for the inversion of Σ_{NN}.

S3. Modelling residuals using an $\mathrm{ARMA}(p,q)$ process

In the previous section we modelled the discrepancy as a function drawn from a GP prior. Alternatively, we can address the case of discrepancy using a correlated residual approach (see Section 3.6.2 in [7] for an introduction to this modelling approach). In this case we can model the residuals between the data $(Y_C)_i$ and the mechanistic model $f_i(\boldsymbol{\theta}, u_C^i)$ as an $\mathrm{ARMA}(p,q)$ process as follows:

$$\begin{aligned}(Y_C)_i - f_i(\boldsymbol{\theta}, u_C^i) &= e_i \\ &= \varphi_1 e_{i-1} + \ldots + \varphi_p e_{i-p} + \nu_i + \zeta_1 \nu_{i-1} + \ldots + \zeta_q \nu_{i-1-q},\end{aligned} \tag{S3.1}$$

where

$$\nu_i \sim \mathcal{N}(0, \tau^2), \tag{S3.2}$$

and $\boldsymbol{\varphi} = [\varphi_1, \ldots, \varphi_p]^T$, $\boldsymbol{\zeta} = [\zeta_1, \ldots, \zeta_p]^T$ are the vectors representing the $p \geq 0$ autoregressive coefficients and $q \geq 0$ moving-average coefficients of the ARMA process.

The rationale behind this modelling approach comes from the fact that if the mechanistic model is able to explain the measurements adequately then the residuals are essentially uncorrelated measurement noise $\epsilon \sim \mathcal{N}(0, \sigma^2)$. Note that we use a different symbol ν, as opposed to ϵ, to represent the noise term in order to highlight the difference in its interpretations. However, the existence of discrepancy between the model output and the observations points to the fact that the residuals, for each data sample, has unexplained structure that can be modelled using a pre-determined correlation structure, as expressed through an $\mathrm{ARMA}(p,q)$ model.

Inference

We first re-write the normally distributed error term ν_i as

$$\nu_i = (Y_C)_i - f_i(\boldsymbol{\theta}, u_C^i) - \sum_{j=1}^{p} \varphi_j \{(Y_C)_{i-j} - f_{i-j}(\boldsymbol{\theta}, u_C^{i-j})\} - \sum_{k=1}^{q} \zeta_k \nu_{i-k}, \tag{S3.3}$$

using which we can write the conditional likelihood of the observed data for N measurements as [8]

$$p(\boldsymbol{Y}_C | \boldsymbol{\theta}, \boldsymbol{\varphi}, \boldsymbol{\zeta}, \tau) = (2\pi\tau^2)^{N/2} \exp\left(-\frac{1}{2\tau^2} \sum_{i=p+1}^{N} \nu_i^2 \right), \tag{S3.4}$$

where we have used ν_i for $i \geq p+1$ by assuming that $\nu_p = \nu_{p-1} = \ldots = \nu_{p+1-q} = 0$, its expected value. Note that to calculate the likelihood for all the N measurements requires us to introduce extra parameter values, as latent variables, for the past history of the data as well as the error terms before measurement commences, that is for $[(Y_C)_0, (Y_C)_{-1}, \ldots, (Y_C)_{1-p}]$ and $[\nu_0, \nu_{-1}, \ldots, \nu_{1-q}]$. Alternatively, we can reformulate Eq. S3.1 in a state space form and use the Kalman filter algorithm to evaluate the unconditional full likelihood for all i. We refer the reader to [7] for the details of this approach. We point out here that the difference between these two approaches of calculating the likelihood is insignificant for long time series, which is the case for our calibration problems with $N \geq 80000$.

Having defined the likelihood we can again adopt the Bayesian framework to infer posterior distributions of the model parameters $\boldsymbol{\theta}$ and the set of ARMA parameters $\boldsymbol{\phi} = [\boldsymbol{\varphi}, \boldsymbol{\zeta}]$, by choosing suitable prior distributions $p(\boldsymbol{\theta})$ and $p(\boldsymbol{\phi})$ respectively and using the Bayes theorem to obtain the posterior given by

$$p(\boldsymbol{\theta}, \boldsymbol{\phi} | \boldsymbol{Y}_C) \propto p(\boldsymbol{Y}_C | \boldsymbol{\theta}, \boldsymbol{\phi}) p(\boldsymbol{\theta}) p(\boldsymbol{\phi}). \tag{S3.5}$$

Note that we have considered the noise variance known since its maximum likelihood estimate is given by $\tau^2 = \frac{\sum_{i=p+1}^{N} \nu_i^2}{N-(2p+q+1)}$, which can be easily obtained once estimates of $\boldsymbol{\phi}$ and $\boldsymbol{\theta}$ are available. Similar to the GP based inference problem we again use MCMC to obtain the desired posterior distributions.

Predictions

In a purely time series modelling context, where models such as the ARMA is extensively used, predictions are used to forecast ahead in time for short intervals. In the context of our calibration problem we are generally interested in predicting outputs for a new calibration u_V. However, considering the fact that we want to to predict M output values corresponding to the new validation inputs, we can simply recast our predictions as one-step-ahead forecasts.

We denote the M predicted outputs as $\boldsymbol{Y}_V = (Y_V)_{m=1}^M$, while $\boldsymbol{Y}_p = (Y_C)_{N-(p-1)}^N$ and $\mathbf{f}_{\boldsymbol{\theta},u_C} = [f_N(\boldsymbol{\theta}, u_C), \ldots, f_{N-(p-1)}(\boldsymbol{\theta}, u_C)]$ are column vectors representing the last p observations and model evaluations with the calibration u_C. We denote the vector of the last q errors as $\boldsymbol{\nu}_C = [\nu_{N-(q-1)}, \ldots, \nu_N]^T$.

Note that in our formulation here, the mth, $m = 1, \ldots, M$, prediction is to be considered as the $(N+1)^{\text{th}}$ prediction from the model in Eq. S3.1 with the following modification: we replace $f_{N+1}(\boldsymbol{\theta}, u_C^i)$ with $f_1(\boldsymbol{\theta}, u_V^1)$. Thus, for a particular value of the parameters we have

$$(Y_V)_m \sim \mathcal{N}(\mathbb{E}[(Y_V)_m|\boldsymbol{Y}_p, \boldsymbol{\nu}_C, \boldsymbol{\theta}, \boldsymbol{\phi}], \mathrm{Var}[(Y_V)_m|\boldsymbol{Y}_p, \boldsymbol{\nu}_C, \boldsymbol{\theta}, \boldsymbol{\phi}]), \tag{S3.6}$$

where the mean and the variance of the one-step ahead prediction distribution is given by

$$\begin{aligned} \mathbb{E}[(Y_V)_m|\boldsymbol{Y}_p, \boldsymbol{\nu}_C, \boldsymbol{\theta}, \boldsymbol{\phi}] &= f_m(\boldsymbol{\theta}, u_V^m) + \boldsymbol{\varphi}^T(\boldsymbol{Y}_p - \mathbf{f}_{\boldsymbol{\theta},u_C}) + \boldsymbol{\zeta}^T \boldsymbol{\nu}_C, \\ \mathrm{Var}[(Y_V)_m|\boldsymbol{Y}_p, \boldsymbol{\nu}_C, \boldsymbol{\theta}, \boldsymbol{\phi}] &= \mathrm{Var}\left[(Y_V)_m - \mathbb{E}[(Y_V)_m|\boldsymbol{Y}_p, \boldsymbol{\nu}_C, \boldsymbol{\theta}, \boldsymbol{\phi}]\right] = \mathrm{Var}[\nu_N] = \tau^2. \end{aligned} \tag{S3.7}$$

In order to quantify the uncertainty in the predictions we can integrate out the model and noise parameters [9]:

$$p((Y_V)_m|\boldsymbol{Y}_p, \boldsymbol{\nu}_C) = \int \mathcal{N}(\mathbb{E}[(Y_V)_m|\boldsymbol{Y}_p, \boldsymbol{\nu}_C, \boldsymbol{\theta}, \boldsymbol{\phi}] p(\boldsymbol{\theta}, \boldsymbol{\phi}|\boldsymbol{Y}_C)\, \mathrm{d}\,\boldsymbol{\theta}\, \mathrm{d}\,\boldsymbol{\phi}, \tag{S3.8}$$

where we use Monte Carlo integration as in Eq. S2.12.

In order to collect the full set of M predictions $\boldsymbol{Y}_V$ we simply use the one-step-ahead forecasting distribution shown above in a recursive manner.

S4. Choice of priors for the ion channel example

Here we specify the choice of priors for the ion channel example.

- For the ion channel ODE model parameters, we chose a uniform prior specified in Beattie *et al.* [10] and Lei *et al.* [11,12].
- For the GP model, we have the unbiased noise parameter σ, the length-scale ρ_i, the marginal variance α:
  - σ: Half-Normal prior with standard deviation of 25;
  - ρ_i: Inverse-Gamma prior with shape and scale being $(5, 5)$;
  - α: Inverse-Gamma prior with shape and scale being $(5, 5)$.
- For the ARMA model, we have the autoregressive coefficients φ_i, and moving-average coefficients ζ_i:
  - φ_i: Normal prior centred on the maximum likelihood estimates with standard deviation of 2.5;
  - ζ_i: Normal prior centred on the maximum likelihood estimates with standard deviation of 2.5.

S5. Computing and representing posterior predictive

To compute the posterior predictive, we follow Girolami [13] and write the posterior predictive in Eqs. S2.12 & S3.8 as

$$p(Y_V \mid Y_C) = \sum_k p(Y_V \mid \boldsymbol{\theta}_k, \boldsymbol{\phi}_k, Y_C) p(\boldsymbol{\theta}_k, \boldsymbol{\phi}_k \mid Y_C), \tag{S5.1}$$

where $\boldsymbol{\theta}_k, \boldsymbol{\phi}_k$ are the kth posterior sample of the parameters. We have checked the posterior predictive of the ODE models at a given time point is symmetric and similar to a Gaussian distribution, for the sake of simplicity, we therefore use summary statistics such as the predictive mean and credible intervals computed using variance to represent the posterior predictive in this paper. To obtain the predictive mean $\mathbb{E}[Y_V \mid Y_C]$ and variance $\mathrm{Var}[Y_V \mid Y_C]$, we use

$$\mathbb{E}[Y_V \mid Y_C] = \sum_k \mathbb{E}[Y_V \mid \boldsymbol{\theta}_k, \boldsymbol{\phi}_k, Y_C] p(\boldsymbol{\theta}_k, \boldsymbol{\phi}_k \mid Y_C), \tag{S5.2}$$

$$\mathrm{Var}[Y_V \mid Y_C] = \sum_k \left(\mathrm{Var}[Y_V \mid \boldsymbol{\theta}_k, \boldsymbol{\phi}_k, Y_C] + \mathbb{E}[Y_V \mid \boldsymbol{\theta}_k, \boldsymbol{\phi}_k, Y_C]^2 \right) p(\boldsymbol{\theta}_k, \boldsymbol{\phi}_k \mid Y_C) - \mathbb{E}[Y_V \mid Y_C]^2. \tag{S5.3}$$

Finally, to show the 95% credible intervals of our predictions, we plot $\mathbb{E}[Y_V \mid Y_C] \pm 1.96\sigma_{Y_V}$ where $\sigma^2_{Y_V} = \mathrm{Var}[Y_V \mid Y_C]$.

S6. Supplementary results for the action potential example

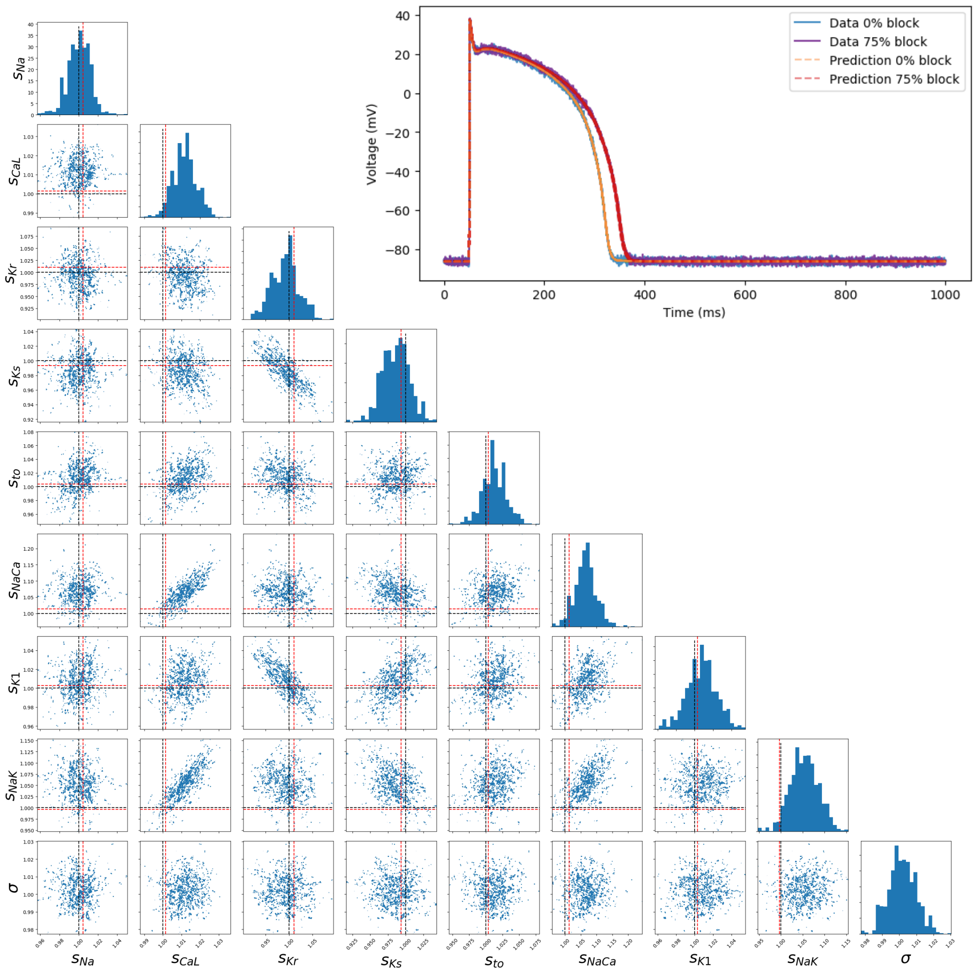

Figure S1. **Matrix plot and histograms**: Posterior distribution of Model T parameters when estimated using data from Model T. The dashed black lines indicate the true (data-generating) parameters; the dashed red lines are the result of the global optimisation routine. **Inset plot**: Posterior predictions for the 'context of use' (CoU) data, for the action potential model tutorial (in the scenario of no model discrepancy). The posterior predictions are model predictions using parameters sampled from the posterior distribution; 200 samples/predictions are shown. Model T gives an almost-perfect prediction of the CoU data (which was not used in training).

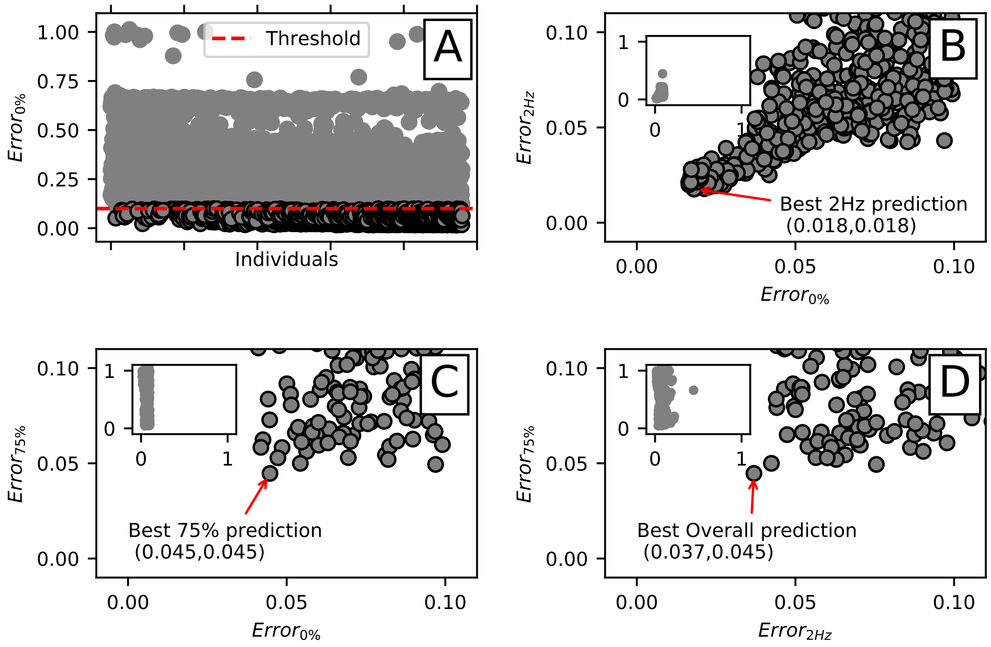

Figure S2. Results obtained via Genetic Algorithm and Differential Evolution to adjust the Fink Action Potential to ten Tusscher at control, i.e., $1\,\mathrm{Hz}$, and 0% IKr block configuration. The Genetic Algorithm was used with a population size of 100 individuals and 10 generations. The Differential Evolution was used with 150 individuals and 15 generations. Both algorithms were implemented using the Python library Pygmo with the standard configurations. **A** From all the evaluations we selected the candidates (parameter sets) that satisfied $Error_{0\%} < 0.1$. Relative RMS errors are computed with $Error_X(i) = \frac{||T_X - aF_X(i)||_2}{||T_X||_2}$, where T_X is the ten Tusscher model in scenario X, and aF_X is the adjusted Fink model using the individual i for scenario X. A total of 1079 candidates satisfied $Error_{0\%} < 0.1$, i.e., were below the displayed threshold. Using this metric, the best candidate had $Error_{0\%} = 1.6\%$. **B** Testing the performance of the 1079 candidates with respect to the 2Hz scenario a total of 990 candidates satisfy $Error_{0\%} < 0.1$ and $Error_{2Hz} < 0.1$. Using these two metrics, the best candidate had $Error_{0\%} = 1.8\%$ and $Error_{2Hz} = 1.8\%$. **C** Testing the performance of the 1079 candidates with respect to the 75% IKr block scenario only 80 candidates satisfy $Error_{0\%} < 0.1$ and $Error_{75\%} < 0.1$. Using these two metrics, the best candidate had $Error_{0\%} = 4.5\%$ and $Error_{75\%} = 4.5\%$. **D** Testing the performance of the 1079 candidates with respect to both 75% IKr block and 2Hz scenarios only 70 candidates satisfy $Error_{0\%} < 0.1$, $Error_{75\%} < 0.1$, and $Error_{2Hz} < 0.1$. Using the three metrics, the best candidate had $Error_{0\%} = 4.5\%$, $Error_{2Hz} = 3.7\%$, and $Error_{75\%} = 4.5\%$.

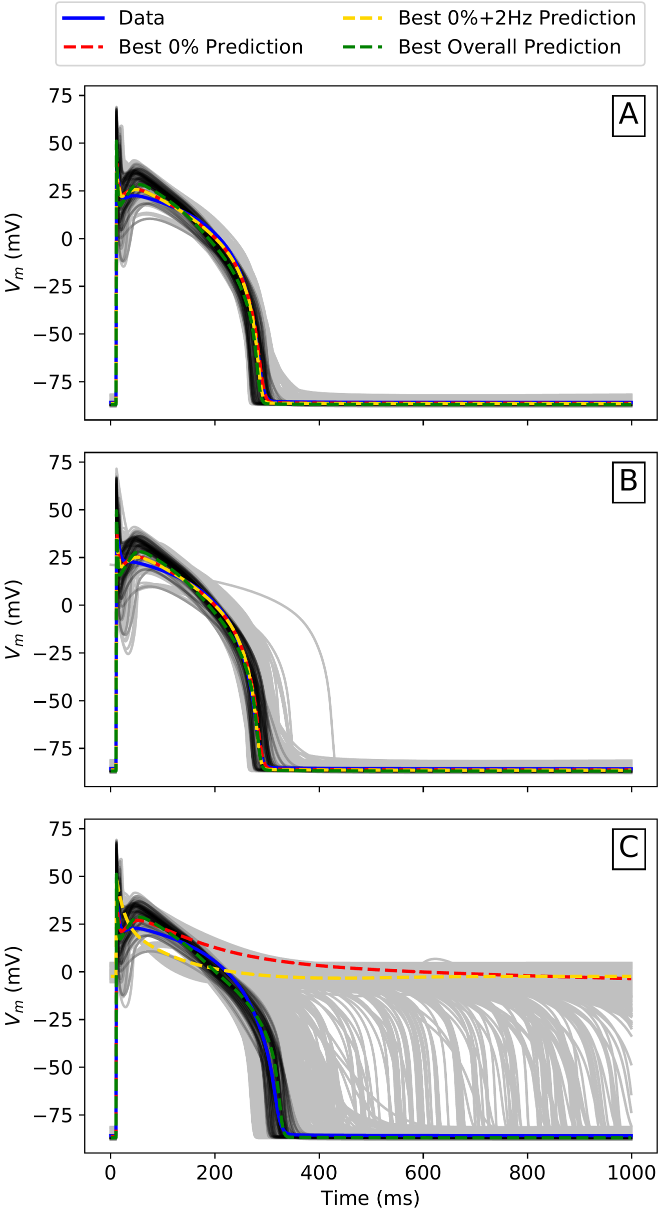

Figure S3. APs obtained by the Data (ten Tusscher model) and also by the fitting process for each scenario: **A** control, i.e., $1\,\mathrm{Hz}$, and 0% IKr block; **B** $2\,\mathrm{Hz}$; and **C** 75% IKr block. "Best 0% prediction" are the results obtained using the best candidate that satisfies $Error_{0\%} < 0.1$. "Best $0\% + 2Hz$ prediction" are results obtained using the best candidate that satisfies $Error_{0\%} < 0.1$ and $Error_{2Hz} < 0.1$. "Best overall prediction" are results obtained using the best candidate that satisfies $Error_{0\%} < 0.1$, $Error_{2Hz} < 0.1$, and $Error_{75\%} < 0.1$. The 1079 APs that satisfy $Error_{0\%} < 0.1$ are plotted with grey lines. The 70/1079 APs that satisfy $Error_{0\%} < 0.1$, $Error_{2Hz} < 0.1$, and $Error_{75\%} < 0.1$ are are plotted with black lines. Note that there is no way of knowing in advance what the best candidate parameter set will be without performing the experiment, so a distribution of possibilities should generally be shown.

S7. Supplementary results for the ion channel discrepancy example

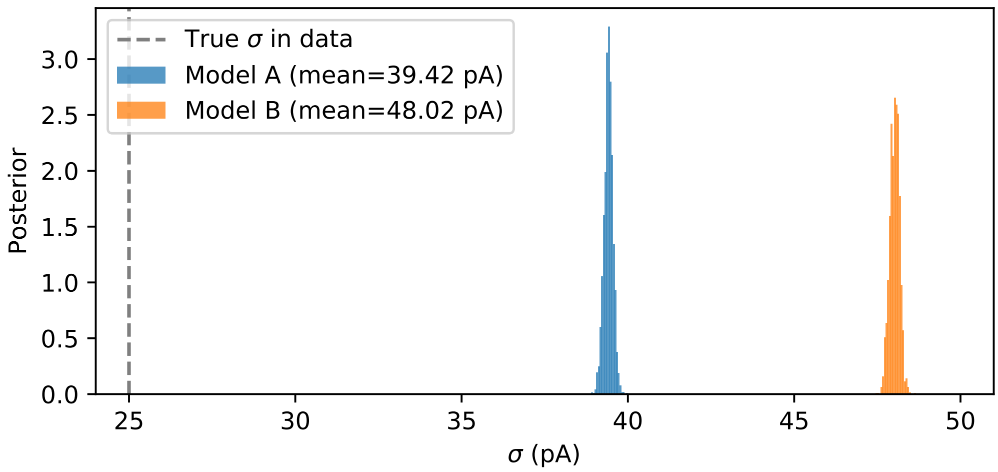

Figure S4. A comparison of the σ values in the i.i.d. model for Model A and B, and σ_{true} refers to the value used in generating the data with Model C. If we consider the inferred σ value in Eq. 1.3 in the main text as $\sigma^2_{\text{inferred}} = \sigma^2_{\text{true}} + \sigma^2_{\text{discrepancy}}$, then we can see that both $\sigma_{\text{A}}, \sigma_{\text{B}} > \sigma_{\text{true}}$. Hence we have $\sigma^2_{\text{discrepancy}}$ term is non-zero for both models, which reflects the fact that there is discrepancy for both models. One may use the size of σ_{inferred} to interpret the size of the model discrepancy here.

Model A		iid noise	GP(t)	GP(O, V)	ARMA(2, 2)
Calibration	Sinewave	-3.8×10^4	-2.1×10^3	-1.4×10^4	0
Prediction	Staircase	-5.4×10^5	-3.4×10^5	-3.6×10^5	0
	AP	-6.1×10^4	-2.2×10^5	-1.1×10^5	0

Model B		iid noise	GP(t)	GP(O, V)	ARMA(2, 2)
Calibration	Sinewave	-5.5×10^4	-6.8×10^3	-1.9×10^4	0
Prediction	Staircase	-1.6×10^6	-6.6×10^5	-3.7×10^5	0
	AP	-3.6×10^5	-3.8×10^5	0	-2.5×10^4

Table S1. Posterior predictive log-likelihoods of fits and predictions for Models A (left) and B (right) with different discrepancy models: i.i.d. noise, $\mathrm{GP}(t)$, $\mathrm{GP}(O, V)$, and $\mathrm{ARMA}(2, 2)$ for all three voltage protocols. The posterior predictive log-likelihood is $\pi(Y \mid Y_C) = \int \pi(Y \mid \theta)\pi(\theta \mid Y_C)\mathrm{d}\theta$, which we approximate by $\frac{1}{N}\sum_{n=1}^{N} p(Y \mid \theta_n)$ where θ_n are samples from the posterior distribution generated by MCMC. Only relative differences within a row are meaningful, and we therefore subtract the maximum log-likehood for each dataset from the results giving the best model in each row a score of zero. Note that care is needed when interpreting the log-likelihood values for the GP models due to the FITC approximation used to approximate the full likelihood.

(a) Model A

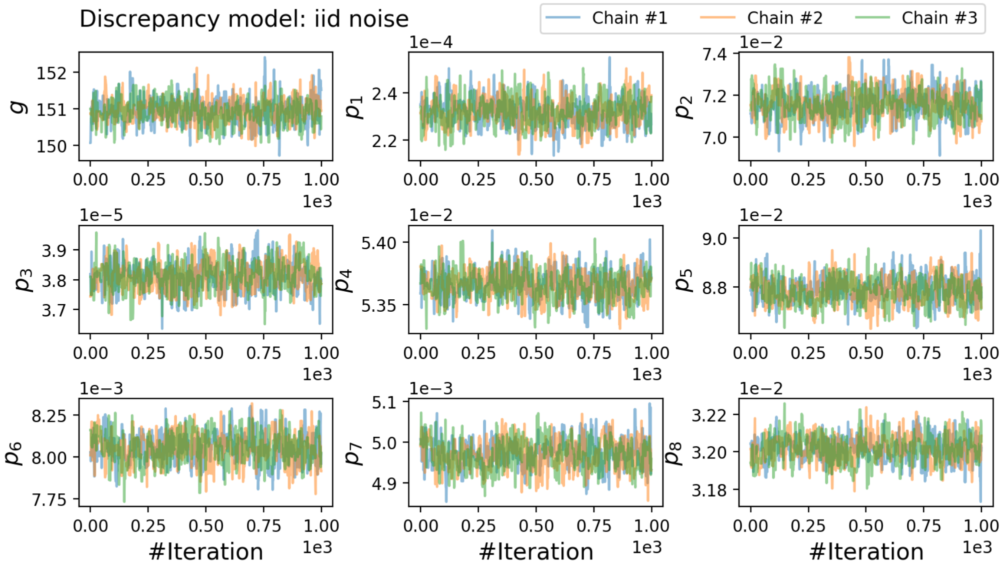

Figure S5. Trace plot of 3 independent MCMC runs for Model A parameters (with the i.i.d. noise model): the conductance, g, and kinetic parameters $p_1, \ldots, p_8$ (a list of parameters referring to $A_{i,j}$ and $B_{i,j}$ in Eq. (3.5)).

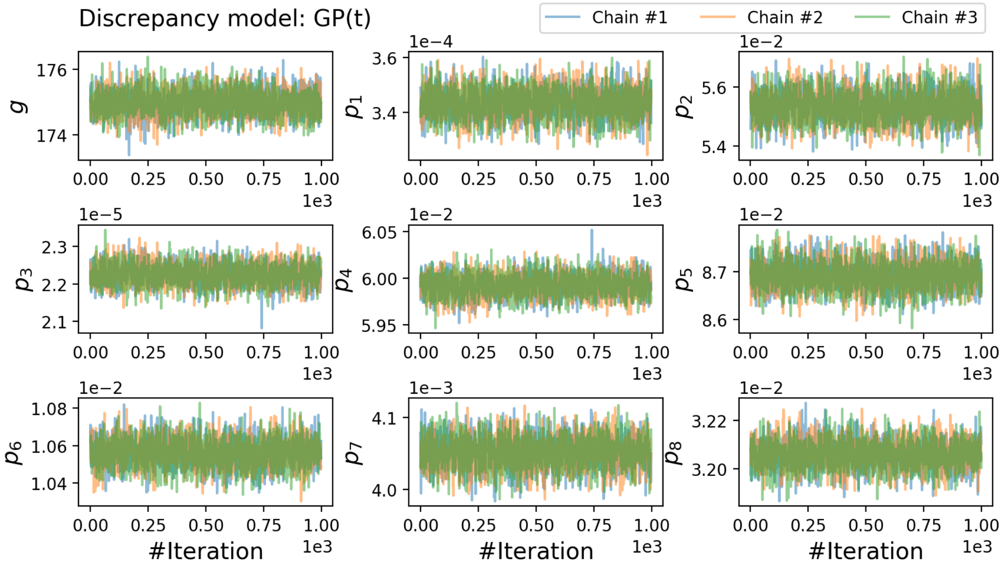

Figure S6. Trace plot of 3 independent MCMC runs for Model A parameters (with the $\mathrm{GP}(t)$ noise discrepancy model): the conductance, g, and kinetic parameters $p_1, \ldots, p_8$ (a list of parameters referring to $A_{i,j}$ and $B_{i,j}$ in Eq. (3.5)).

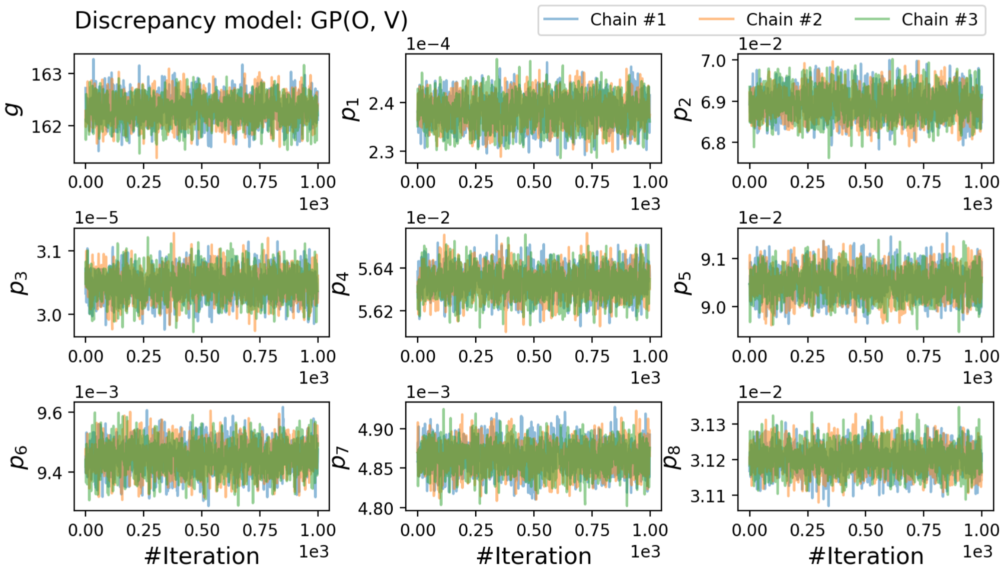

Figure S7. Trace plot of 3 independent MCMC runs for Model A parameters (with the $\mathrm{GP}(O, V)$ noise discrepancy model): the conductance, g, and kinetic parameters $p_1, \ldots, p_8$ (a list of parameters referring to $A_{i,j}$ and $B_{i,j}$ in Eq. (3.5)).

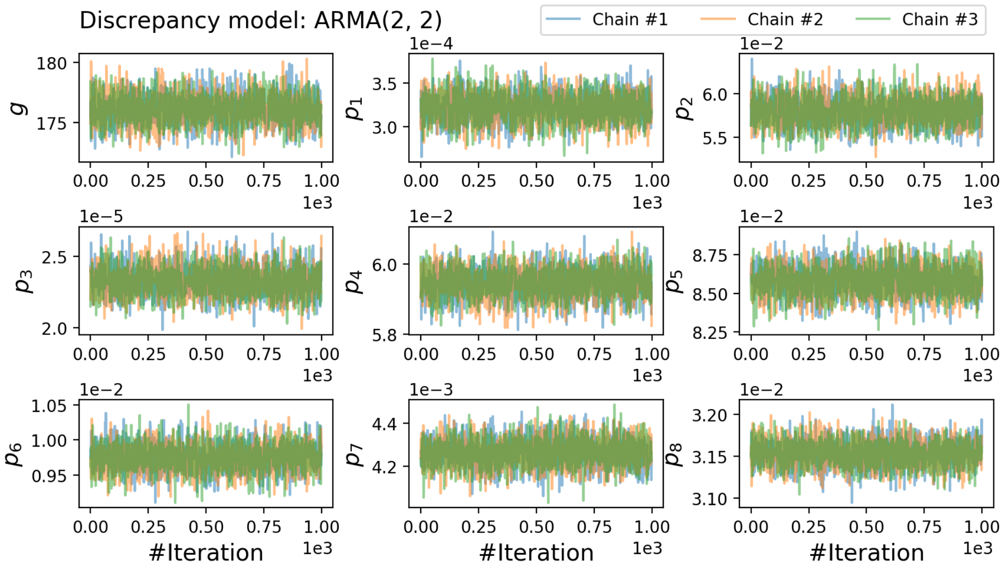

Figure S8. Trace plot of 3 independent MCMC runs for Model A parameters (with the $\mathrm{ARMA}(2, 2)$ noise discrepancy model): the conductance, g, and kinetic parameters $p_1, \ldots, p_8$ (a list of parameters referring to $A_{i,j}$ and $B_{i,j}$ in Eq. (3.5)).

(i) Model A: Full model predictions

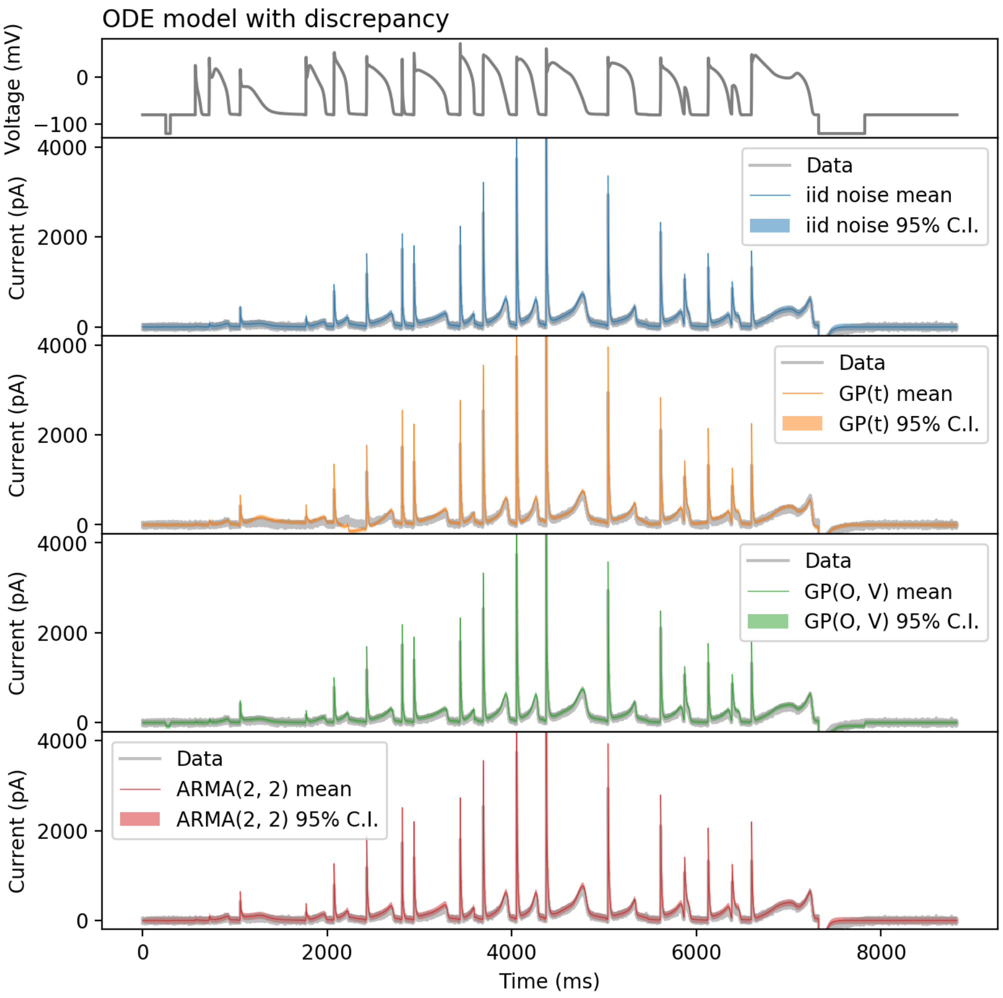

Figure S9. Model A prediction with different discrepancy models: no discrepancy (i.i.d. noise), $\mathrm{GP}(t)$, $\mathrm{GP}(O, V)$, and $\mathrm{ARMA}(2, 2)$. The voltage clamp protocol for calibration is the action potential series protocol [10].

(ii) Model A: Discrepancy predictions

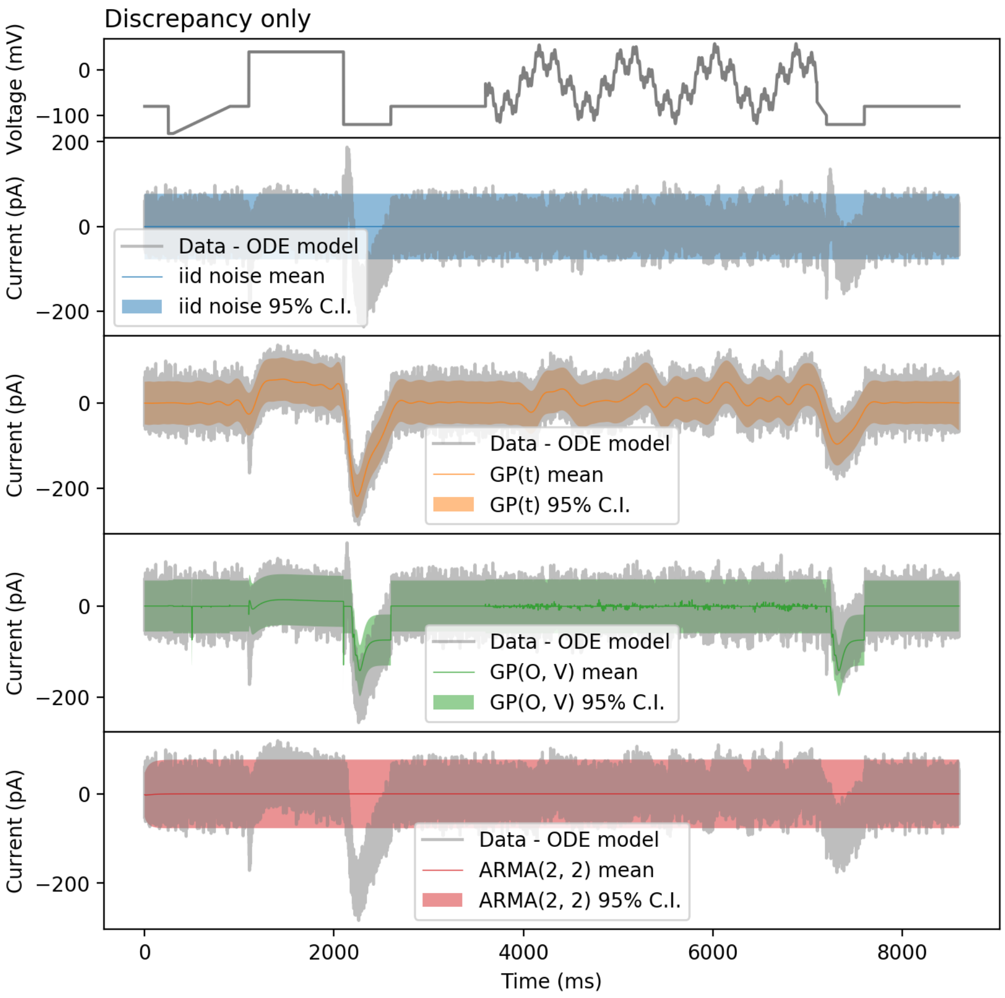

Figure S10. Model A fitting residuals of the MAP estimate accounted by different discrepancy models: no discrepancy (i.i.d. noise), $\mathrm{GP}(t)$, $\mathrm{GP}(O, V)$, and $\mathrm{ARMA}(2, 2)$. The voltage clamp protocol for calibration is the sinusoidal protocol [10].

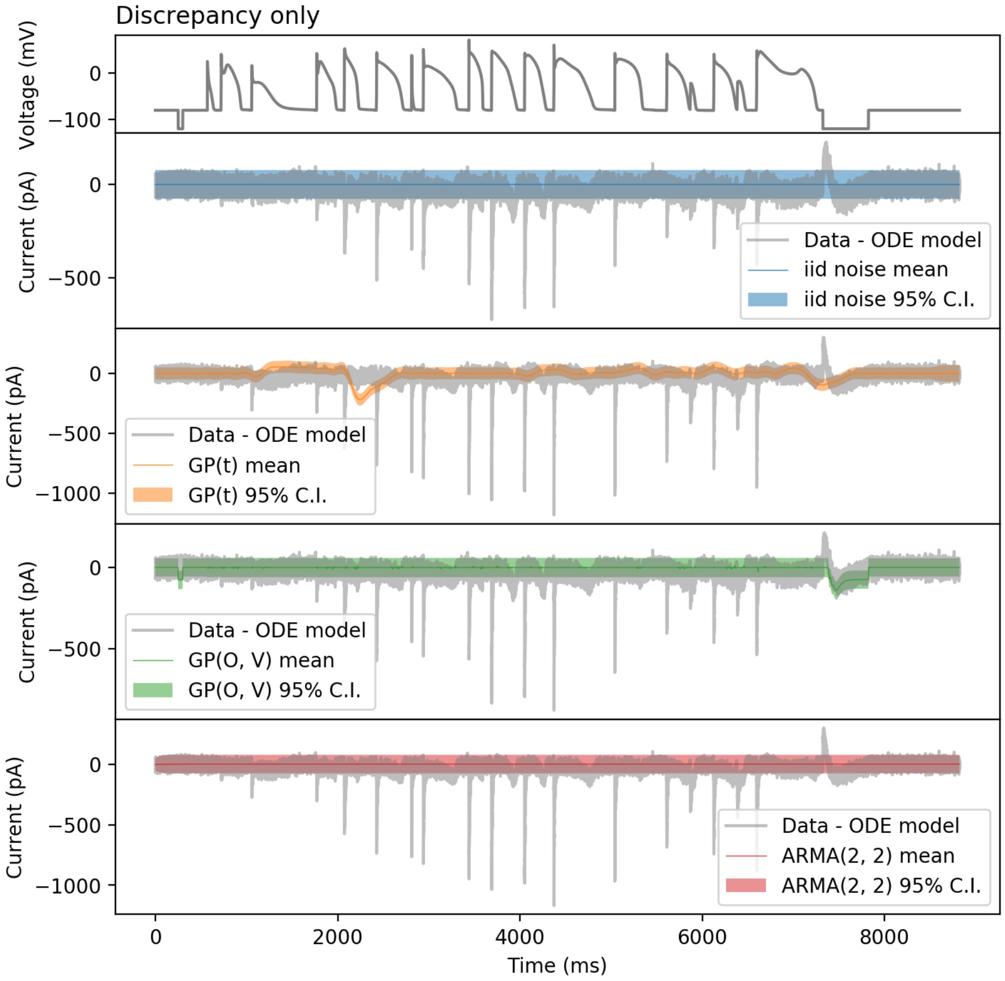

Figure S11. Model A prediction residuals of the MAP estimate accounted by different discrepancy models: no discrepancy (i.i.d. noise), $\mathrm{GP}(t)$, $\mathrm{GP}(O, V)$, and $\mathrm{ARMA}(2, 2)$. The voltage clamp protocol for calibration is the action potential series protocol [10].

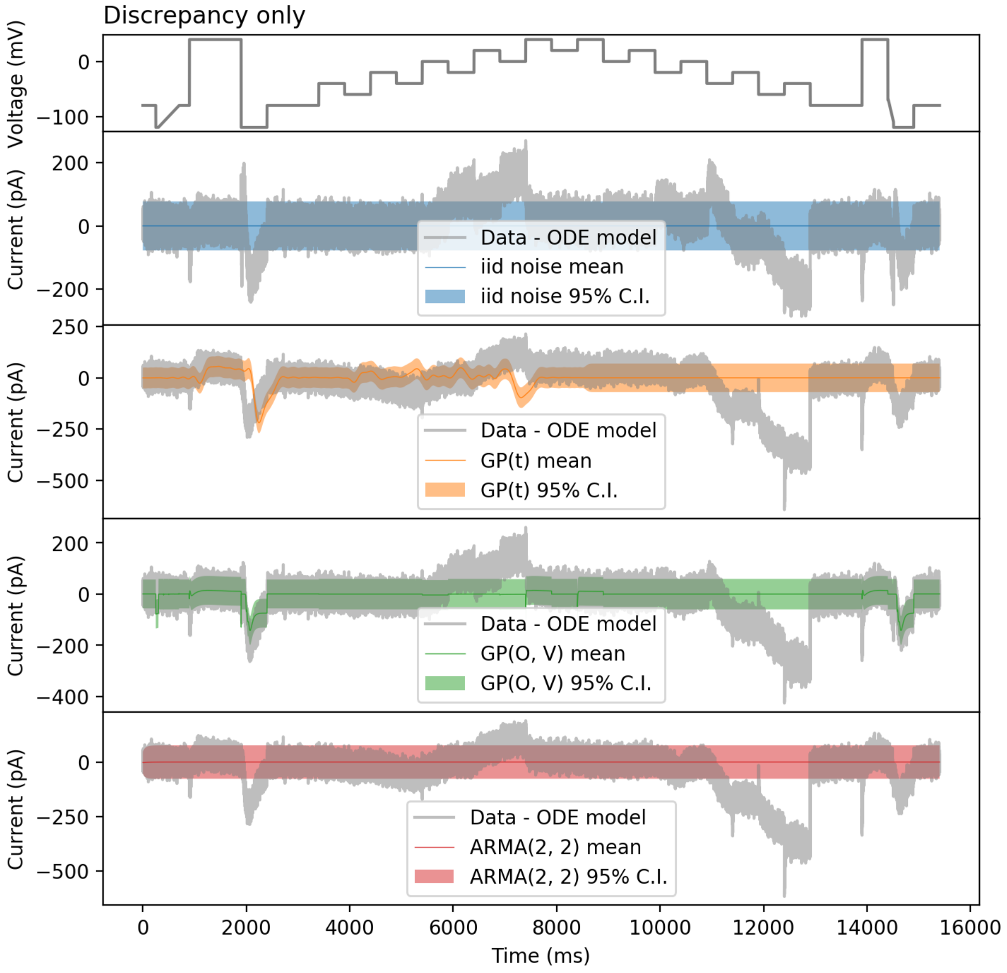

Figure S12. Model A prediction residuals of the MAP estimate accounted by different discrepancy models: no discrepancy (i.i.d. noise), $\mathrm{GP}(t)$, $\mathrm{GP}(O, V)$, and $\mathrm{ARMA}(2, 2)$. The voltage clamp protocol for calibration is the staircase protocol [11].

(iii) Model A: ODE model predictions

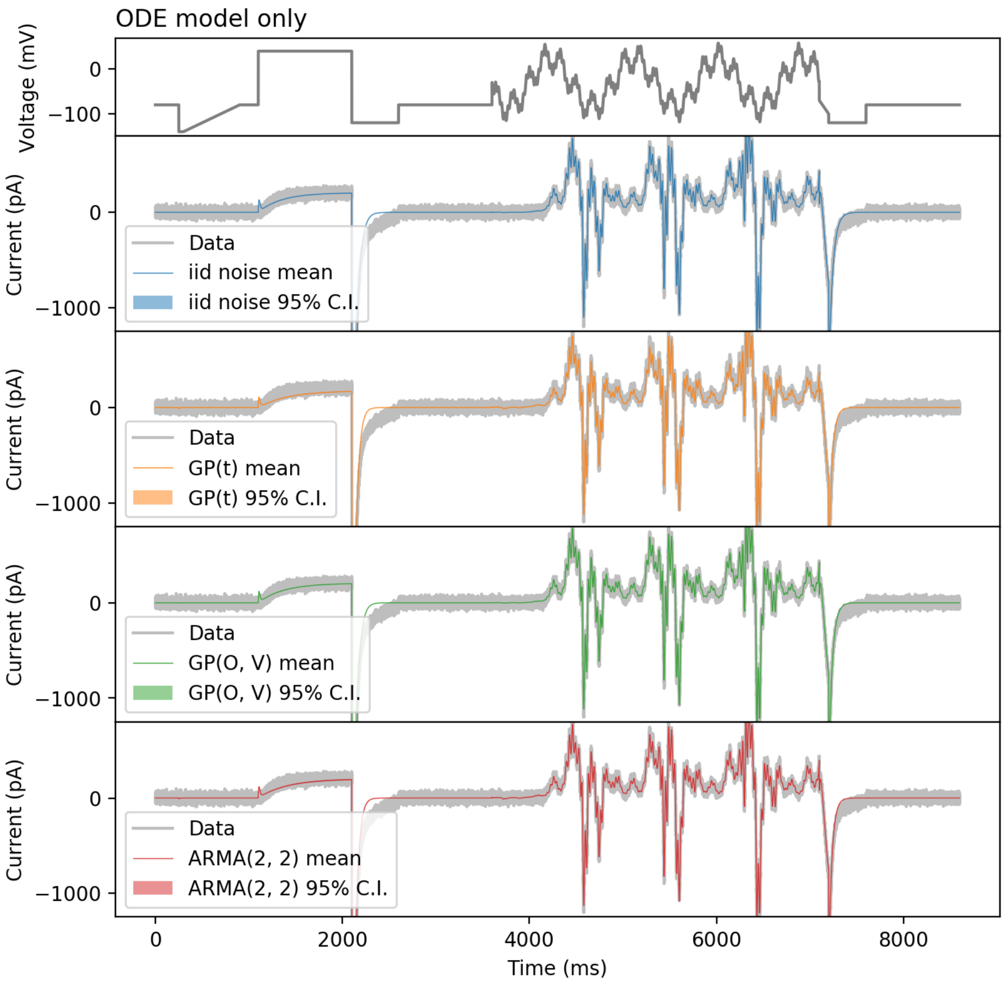

Figure S13. Fitting of the ODE model of Model A, using different discrepancy models: no discrepancy (i.i.d. noise), $\mathrm{GP}(t)$, $\mathrm{GP}(O, V)$, and $\mathrm{ARMA}(2, 2)$. The voltage clamp protocol for calibration is the sinusoidal protocol [10].

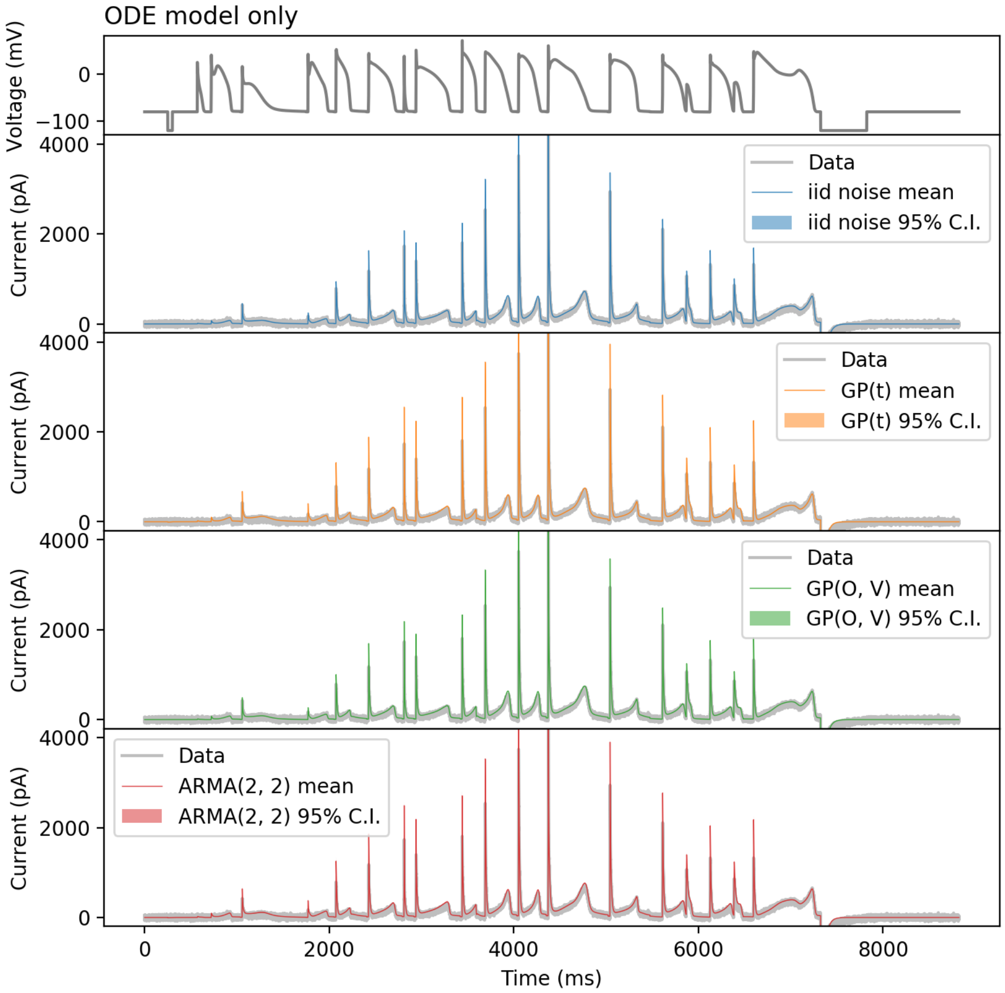

Figure S14. Predictions of the ODE model of Model A, using different discrepancy models: no discrepancy (i.i.d. noise), $\mathrm{GP}(t)$, $\mathrm{GP}(O, V)$, and $\mathrm{ARMA}(2, 2)$. The voltage clamp protocol for calibration is the action potential series protocol [10].

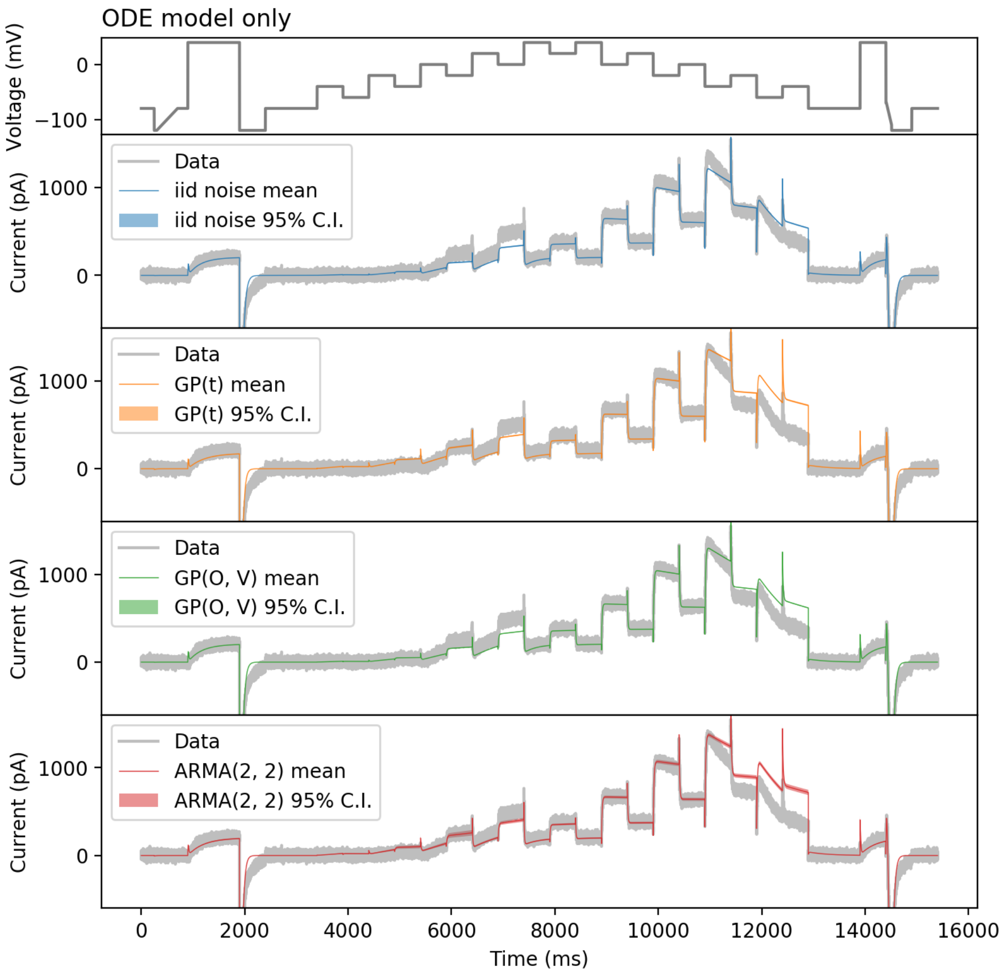

Figure S15. Predictions of the ODE model of Model A, using different discrepancy models: no discrepancy (i.i.d. noise), $\mathrm{GP}(t)$, $\mathrm{GP}(O, V)$, and $\mathrm{ARMA}(2, 2)$. The voltage clamp protocol for calibration is the staircase protocol [11].

(b) Model B

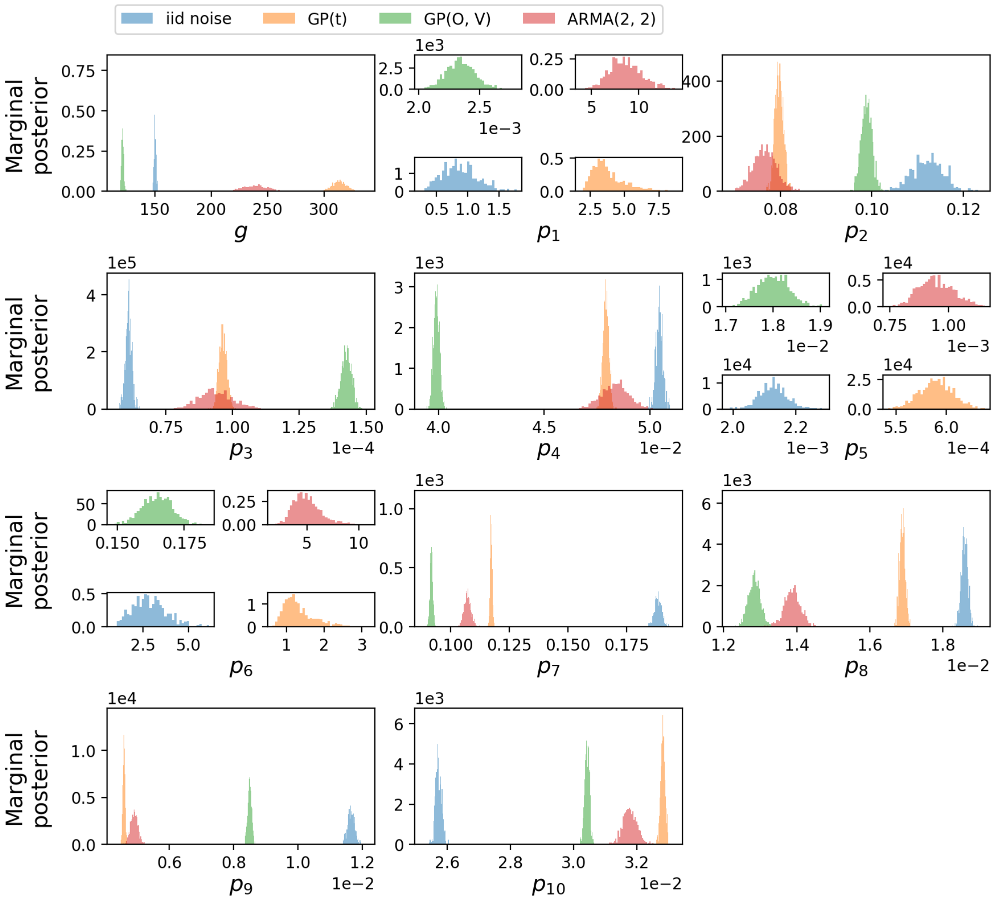

Figure S16. Model B inferred marginal posterior distributions for the conductance, g, and kinetic parameters $p_1, \ldots, p_{10}$ (a list of parameters referring to $A_{i,j}$ and $B_{i,j}$ in Eq. (3.5)) with different discrepancy models: i.i.d. noise (blue), $\mathrm{GP}(t)$ (orange), $\mathrm{GP}(O, V)$ (green), and $\mathrm{ARMA}(2, 2)$ (red).

(i) Model B: Full model predictions

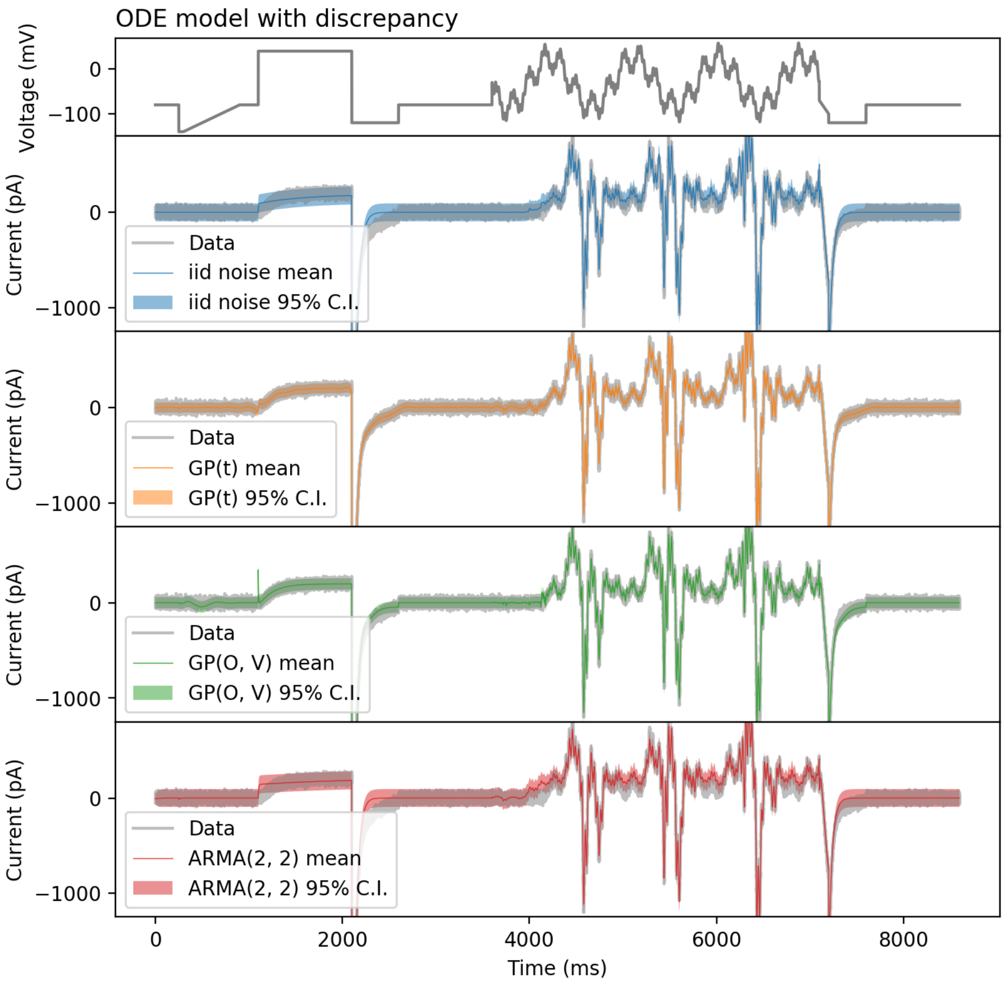

Figure S17. Model B fitting results with different discrepancy models: i.i.d. noise, $\mathrm{GP}(t)$, $\mathrm{GP}(O, V)$, and $\mathrm{ARMA}(2, 2)$. The voltage clamp protocol for calibration is the sinusoidal protocol [10]. It shows the posterior predictive with the bounds showing the 95% credible interval.

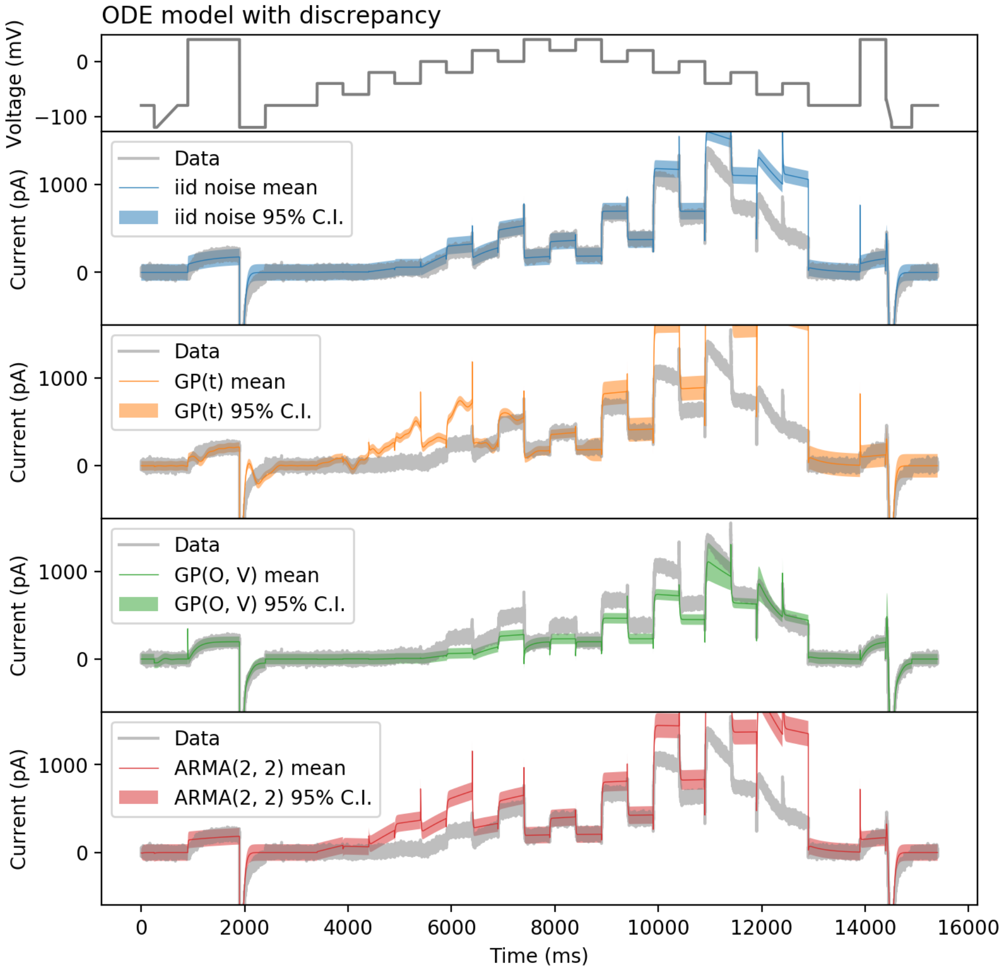

Figure S18. Model B prediction with different discrepancy models: i.i.d. noise, $\mathrm{GP}(t)$, $\mathrm{GP}(O, V)$, and $\mathrm{ARMA}(2, 2)$. The voltage clamp protocol for calibration is the staircase protocol [11]. It shows the posterior predictive with the bounds showing the 95% credible interval.

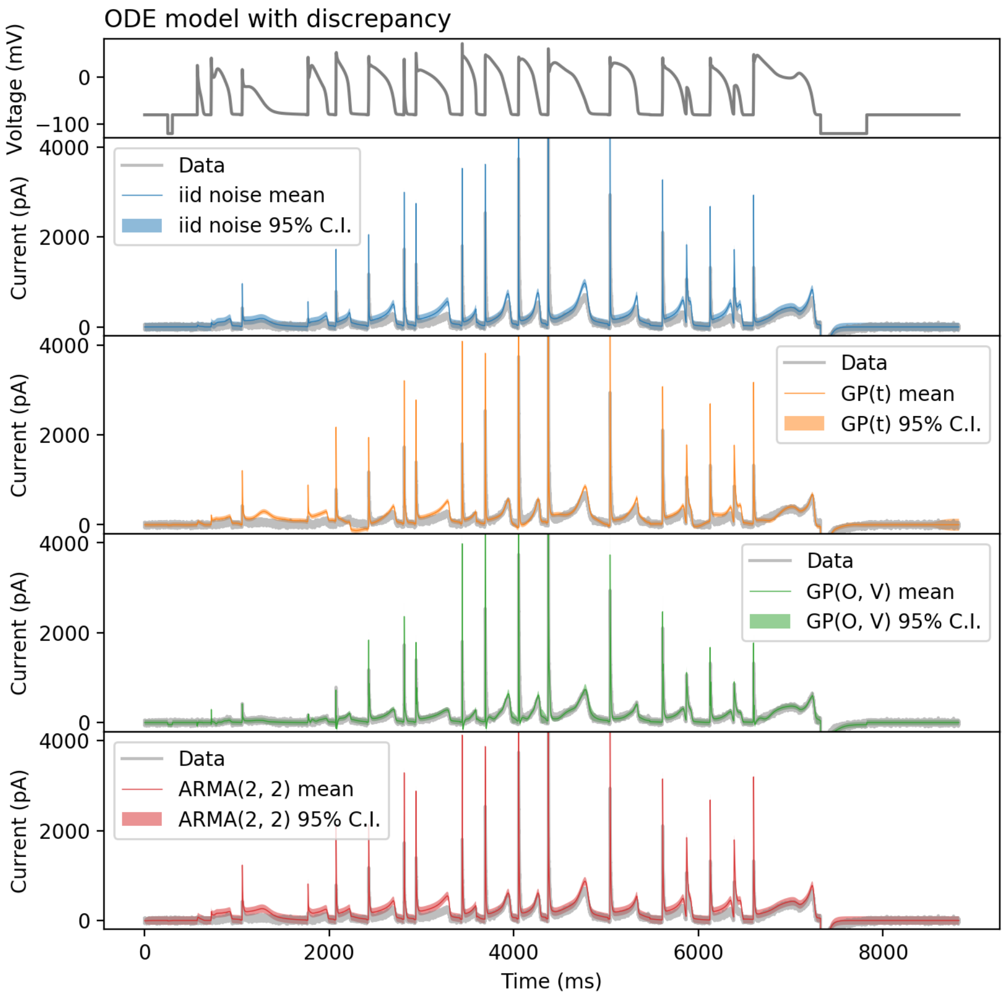

Figure S19. Model B prediction with different discrepancy models: no discrepancy (i.i.d. noise), $\mathrm{GP}(t)$, $\mathrm{GP}(O, V)$, and $\mathrm{ARMA}(2, 2)$. The voltage clamp protocol for calibration is the action potential series protocol [10].

(ii) Model B: Discrepancy predictions

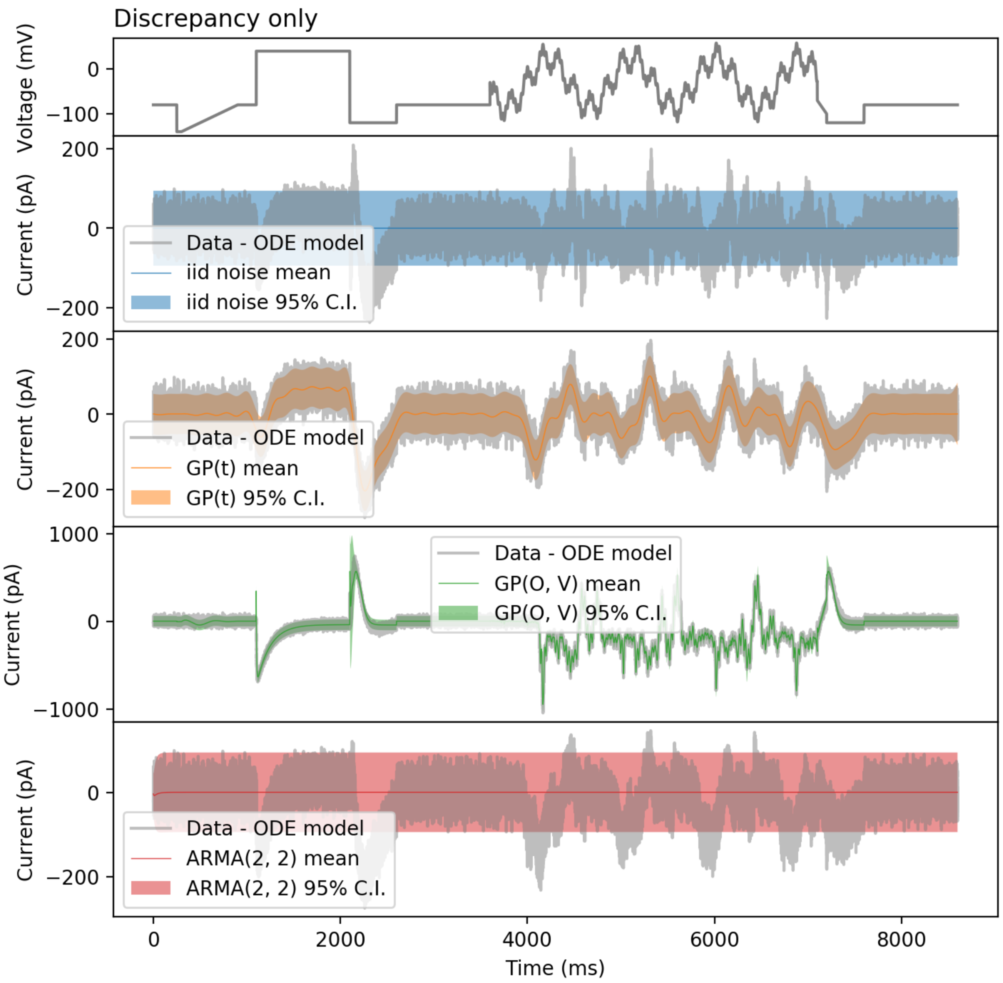

Figure S20. Model B fitting residuals of the MAP estimate accounted by different discrepancy models: no discrepancy (i.i.d. noise), $\mathrm{GP}(t)$, $\mathrm{GP}(O, V)$, and $\mathrm{ARMA}(2, 2)$. The voltage clamp protocol for calibration is the sinusoidal protocol [10].

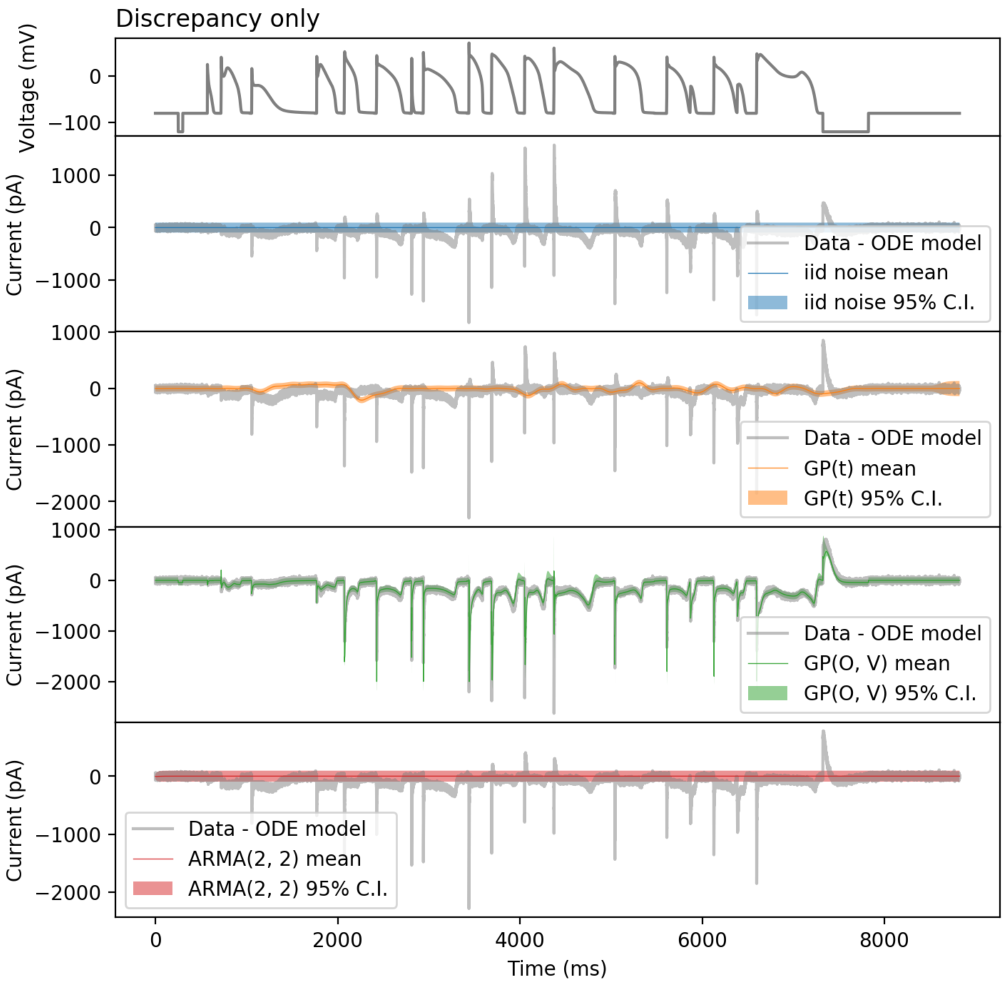

Figure S21. Model B prediction residuals of the MAP estimate accounted by different discrepancy models: no discrepancy (i.i.d. noise), $\mathrm{GP}(t)$, $\mathrm{GP}(O, V)$, and $\mathrm{ARMA}(2, 2)$. The voltage clamp protocol for calibration is the action potential series protocol [10].

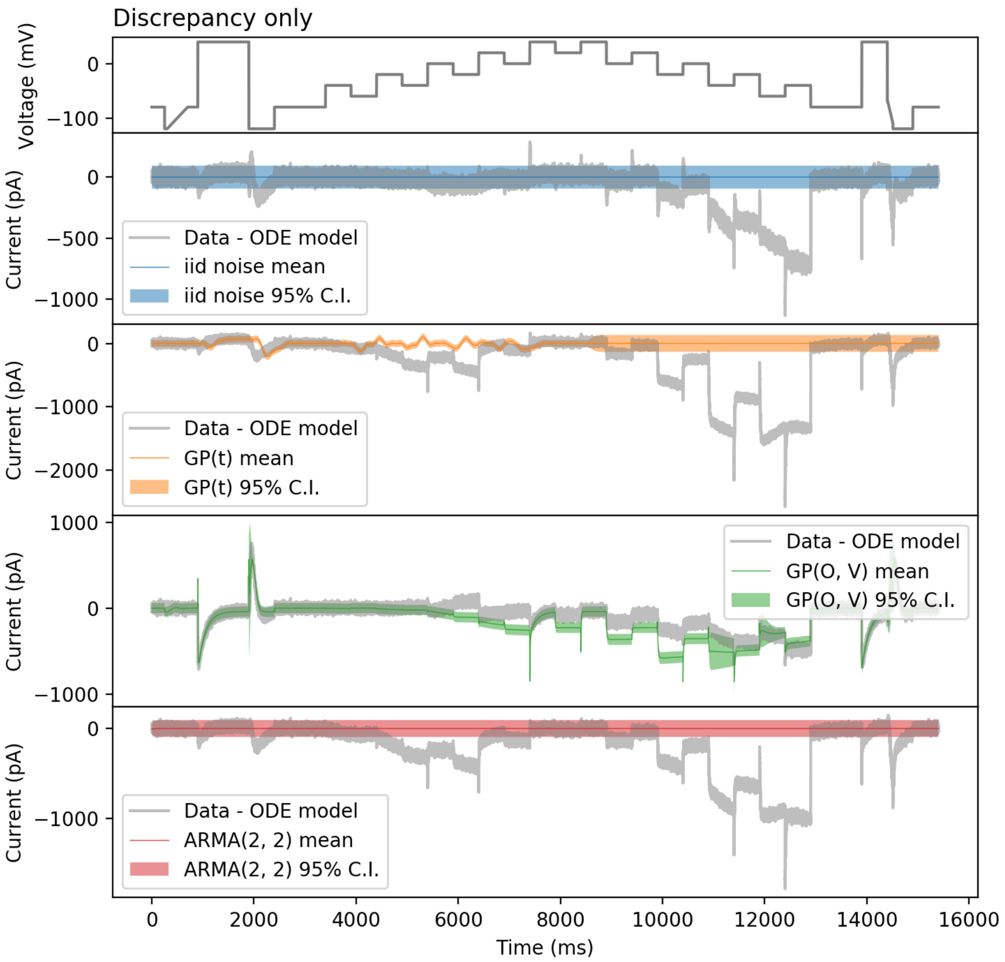

Figure S22. Model B prediction residuals of the MAP estimate accounted by different discrepancy models: no discrepancy (i.i.d. noise), $\mathrm{GP}(t)$, $\mathrm{GP}(O, V)$, and $\mathrm{ARMA}(2, 2)$. The voltage clamp protocol for calibration is the staircase protocol [11].

(iii) Model B: ODE model predictions

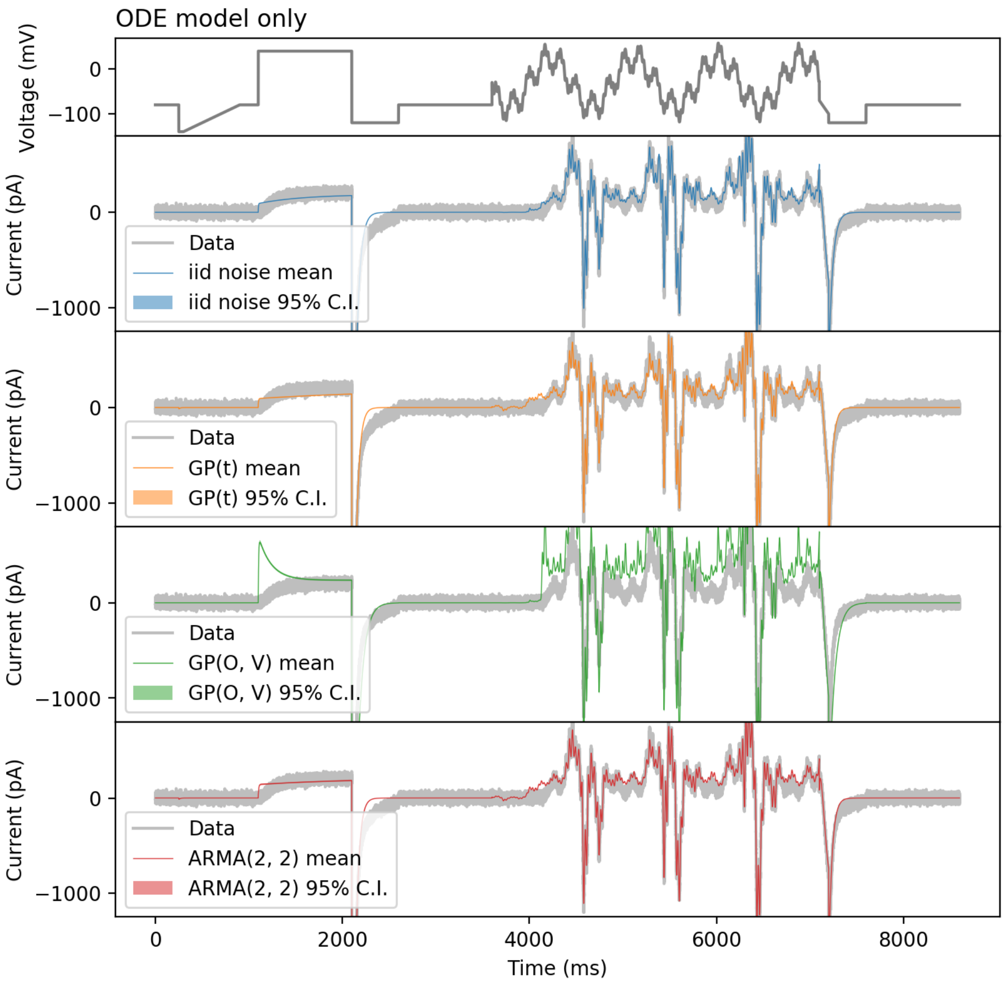

Figure S23. Fitting of the ODE model of Model B, using different discrepancy models: no discrepancy (i.i.d. noise), $\mathrm{GP}(t)$, $\mathrm{GP}(O, V)$, and $\mathrm{ARMA}(2, 2)$. The voltage clamp protocol for calibration is the sinusoidal protocol [10].

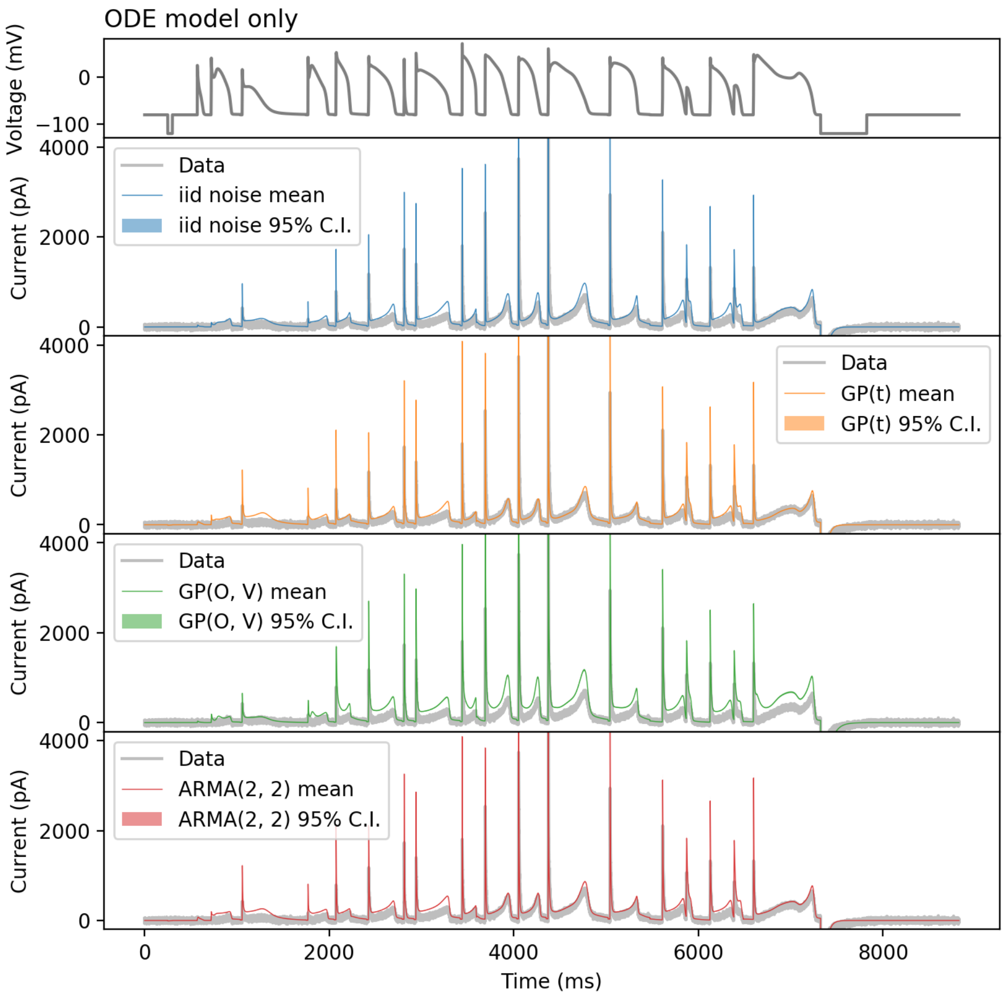

Figure S24. Predictions of the ODE model of Model B, using different discrepancy models: no discrepancy (i.i.d. noise), $\mathrm{GP}(t)$, $\mathrm{GP}(O, V)$, and $\mathrm{ARMA}(2, 2)$. The voltage clamp protocol for calibration is the action potential series protocol [10].

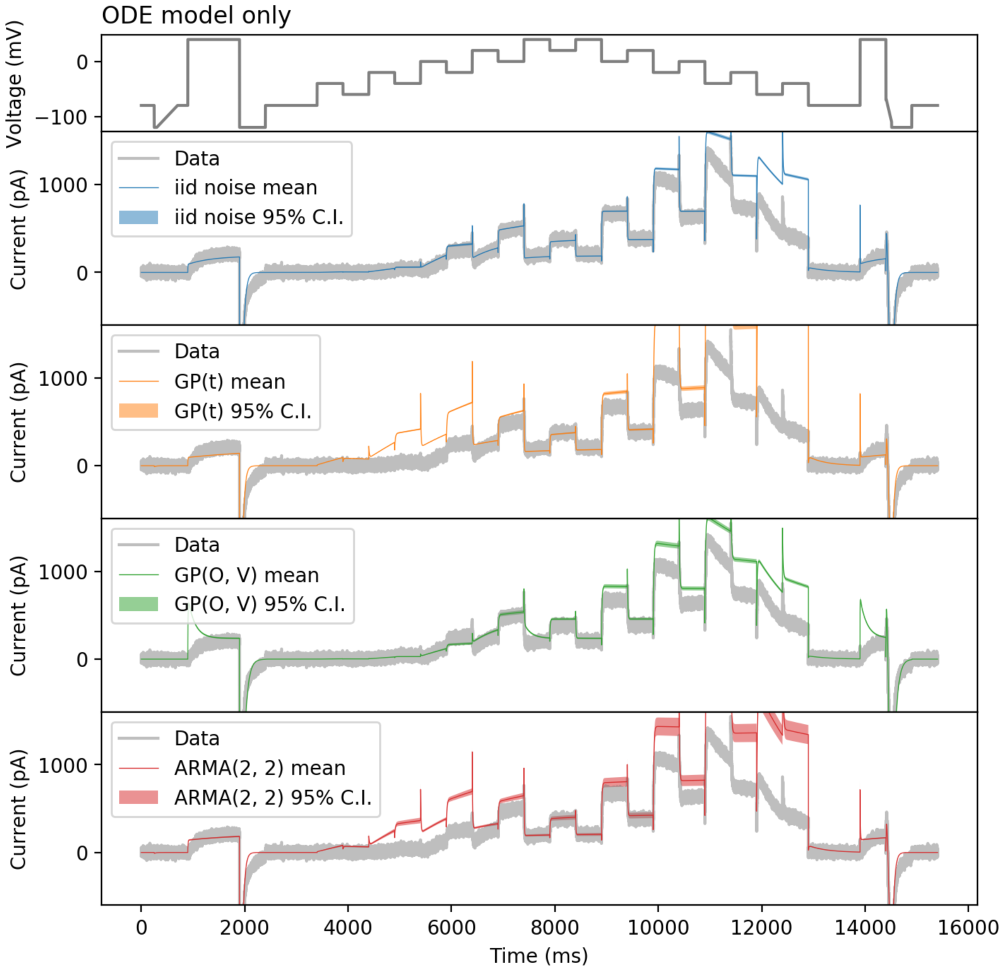

Figure S25. Predictions of the ODE model of Model B, using different discrepancy models: no discrepancy (i.i.d. noise), $\mathrm{GP}(t)$, $\mathrm{GP}(O, V)$, and $\mathrm{ARMA}(2, 2)$. The voltage clamp protocol for calibration is the staircase protocol [11].

(c) GP covariance functions: RBF, OU and Matern3/2

Full model predictions

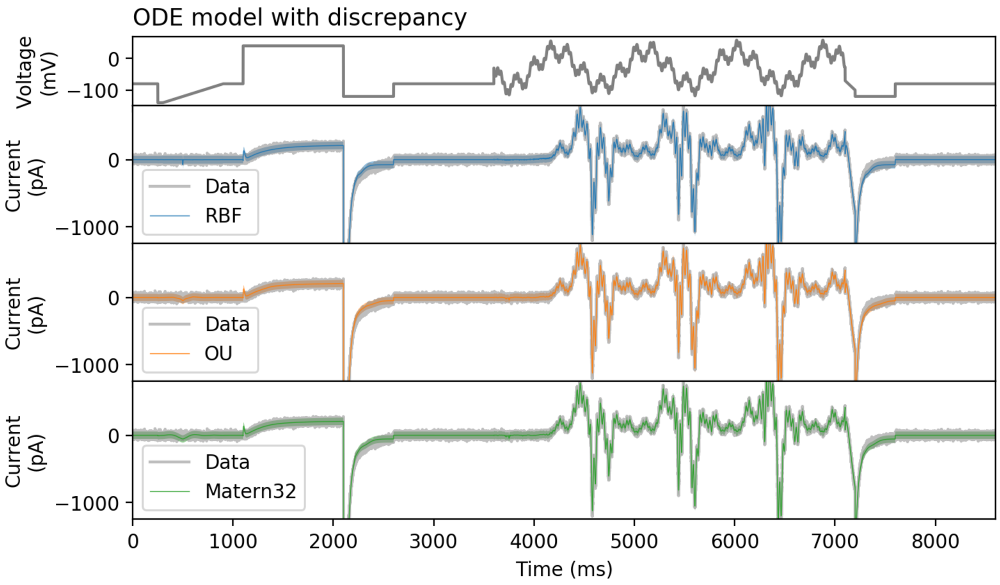

Figure S26. Model A $\mathrm{GP}(O, V)$ discrepancy model fitted with different GP covariance functions: radial basis function (RBF), Ornstein–Uhlenbeck (OU, also known as exponential covariance function), and Matérn 3/2 covariance function. The voltage clamp protocol for calibration is the sinusoidal protocol [10]. It shows the posterior predictive with the bounds showing the 95% credible interval.

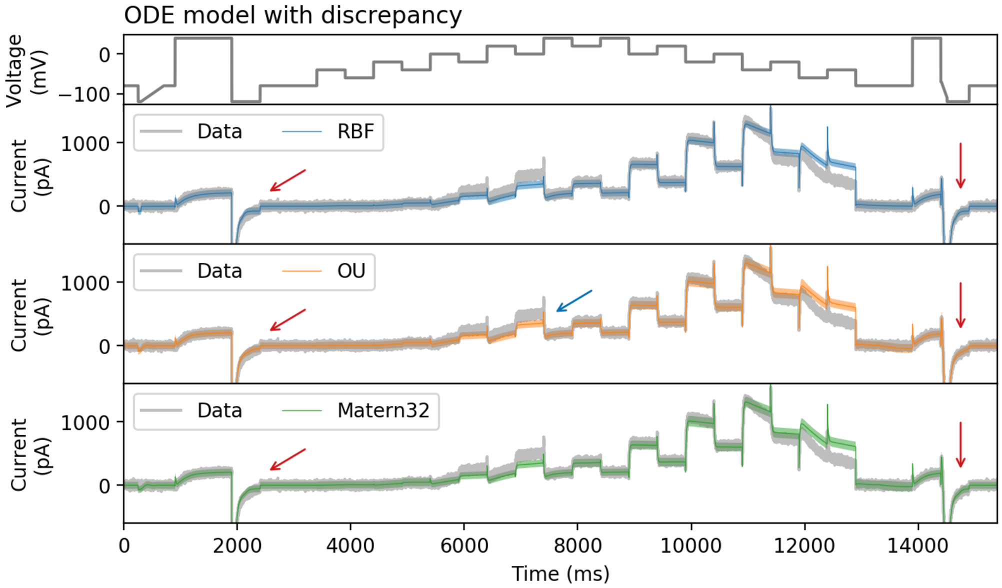

Figure S27. Model A $\mathrm{GP}(O, V)$ discrepancy model fitted with different GP covariance functions: radial basis function (RBF), Ornstein–Uhlenbeck (OU, also known as exponential covariance function), and Matérn 3/2 covariance function. The voltage clamp protocol for calibration is the staircase protocol [11]. It shows the posterior predictive with the bounds showing the 95% credible interval.

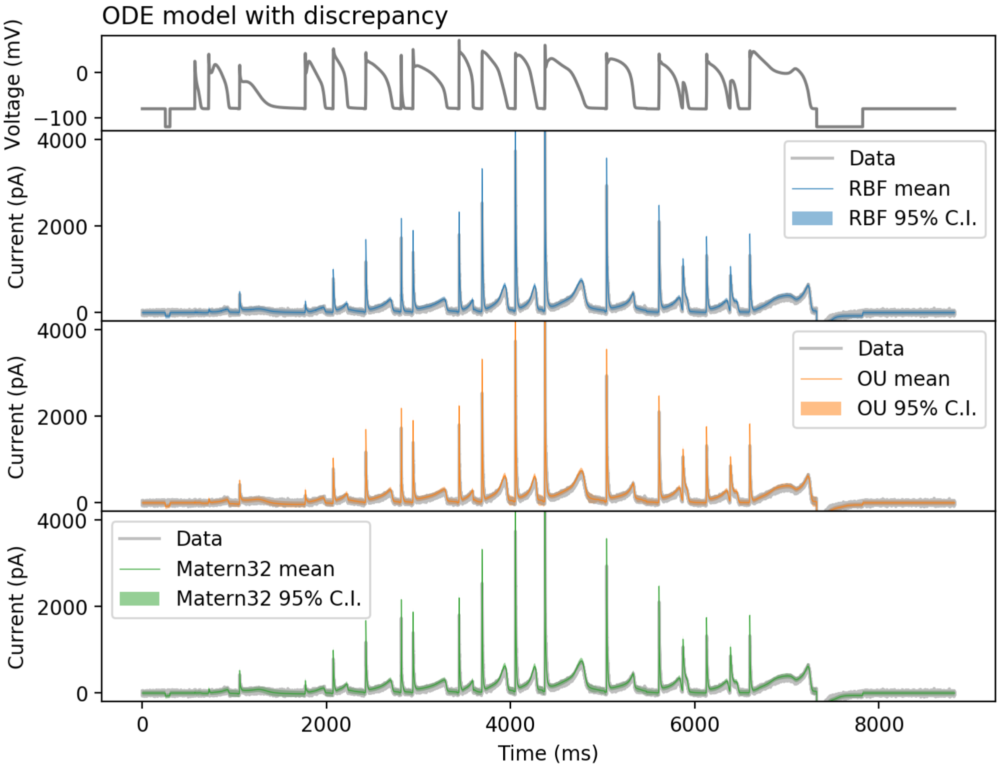

Figure S28. Model A $\mathrm{GP}(O, V)$ discrepancy model fitted with different GP covariance functions: radial basis function (RBF), Ornstein–Uhlenbeck (OU, also known as exponential covariance function), and Matérn 3/2 covariance function. The voltage clamp protocol for calibration is the action potential series protocol [10]. It shows the posterior predictive with the bounds showing the 95% credible interval.

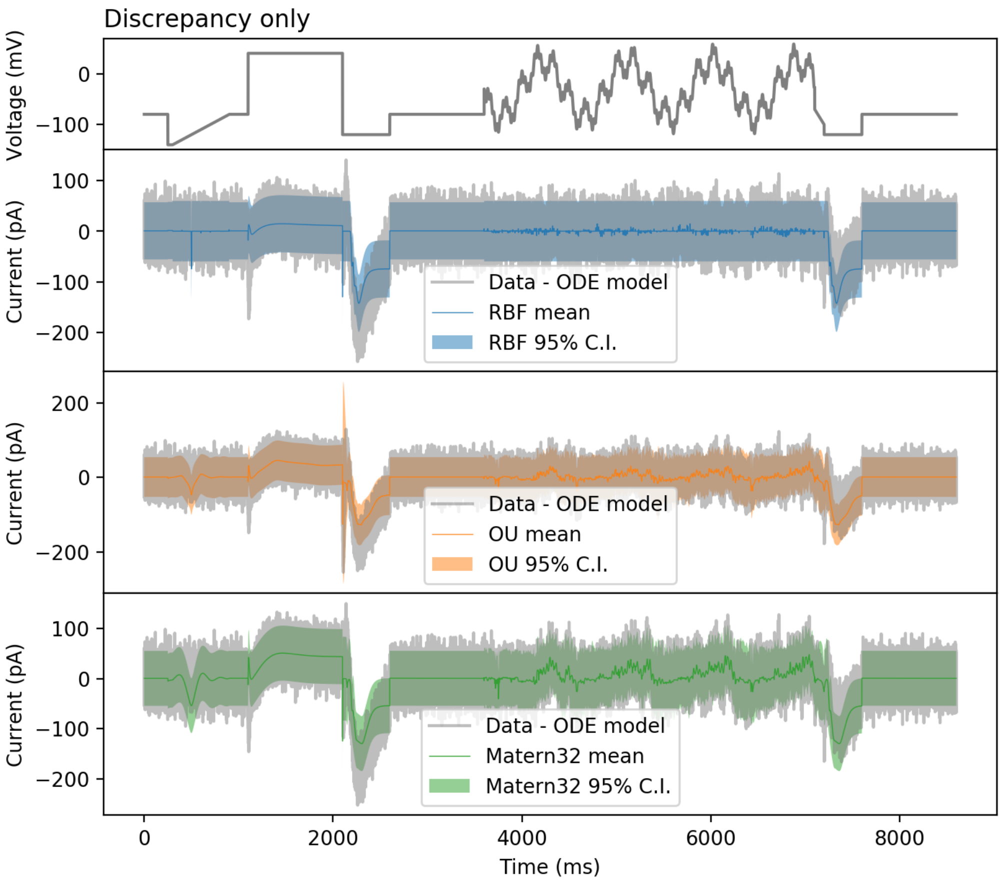

Figure S29. Model A with $\mathrm{GP}(O, V)$ discrepancy model fitting residuals of the MAP estimate accounted by different GP covariance functions: radial basis function (RBF), Ornstein–Uhlenbeck (OU, also known as exponential covariance function), and Matérn 3/2 covariance function. The voltage clamp protocol for calibration is the sinusoidal protocol [10].

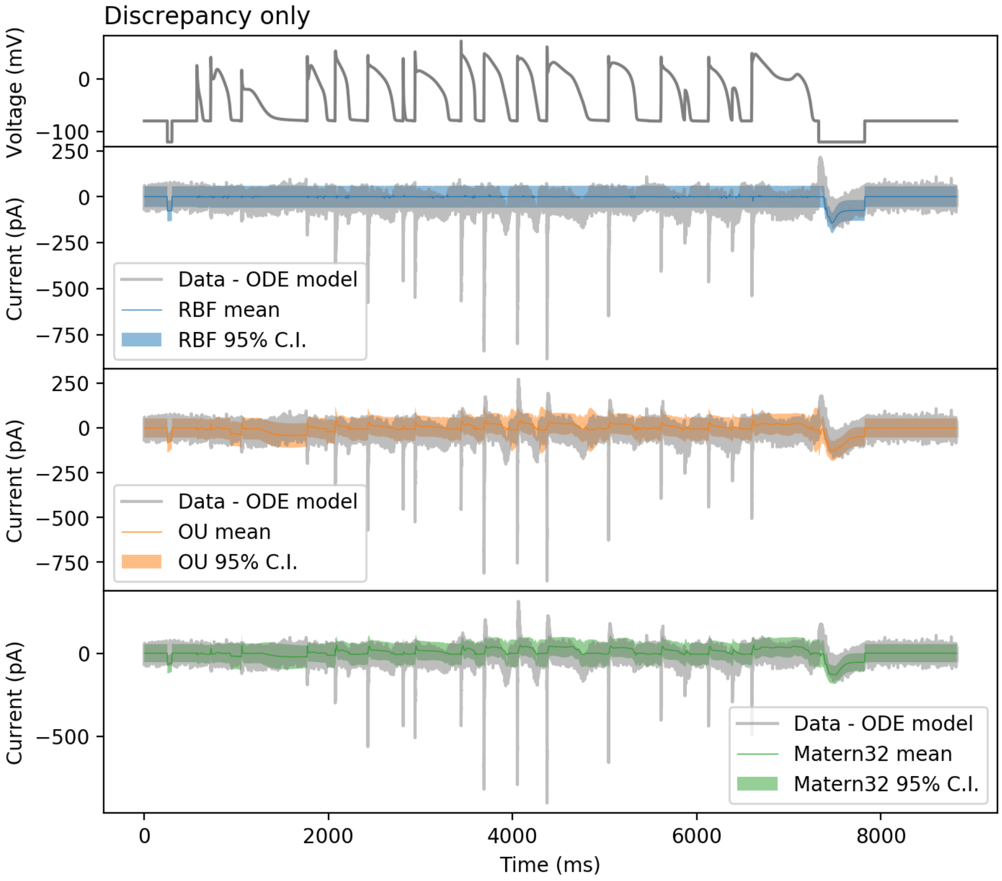

Figure S30. Model A with $\mathrm{GP}(O, V)$ discrepancy model fitting residuals of the MAP estimate accounted by different GP covariance functions: radial basis function (RBF), Ornstein–Uhlenbeck (OU, also known as exponential covariance function), and Matérn 3/2 covariance function. The voltage clamp protocol for calibration is the action potential series protocol [10].

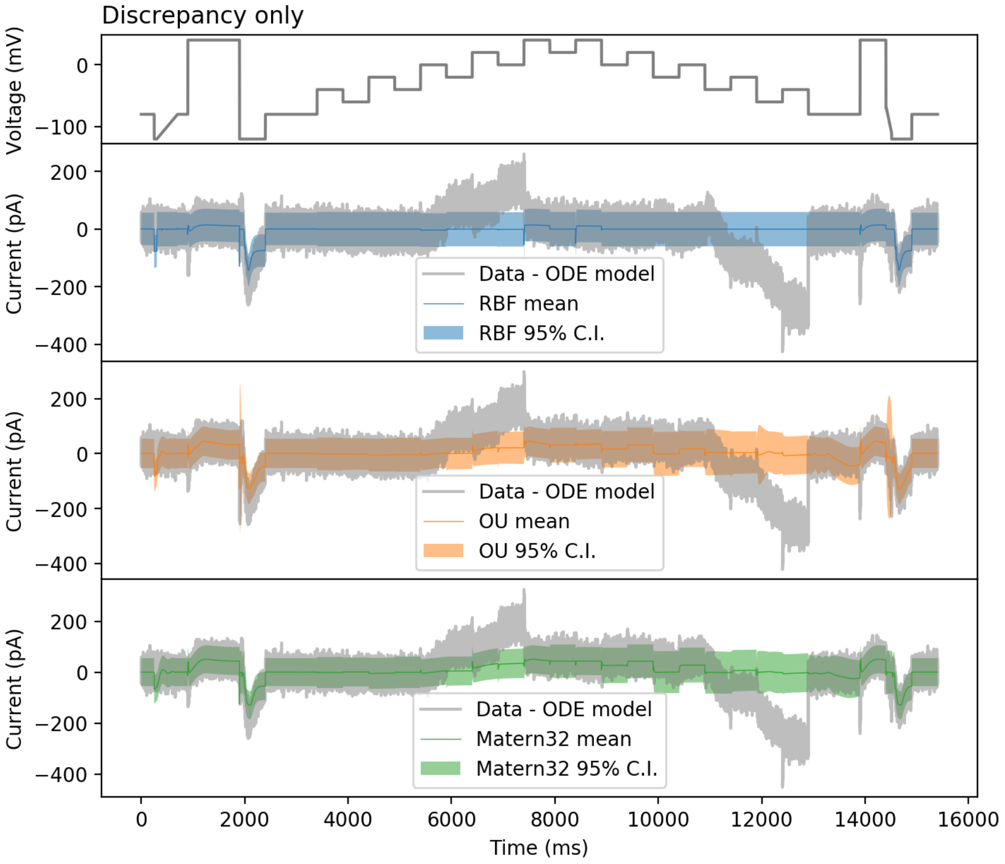

Figure S31. Model A with $\mathrm{GP}(O, V)$ discrepancy model fitting residuals of the MAP estimate accounted by different GP covariance functions: radial basis function (RBF), Ornstein–Uhlenbeck (OU, also known as exponential covariance function), and Matérn 3/2 covariance function. The voltage clamp protocol for calibration is the staircase protocol [11].

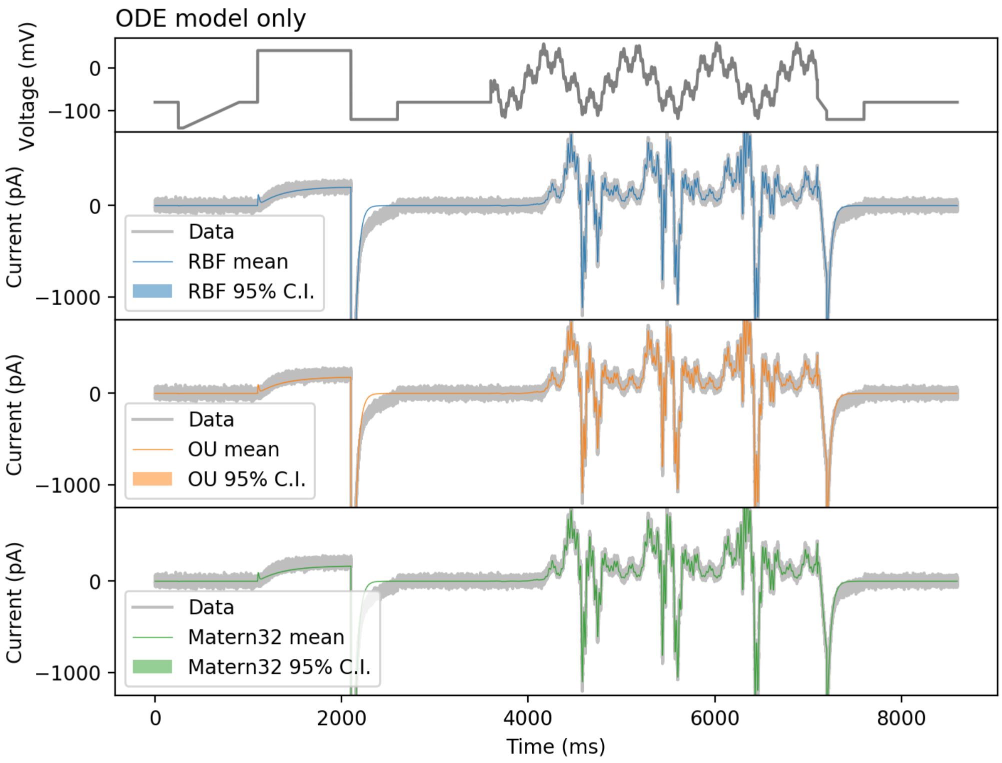

Figure S32. Fitting of the ODE model of Model A with $\mathrm{GP}(O, V)$ discrepancy model, using different GP covariance functions: radial basis function (RBF), Ornstein–Uhlenbeck (OU, also known as exponential covariance function), and Matérn 3/2 covariance function. The voltage clamp protocol for calibration is the sinusoidal protocol [10].

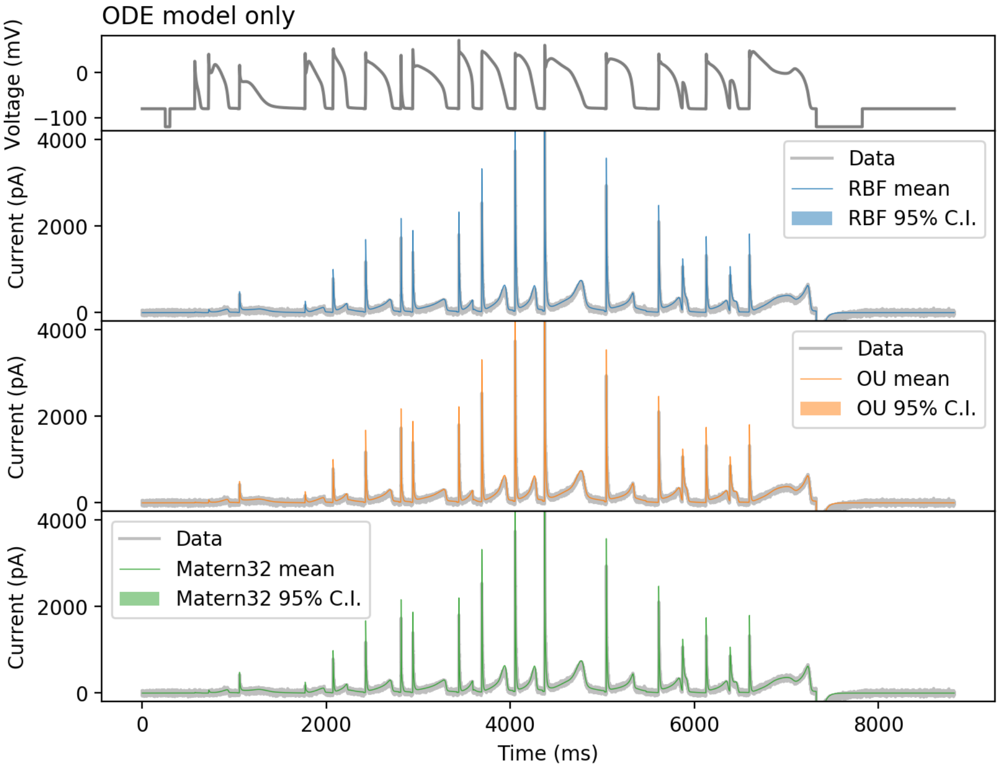

Figure S33. Fitting of the ODE model of Model A with $\mathrm{GP}(O, V)$ discrepancy model, using different GP covariance functions: radial basis function (RBF), Ornstein–Uhlenbeck (OU, also known as exponential covariance function), and Matérn 3/2 covariance function. The voltage clamp protocol for calibration is the action potential series protocol [10].

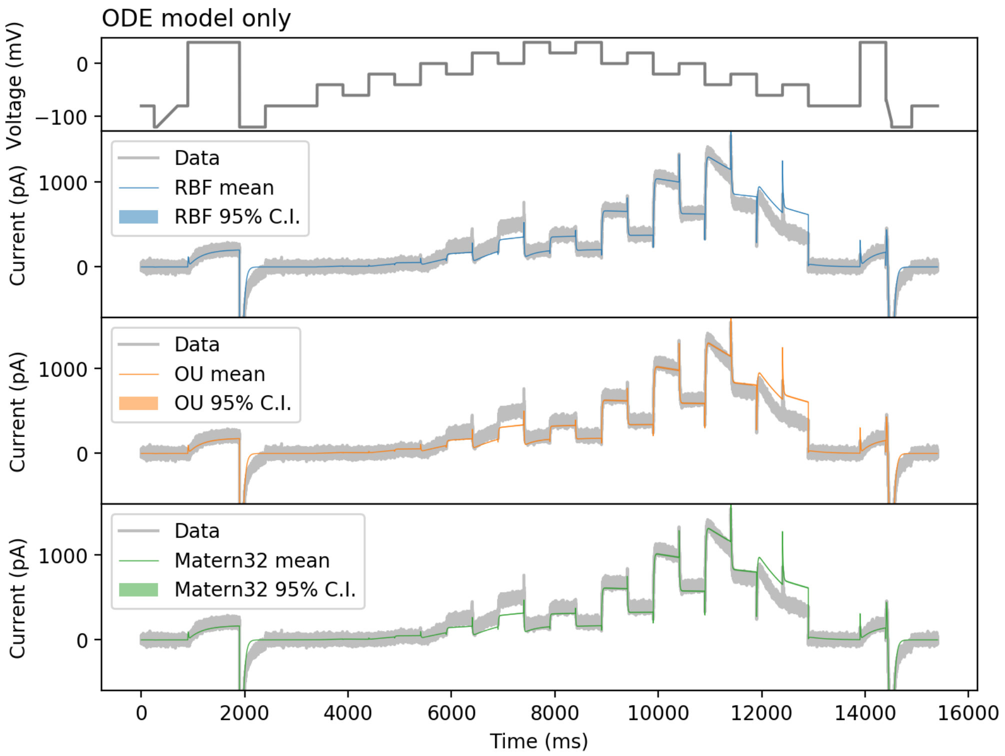

Figure S34. Fitting of the ODE model of Model A with $\mathrm{GP}(O, V)$ discrepancy model, using different GP covariance functions: radial basis function (RBF), Ornstein–Uhlenbeck (OU, also known as exponential covariance function), and Matérn 3/2 covariance function. The voltage clamp protocol for calibration is the staircase protocol [11].

References

1. K. H. Ten Tusscher, D. Noble, P.-J. Noble, and A. V. Panfilov, “A model for human ventricular tissue,” *American Journal of Physiology-Heart and Circulatory Physiology*, vol. 286, no. 4, pp. H1573–H1589, 2004.
2. M. Fink, D. Noble, L. Virag, A. Varro, and W. R. Giles, “Contributions of HERG K+ current to repolarization of the human ventricular action potential,” *Progress in biophysics and molecular biology*, vol. 96, no. 1-3, pp. 357–376, 2008.
3. M. C. Kennedy and A. O’Hagan, “Bayesian calibration of computer models,” *Journal of the Royal Statistical Society: Series B (Statistical Methodology)*, vol. 63, no. 3, pp. 425–464, 2001.
4. C. Rasmussen and C. Williams, *Gaussian processes for machine learning*. MIT Press, 2006.
5. J. Quiñonero-Candela and C. E. Rasmussen, “A unifying view of sparse approximate gaussian process regression,” *Journal of Machine Learning Research*, vol. 6, no. Dec, pp. 1939–1959, 2005.
6. E. Snelson and Z. Ghahramani, “Sparse gaussian processes using pseudo-inputs,” in *Advances in Neural Information Processing Systems*, pp. 1257–1264, 2006.
7. J. Durbin and S. J. Koopman, *Time series analysis by state space methods*. Oxford university press, 2012.
8. W. Wei, *Time Series Analysis: Univariate and Multivariate Methods, 2nd edition, 2006*. Pearson Addison Wesley, 2006.
9. J. Marriott, N. Ravishanker, A. Gelfand, and J. Pai, “Bayesian analysis of arma processes: Complete sampling-based inference under exact likelihoods,” *Bayesian analysis in statistics and econometrics*, pp. 243–256, 1996.
10. K. A. Beattie, A. P. Hill, R. Bardenet, Y. Cui, J. I. Vandenberg, D. J. Gavaghan, T. P. De Boer, and G. R. Mirams, “Sinusoidal voltage protocols for rapid characterisation of ion channel kinetics,” *The Journal of physiology*, vol. 596, no. 10, pp. 1813–1828, 2018.
11. C. L. Lei, M. Clerx, D. J. Gavaghan, L. Polonchuk, G. R. Mirams, and K. Wang, “Rapid characterisation of hERG channel kinetics I: using an automated high-throughput system,” *Biophysical Journal*, vol. 117, pp. 2438–2454, 2019.
12. C. L. Lei, M. Clerx, K. A. Beattie, D. Melgari, J. C. Hancox, D. J. Gavaghan, L. Polonchuk, K. Wang, and G. R. Mirams, “Rapid characterisation of hERG channel kinetics II: temperature dependence,” *Biophysical Journal*, vol. 117, pp. 2455–2470, 2019.
13. M. Girolami, “Bayesian inference for differential equations,” *Theoretical Computer Science*, vol. 408, no. 1, pp. 4–16, 2008.
